# Escape Intern Orientation! — A Capstone and Team Building Activity for New EM Interns

**DOI:** 10.5070/M5.52158

**Published:** 2026-04-30

**Authors:** Megan Rivera, Meredith Thompson

**Affiliations:** * Duke University School of Medicine, Department of Emergency Medicine, Durham, NC; ^ University of Florida School of Medicine, Department of Emergency Medicine, Gainesville, FL

## Abstract

**Audience:**

This activity is designed as a team building capstone exercise for new interns to review important content from intern orientation at an emergency medicine residency program.

**Introduction:**

Medical students matriculate into residency with various backgrounds, medical knowledge base, and personalities. As they orient themselves into their respective specialties, interns are commonly taught using lectures and slide decks with varied and unclear effectiveness.^1^,^2^ It has been recommended that intern orientation should incorporate active learning, such as simulation as well as a collaborative environment to bolster retention and encourage teamwork.^1^,^3^ Escape rooms are becoming increasingly popular in medical education as an adjunct to traditional lecture-based learning that encourages collaboration amongst learners.^4^ We embraced this modality to create a capstone and teambuilding experience for our new interns that was delivered at the completion of our intern orientation activities. Topics taught in our intern orientation include resuscitation of the critically ill patient, airway management, acute coronary syndromes, EKG reading, basic ultrasound, stroke care, and foundational pediatric topics. In this activity, an escape room is used to recall and apply topics learned during the emergency medicine intern orientation, while also promoting teamwork and camaraderie amongst a new intern cohort.

**Educational Objectives:**

By the end of this small group exercise, learners will be able to:

1. Identify first, second, and third-degree heart block on a 12-lead ECG.
2. Recognize STEMI pattern on a 12-lead ECG.
3. Categorize appropriate images that make up an EFAST exam for a trauma patient.
4. Recall the proper management of a tension pneumothorax.
5. Identify an organized approach to emergency department rapid-sequence intubation (RSI).
6. Recognize acute otitis media (AOM).
7. Locate the appropriate antibiotic and pediatric dose to treat acute otitis media via the *Harriet Lane Handbook*.
8. Demonstrate how to apply evidence-based guidelines to a clinical case of neonatal pediatric fever.
9. Recall common clinical findings of basilar skull fracture.
10. Identify important concepts in the management of stroke syndromes.
11. Recognize vital sign abnormalities that could indicate sepsis.
12. Review important concepts related to the management of septic patients.

**Educational Methods:**

The use of game-based learning or gamification has become increasingly popular within medical education as a form of active learning.^5^ There is a growing body of literature among health care professionals highlighting improvement in participants’ skills and learning through games as well as the ability of this method in encouraging peer education and socialization.^6^–^8^ Escape rooms are a form of game-based learning employing various puzzles and settings specifically designed to meet educational objectives in an active learning environment.^9^ Recently, literature has shown that escape rooms can be an instrument to foster relationships amongst co-workers in addition to facilitating improved learning outcomes via active learning.^10^

We developed an escape room to review and solidify important content from our intern orientation as well as to encourage teamwork and camaraderie on the last day of orientation. The intern class was divided into three teams, each with four participants. Each team selected their own team name and were informed during a pre-briefing that they would only be able to escape intern orientation by completing all six stations. A facilitator at each station timed the teams. The team with the fastest time was deemed the winner which was announced after a short debrief of the activity.

**Research Methods:**

All interns who participated in the escape room completed an anonymous online survey after the activity. The survey was designed to solicit feedback on the effectiveness of the activity in reviewing material taught during orientation as well as overall satisfaction with the escape room experience measured as a recommendation to continue the activity in the future.

**Results:**

All twelve (100%) interns that attended the escape room completed the post participation survey. Ninety-two percent (11/12 interns) thought the activity was moderately to extremely effective in reviewing the content delivered during intern orientation and 100% (12/12 interns) recommended we continue the escape intern orientation activity for future classes. Representative free response comments soliciting general feedback on the activity included “Great event, do it again. Very interactive and fun.” As well as “Great activity to tie in all that we learned this month. Very creative and clever! Also, a great team building opportunity as well.”

**Discussion:**

Intern orientation is a necessary, high-yield time for new doctors to learn foundational specialty and hospital specific topics.^11^ As educators are being challenged to move away from traditional classroom lecturing, gamification has emerged within medical education as an active learning tool to increase engagement, promote team building, provide immediate feedback, and make content more interesting.^12^ Escape rooms fall within a constructivist theory of learning because participants are challenged to incorporate existing knowledge to draw conclusions, ultimately unlocking their “escape.”^13^

Further, intern orientation is also a time of social gathering and team building. Because camaraderie is connected to wellbeing, it is highly valued in emergency medicine residencies.^14^ When interns matriculate into their residencies, social interactions are encouraged among the group. In our escape room activity, gamification is used to enhance social interaction in a way that encourages relationship building and teamwork. This is important as cohesiveness amongst the resident group is a key factor for their success in residency.^15^

**Topics:**

Intern, emergency medicine, heart blocks, extended focused assessment with sonography in trauma (EFAST), rapid sequence intubation, pediatric fever, stroke, sepsis.

## USER GUIDE

**Table:** 

List of Resources:	
Abstract	1
User Guide	3
Small Groups Learning Materials	8
Appendix A: Pre-Brief PowerPoint	29
Appendix B: Supplemental Images & Documents PowerPoint	30
Appendix C: Materials Master Checklist	31
Appendix D: Participant Sheet	33
Appendix E: Wrap-Up PowerPoint	35

**Learner Audience:**

Emergency medicine interns.

**Time Required for Implementation:**

Prepare materials at least 3 weeks in advance of the activity. Allow 2–3 hours for set up and 2–3 hours for the activity. A “practice run” with facilitators prior to the activity is strongly encouraged.

**Recommended Number of Learners Per Instructor:**

3–5

**Topics:**

Intern, emergency medicine, heart blocks, extended focused assessment with sonography in trauma (EFAST), rapid sequence intubation, pediatric fever, stroke, sepsis.

**Objectives:**

By the end of this small group exercise, learners will be able to:

1. Identify first, second, and third-degree heart block on a 12-lead ECG.
2. Recognize STEMI pattern on a 12-lead ECG.
3. Categorize appropriate images that make up an EFAST exam for a trauma patient.
4. Recall the proper management of a tension pneumothorax.
5. Identify an organized approach to emergency department rapid-sequence intubation.
6. Recognize acute otitis media (AOM).
7. Locate the appropriate antibiotic and pediatric dose to treat acute otitis media via the *Harriet Lane Handbook*.
8. Demonstrate how to apply evidence-based guidelines to a clinical case of neonatal pediatric fever.
9. Recall common clinical findings of basilar skull fracture.
10. Identify important concepts in the management of stroke syndromes.
11. Recognize vital sign abnormalities that could indicate sepsis.
12. Review important concepts related to the management of septic patients.

### Linked objectives and methods

Our goals for developing this educational activity were two-fold. First, we wanted to review and solidify important content taught during intern orientation. Second, we wanted to create a unique capstone experience to facilitate teamwork and camaraderie amongst the new intern cohort. Gamification in the form of an escape room represented the best choice to achieve these goals given the inherent characteristics of this modality and how it aligned with our constructivist conceptual framework for the activity.

It is becoming increasingly known within medical education literature that active, problem-based learning is more effective than passive, lecture-based learning.^16^–^19^ Active, problem-based learning falls within a constructivist theory of learning, which is thought to be superior among other learning theories.^20^ In constructivism, participants acquire new knowledge through application of previously known concepts.^13^ This framework asserts that activation of prior knowledge is critical to the learning process, as learners interpret new experiences based on what they already know.^20^ This perspective also shifts the instructional strategy to focus on the learner, requiring active engagement (through gamification via an escape room in our activity) to construct their own knowledge base. Escape rooms have been utilized among various U.S. residencies including thoracic surgery, radiology, and emergency medicine with favorable outcomes.^21^–^23^

Additionally, we found the escape room modality was best to encourage socialization and teamwork among the new intern cohort.^8^ It has been found that escape room activities additionally increase workplace social capital scores.^14^ This important construct is a measure of the network of relationships amongst co-workers which enables the workplace to function effectively as well as refers to the mutual trust and connectedness of a work group that can foster a sense of support and belonging.^10^ This is key given the importance of developing relationships and connectedness early in residency to thrive in a new educational environment.^15^

Educational Objective (EO) 1: Identify first, second, and third-degree heart block on a 12-lead ECG. This objective is met during scenario 1. Participants enter the room and see a riddle prominently displayed describing each type of heart block but with the answers blank. ECGs are found in various places around the room. The type of block for each ECG must be correctly identified to unlock a clue to find the next puzzle.

EO 2: Recognize STEMI pattern on a 12-lead ECG. This is also accomplished in scenario 1. Participants identify the word COAT from the previous puzzle and find a white coat with a folded ECG in the pocket. This ECG demonstrates a lateral STEMI. ST elevations in leads 1, avL, 5, 6 will need to be identified to create a code (1VL56) to unlock a box with your directions to proceed to the next room.

EO 3: Categorize appropriate images that make up an EFAST exam for a trauma patient. This is addressed in scenario 2. A clinical case describing a trauma patient is prominently displayed when participants enter the room. Various ultrasound images are scattered on a desk which must be organized into the correct sequence for an EFAST exam. Once correctly identified (all are present except the lung window), the images spell out the word LAMP that leads to the next puzzle.

EO 4: Recall the proper management of a tension pneumothorax. This is also accomplished in scenario 2. A picture of a barcode sign will be found underneath the lamp. A riddle is on the opposite side. A blacklight found in the room must be used to illuminate a code to a lock box where a 14-gauge needle is found. The participant must pop balloons with the needle until they discover the clue inside the balloon that appropriately manages the pneumothorax and provides the clue to lead to the next station.

EO 5: Identify an organized approach to emergency department rapid-sequence intubation. This objective is met with scenario 3. Participants must identify the correct sequence of the 7 Ps of RSI amongst several distractors. Once done, the pictures will provide a clue to use to unlock a box of airway equipment. This must be used to find a clue in an intubation mannequin’s airway to proceed to the next station.

EO 6: Recognize acute otitis media and EO 7: Locate the appropriate antibiotic and pediatric dose to treat acute otitis media via the *Harriet Lane Handbook*.

These objectives are both met in scenario 4. Participants must identify a picture of acute otitis media to be able to use the *Harriet Lane Handbook* to find the appropriate treatment that gives a clue to unlock a box with materials for the next puzzle.

EO 8: Demonstrate how to apply evidence-based guidelines to a clinical case of neonatal pediatric fever. This objective is also achieved in scenario 4. Participants are provided with a clinical case of pediatric fever and directed by clues to a paper copy of the REVISE II Clinical Practice Guideline for Febrile infants.^24^ They must apply these guidelines to the case and determine the correct order of management steps to treat the theoretical patient. Once the correct order is identified, this provides a code to a box to be opened with instructions to proceed to the next puzzle.

EO 9: Recall common clinical findings of basilar skull fracture. This is accomplished during scenario 5. Participants must deduce the clinical finding of raccoon eyes from a picture of a raccoon with “googly eyes.” They must then identify with a black light flashlight that the temporal bone of a skull in the room has a code with invisible ink. This code opens a lock box with the next puzzle.

EO 10: Identify important concepts in the management of stroke syndromes. Scenario 5 also accomplishes this objective. Participants unlock a box that has a clinical picture of racoon eyes and a quiz cut into pieces. They must then assemble and complete the quiz, which addresses several different aspects of stroke care. The letters by each correct answer then are rearranged to provide a clue of which room to proceed to next.

EO 11: Recognize vital sign abnormalities that could indicate sepsis. Scenario 6 accomplishes this objective. Participants are presented with a clinical case, but vital signs are missing from the nursing home report. They then must deduce that bolded letters found in the case correspond to elements found on a periodic table hanging in the room and that the anatomic numbers are the vital sign data. Participants must recognize based on the context of the case and the newly discovered vital signs that the patient is possibly septic. Once this data is found it unlocks a box with materials for the next puzzle.

EO 12: Review important concepts related to the management of septic patients. This objective is also addressed in scenario 6. Participants are provided another puzzle where they must differentiate risk of community vs. hospital acquired infections based on the clinical case and choose the correct antibiotics for empiric coverage. These antibiotics will be available in a labeled syringe amongst other distractors. Participants must identify the correct antibiotics and deduce that the number of ccs in the syringe makes the code to open the box to complete the room.

### Recommended pre-reading for facilitator

The following are supplemental materials to serve as pre-reading or to use as a reference for facilitators:

1. Braude D. The Ten Ps of Rapid Sequence Intubation. *Emerg Med News*. 2007;29(1):8. doi:10.1097/01.EEM.0000264634.15897.25
2. Pantell RH, Roberts KB, Adams WG, et al. Evaluation and management of well-appearing febrile infants 8 to 60 days old. *Pediatrics*. 2021 Aug;148(2):e2021052228. https://publications.aap.org/pediatrics/article/148/2/e2021052228/179783/Evaluation-and-Management-of-Well-Appearing?autologincheck=redirected

### Optional Learner Responsible Content (LRC)

1. Brady WJ, Glass III GF. Cardiac rhythm disturbances. In: Tintinalli JE, Ma O, Yealy DM, Meckler GD, Stapczynski J, Cline DM, Thomas SH. eds. *Tintinalli’s Emergency Medicine: A Comprehensive Study Guide, 9e*. McGraw-Hill Education; 2020.
2. Rowland-Fisher A, Reardon R. E-FAST (Extended Focused Assessment with Sonography in Trauma). American College of Emergency Physicians. October 19, 2021. Accessed May 5, 2022. https://www.acep.org/sonoguide/basic/fast
3. Braude D. The Ten Ps of rapid sequence intubation: *Emerg Med News*. 2007;29(1):8. doi:10.1097/01.EEM.0000264634.15897.25
4. Pantell RH, Roberts KB, Adams WG, et al. Evaluation and management of well-appearing febrile infants 8 to 60 days old. *Pediatrics*. 2021 Aug;148(2):e2021052228. doi:10.1542/peds.2021-052228
5. Go S, Kornegay J. Stroke syndromes. In: Tintinalli JE, Ma O, Yealy DM, Meckler GD, Stapczynski J, Cline DM, Thomas SH. eds. *Tintinalli’s Emergency Medicine: A Comprehensive Study Guide, 9e*. McGraw-Hill Education; 2020.
6. Puskarich MA, Jones AE. Sepsis. In: Tintinalli JE, Stapczynski J, Ma O, Yealy DM, Meckler GD, Cline DM. eds. *Tintinalli’s Emergency Medicine: A Comprehensive Study Guide, 8e*. McGraw-Hill Education; 2016.

### Small group application exercise (sGAE)

See the following attached materials for this small group exercise

- Appendix A: Pre-Brief PowerPoint
- Appendix B: Supplemental Images & Documents PowerPoint
- Appendix C: Materials Master Checklist
- Appendix D: Participant Sheet
- Appendix E: Wrap-Up PowerPoint

### Results and Tips for Successful Implementation

All interns that participated in the activity completed an anonymous online post participation survey. The response rate was 12/12 (100%). Ninety-two percent (11/12 interns) thought the activity was moderately to extremely effective in reviewing the content delivered during intern orientation and 100% (12/12 interns) recommended we continue the escape intern orientation activity for future classes. Our participants found the “pediatric fever” and “7 Ps of intubation” to be the hardest stations and the “ultrasound” and “EKG” stations were rated the most enjoyable. Free response comments soliciting general feedback on the activity were all positive: “Great event, do it again. Very interactive and fun,” as well as, “Great activity to tie in all that we learned this month. Very creative and clever! Also, a great team building opportunity as well.” And finally, “Great time, lots of fun!”

We recommend the following tips for successful implementation:

1. Ensure your escape rooms align tightly with topics discussed during intern orientation. We discovered that the neonatal fever guidelines were not covered in sufficient depth leading to participants struggling with this puzzle in the pediatric station.
2. Budget appropriate time for activity preparation and activity debrief. Because we ran out of time and had to rush through our debrief, we plan to budget extra flex time to make sure this does not occur in the future.
3. Conduct a “dress rehearsal” to ensure all components of the escape room are accounted for in advance. This is key to discover the effectiveness of your setting and to educate facilitators on their roles.
4. Encourage team names and gather props for team dress-up (the crazier the better!). This is a fun addition and adds to the team building and enjoyment of the activity.
5. Gather prizes for winners of best team name and best overall time. Our faculty donated gift cards, so all interns had the opportunity to go out and socialize further after the event.

While there is a significant amount of preparation needed, we believe that the benefits of this escape room justify the time spent based on our experience.

## Supplementary Information







## Figures and Tables

**Table:** 

**Overview:**	This escape room activity consists of six escape “rooms.” In this activity, faculty offices are used as each escape room, but other arenas may be used such as a large room divided into parts or classrooms. A pre-brief is conducted (Appendix A); then learners are divided into groups, and each group is given a clipboard, paper, and pen. The clipboard has the participant sheet (Appendix D) where the facilitator of each room records the team’s time and keeps track of their progress to ensure that they escape all rooms. A crossword puzzle is also found on this sheet and represents the final puzzle needed to complete each room to stop the time. A hint is available in a sealed envelope for each room, but using a hint comes with a 60 second penalty (found below and in Appendix B). Allow 2 hours for set up, and 2–2.5 hours for the activity and debrief. A practice run is strongly encouraged. Be sure to map out your rooms in a way that makes sense for your space. One volunteer (senior resident or faculty member) is needed for each escape room and another volunteer for circulating around all rooms, totaling 6–7 volunteer instructors. Participants are divided into 3–4 groups, and each group starts in a different station. We recommend that 2 groups start at a longer station each (Stations 4 and 6), and the other groups complete two shorter stations in sequence (Stations 1, 2, 3, or 5); groups do not have to complete stations in order. If one group completes a scenario before the next station is ready, they may wait outside the station until invited in. Six different scenarios related to core emergency medicine topics were crafted into scenarios. A debrief session should be held following the activity (Appendix E).
**Pre-brief Presentation**	Found in Appendix A
**Supplemental Images, Documents, and Hints:**	Found in Appendix B
**Materials Checklist for Entire Activity**	Found in Appendix C
**Participant Sheet:**	Found in Appendix D
**Brief Wrap Up Presentation**	Found in Appendix E

**Table:** 

*Scenario 1:*	Basic ECG Interpretation
***Estimated Time to Completion***	**Approximately 15 minutes**
**Learning Objectives:**	Identify first, second, and third-degree heart block on a 12-lead ECG. Recognize STEMI pattern on a 12-lead ECG.
**Room Scenario:**	Participants enter room and the following phrase is either printed and taped on the wall or written on a whiteboard (Appendix B): “If R is far from P, then it must be… If PR gets longer then QRS drops, then it must be … If PR stays normal and QRS quits, then it must be … If P and QRS beat independently, then it must be …” There will be stacks of papers with different ECGs mixed in. ECGs will be labeled 1–4. Laminated answer choices (first degree, Wenckebach, Mobitz 2, third degree) lie randomly on the floor. Clipboards numbered 1–4 will be lined up on a desk. Participants will have to clip the corresponding ECG to the number on the clipboard and then attach which type of block matches the ECG. The answer choices have a specified letter bolded that when placed in the correct order on the clipboards (Wenkeba**C**h, M**O**bitz II, **A** first degree, A **T**hird degree) spells out the word COAT. This is the clue to find the next ECG folded in the pocket of a white coat hanging in the room. On the new ECG, there will be a lateral STEMI. Participants will have to identify the STE in leads 1, avL, 5, 6. This (1VL56) will unlock a box with your directions to the next room. Within the box, there will be a piece of paper with a crossword clue that asks, “What medication reduces mortality in Acute Coronary Syndrome?”
**Materials:**	Random materials to make piles. (Eg, printouts of journal articles, old Annals issues, EM news, pictures of Amal Mattu, etc.) EKGs with the following findings: first degree, Wenckebach, Mobitz III, and third-degree heart blocks labeled 1–4 on top left corner. (Found in Appendix B) EKG with STE in 1 AVL, 5, 6, (Found in Appendix B) 4 clip boards labeled 1–4 lined up in number order Laminated words: WenkebaCh, MObitz II, A first degree, A Third degree. (Found in Appendix B) White coat Letter/number combo lock set to the combination 1VL56 Lock box Crossword puzzle question (Appendix B)
**Facilitator Instructions:**	**Pre session:** Determine how many groups you have and make that many sets of clues. For example, if you have four groups, print four copies of clues and place into four envelopes. Print and laminate EKG and cards found in Appendix B. Gather random materials to make piles to simulate a “messy desk,” such as journal articles, old Annals journals, pictures of Amal Mattu. **During Session:** Stay seated in the room to ensure that participants stay on course. Start the timer from the time participants enter the room, and stop the timer after they finish their crossword puzzle. If they utilize a hint, add 60 seconds to their time. **Post Session:** Re-scatter images. Replenish hint if used during prior session. Restore box and re-set lock code.
**sGAE Answers:**	The full riddle without blanks is: If R is far from P, then it must be **first degree**. If PR gets longer then QRS drops, then it must be **Wenckebach**. If PR stays normal and QRS quits, then it must be **Mobitz II**. If P and QRS beat independently, then it must be **third degree**” First puzzle: Clip numbered ECG to matching number on the clipboards lined up on the desk. Match the correct type of block to the ECG. Once all ECG are correctly identified, bolded letters on each answer choice spell out COAT. Identify the lab coat in the room to find another ECG in the pocket. Second puzzle: Identify that the ECG in lab coat pocket has lateral STEMI (STE in leads 1, avL, 5, 6). Deduce that a letter and number code made from these leads (1VL56) is the answer to unlock the box in the room. Locate crossword clue in box. Exit crossword: “What medication reduces mortality in Acute Coronary Syndrome?” Answer: ASPIRIN (#5 Across)
**Hint:**	“The letters underlined will help you find what you need to get out in time.”

**Table:** 

*Scenario 2:*	FAST (Focused Assessment with Sonography in Trauma)
***Estimated Time to Completion***	Approximately 15 minutes
**Learning Objectives:**	Categorize appropriate images that make up a FAST exam for a trauma patient. Recall the proper management of a tension pneumothorax.
**Room Scenario:**	Participants enter the room to find pictures of a motor vehicle accident scene and several balloons scattered throughout the room. There is a clinical scenario posted. On a desk, the participant finds various ultrasound images located in a folder. The participant must identify the need for a FAST exam in the clinical scenario and identify the correct FAST images. Each of the correct images that comprise a FAST exam will be labeled with a letter. When the participant appropriately identifies the correct ultrasound images, they will spell out a word (LAMP) that leads you to a lamp. Underneath the lamp, the participant finds a barcode sign laminated card, and on the flip side of the card will be a rhyme. Sitting next to the card is a black light. The participant must illuminate the card to reveal the code to a lock box (code 14325). Inside the lock box will be a 14 g needle and a piece of paper with a crossword clue that asks, “What position must the patient be lying in to perform a FAST exam?” The participant must solve the crossword and pop all the balloons with the 14 g needle to find this clue that leads them to the next room: “You inserted into the midclavicular 2^nd^ intercostal space and heard a rush of air. Your team is now preparing for a chest tube. Proceed to ___.”
**Materials:**	Scene photos (supplemental images PowerPoint). Folder. Lamp. Ultrasound pictures (supplemental images PowerPoint). Clinical Case (supplemental documents). Rhyme on a laminated card (supplemental documents). Invisible ink and black light. 14 g 3.25” needle. Opaque balloons. Balloon clue inserts (Appendix B). Box with numerical lock set to 14235. Crossword puzzle question (Appendix B).
**Facilitator Instructions:**	**Pre-Session:** Ensure there is a desk or floor lamp in the room being used. Print motor vehicle accident images, ultrasound images, and “barcode sign image” from Appendix B. Print clinical scenario from Appendix B. Print clues from Appendix B and fold/roll them into a small piece and place in balloons, blow up balloons, and tie. Determine how many groups you have and blow up that many sets of balloons. For example, if you have four groups, print four copies of clues and place into four different sets of balloons. **During Session:** Stay seated in room to ensure that participants stay on course. Start the timer from the time participants enter the room and stop the timer after they finish their crossword puzzle. Ensure participants do not pop balloons before unlocking the lock box. If they utilize a hint, add 60 seconds to their time. **Post Session:** Re-scatter images. Replenish hint if used during prior session. Restore box and re-set lock code. Replenish balloons.
**sGAE Answers:**	First puzzle: Identify that the pictures corresponding to letters L (subxiphoid), A (hepatorenal), M (splenorenal), P (bladder) are the correct ultrasound images needed to make up a FAST exam. Deduce that the letters associated with the image spell out LAMP and they need to look under the lamp in the room. Find the image of the barcode sign under the lamp. Second puzzle: Use blacklight to identify the code on the image (14325) to unlock the box. Use the 14 g angiocath found in the lock box to pop the balloons to find clue to lead to the next room. Exit crossword: “What position must the patient be lying in to perform a FAST exam?” Answer: SUPINE (#4 Down)
**Hint:**	Picture of 14 GA 3.25 IN angiocath (see Appendix B)

**Table:** 

*Scenario 3:*	7 Ps of Intubation
***Estimated Time to Completion***	Approximately 15 minutes
**Learning Objectives:**	Identify an organized approach to emergency department rapid-sequence intubation.
**Room Scenario:**	Participants walk into a room with a large whiteboard. A riddle will be posted: “I am a seed with three letters in my name. Take away the last two and I still sound the same.” This will be in green ink within a thought bubble coming from a picture of a faculty member (choose faculty member that teaches airway or program director). Several pictures will be printed that pertain to the Ps of RSI (LRC item 3) as well as several distractors. These pictures will be scattered throughout the room. On a whiteboard, there will be 7 spaces for them to hang the correct picture corresponding to the correct P in the proper order. When placed in the correct order, the number under the picture slot will indicate the letter to use to spell out the word PEAS. This will be used to open a letter combination lock on a lock box. In the box, there will be airway equipment and the crossword clue, “How many liters per minute typically flows through a nonrebreather?” Participants will have to solve the crossword and then use the airway equipment from the lock box to retrieve the clue from the intubation head’s airway to lead to the next room.
**Materials:**	White board with 7 slots for pictures and corresponding numbers for the clue underneath and the riddle written in word bubble in green. Green dry erase marker. Faculty picture. 7 pictures labeled with the Ps and P distractors (Found in Appendix B). Letter combination lock. Lock box. Intubation head. Direct laryngoscope. McGill forceps. Crossword puzzle question (Appendix B).
**Facilitator Instructions:**	**Pre-Session:** Print all materials from Appendix B and laminate. Delineate space on whiteboard to post pictures and write out the steps. **During Session:** Stay seated in the room to ensure that participants stay on course. Begin the timer from the time participants enter the room and stop after they finish their crossword puzzle. If they utilize a hint, add 60 seconds to their time. **Post Session:** Re-scatter images. Replenish hint if used during prior session. Restore box and reset lock code. Place clue back in mannequin airway.
**sGAE Answers:**	First puzzle: Identify the images that correspond to the correct P of RSI and place in the slot in the correct order (Pre**P**aration, Pr**E**-oxygenation, Preintubation Optimization, P**A**ralysis with Induction, Positioning, Placement with Proof, Po**S**t intubation management). Deduce that the bolded letters spell out PEAS and use that as the letter combination to unlock the box. Second puzzle: Use the airway equipment found in the lock box to attempt to intubate the airway trainer. Identify the clue to the next room in the trainer’s airway. Exit crossword: “How many liters per minute typically flows through a nonrebreather?” Answer: FIFTEEN (#1 Down)
**Hint:**	“Grandma always said PEAS were important to unlock good health!”

**Table:** 

*Scenario 4:*	Pediatrics
***Estimated Time to Completion***	Approximately 30 minutes
**Learning Objectives:**	Recognize acute otitis media. Locate the appropriate antibiotic and pediatric dose to treat acute otitis media via the *Harriet Lane Handbook*. Demonstrate how to apply evidence-based guidelines to a clinical case of neonatal pediatric fever.
**Scenario:**	Participants walk into room and find a photo of bulging/erythematous tympanic membrane next to this riddle prominently displayed: “I have lots to say but never speak, I open but you cannot walk through me. I have a spine but no bones. They call me Ms. Lane.” They should recognize acute otitis media (AOM) by the photo, and then must figure out the correct antibiotic treatment and dosing. They must look up the antibiotic and dosing in the *Harriet Lane Handbook*. They’ll find a *Harriet Lane Handbook* in the room and look up the correct antibiotic and dosing for AOM. On the correct page, there will be a taped key (could also use page number for number lock eg, 00708) to unlock a box with materials for the next puzzle. In the box there will be a baby doll, a toy thermometer reading 103.2° F, a toy doctor bag, 2 pieces of paper in different languages, as well as 2 sheets of cardstock with boxes cut out. Participants will have to find the correct cardstock to use as a codebreaker by overlaying it on the papers written in foreign languages. When they have the correct orientation, they will find that two of the three papers spell out the words “REVISE” and “BLANKET.” The baby doll will have this clinical case taped to the back: “Little Susie is a 24-day-old female born at 39 weeks via uncomplicated spontaneous vaginal delivery presenting for fever. Mother measured a temperature of 103° F at home and brought her right to the PED. Mother denies rash, congestion, vomiting, diarrhea, or sick contacts. No medications were given prior to arrival. On exam she is febrile, tachycardic, tachypneic, and saturating well on room air. You cannot localize a source of fever on exam. What are the 4 next steps in taking care of your patient?” Participants should then deduce they must apply the Revise II criteria to solve the clinical case. They will find a copy of the Revise II guidelines to use for reference wrapped in the blanket. In the room, there will be a play doctor bag with strips of paper management strategies printed on them along with a number. There will also be a riddle that says, “You’re doing just fine! Place these four steps in the correct line. When baby is admitted to the floor, you may proceed to the next door.” Participants must choose the correct management strategy and order based on the Revise II guidelines. The correct order of these numbers [5623] will allow the participants to escape the room. Within the box, there will be a piece of paper with a crossword clue, that asks “At what age in months can babies start taking ibuprofen?”
**Materials:**	Picture of bulging/erythematous tympanic membrane (Appendix B). Room prompt and clue (Appendix B). *Harriet Lane Handbook.* Key Lock. Lock box. Baby doll with case taped to back (Appendix B). Toy thermometer. Two sheets of paper in foreign language with codebreaker sheets (Appendix B). Baby blanket. Printout of Revise II pediatric fever guidelines (Appendix B). Toy doctor bag. Riddle and patient management steps (Appendix B). Crossword puzzle question (Appendix B).
**Facilitator Instructions:**	**Pre-session**: Print all materials from Appendix B. Lay tympanic membrane picture, room prompt, clue, and *Harriet Lane Handbook* on desk. Tape key to lock box on the correct page for the pediatric dosing of amoxicillin for acute otitis media. Lock the following items in the lockbox: baby doll with case taped to back, foreign language papers, codebreaker sheets, printout of Revise II pediatric fever guidelines wrapped in baby blanket, toy doctor bag with riddle and patient management steps inside. **During Session**: Stay seated in the room to ensure that participants stay on course. Time from the time participants enter the room and stop after they finish their crossword puzzle. If they utilize a hint, add 60 seconds to their time. Verify the correct order of patient management steps to participants. Post Session: Re-organize tympanic membrane picture, room prompt, clue, and *Harriet Lane Handbook* on desk. Ensure key to lock box is taped on the correct page for the pediatric dosing of amoxicillin for acute otitis media. Ensure lock box contains correct materials: baby doll with case taped to back, foreign language papers, codebreaker sheets, printout of Revise II pediatric fever guidelines wrapped in baby blanket, toy doctor bag with riddle and patient management steps inside. Ensure box is locked. Replenish hint if used during session.
**sGAE Answers:**	First puzzle: Identify AOM and look up the correct antibiotic dosing in the *Harriet Lane Handbook.* Locate the correct dosing information in the *Harriet Lane Handbook* and find a key taped to that page. Use key to open lock box. Second puzzle: Identify the clues REVISE and BLANKET by using the code breaker sheets over the foreign language papers. Locate the blanket in the room to find the Revise II criteria. Locate the play doctor bag to find paper strips for management of the clinical case taped to the back of the baby. Management steps are placed in the correct order: 5623. This allows the facilitator to let them escape the room once the crossword puzzle is completed. Exit crossword: “At what age in months can babies start taking ibuprofen?” Answer: SIX (#3 Across)
**Hint:**	“Through Ms. Lane, you’ll find the antibiotic dosing on the correct page. That’ll let the next clues out of their cage!”

**Table:** 

*Scenario 5:*	Neurology
***Estimated Time to Completion***	Approximately 15 minutes
**Learning Objectives:**	Recall common clinical findings of basilar skull fracture. Identify important concepts in the management of stroke syndromes.
**Scenario**	Participant walks into a room and finds a racoon image with toy “googly eyes” on it. Next to the raccoon is a toy skull on top of a *Tintinalli’s Emergency Medicine* textbook. Participants must deduce that “raccoon eyes” are indicative of a basilar skull fracture. The temporal bone of the skull will have invisible ink written on it that has a code (ours was the number extension to call CT techs). The code will lead to a lock box and inside the participant will find an actual patient picture of racoon eyes and a picture that is cut up in pieces. They must put the pieces together and tape them up to create the multiple-choice quiz. The correct answers of the quiz will spell out a person’s name and that will be the office that will be the next room. Within the box, there will be a piece of paper with a crossword clue that asks, “What push dose medication is typically used to lower blood pressure in acute stroke?”
**Materials:**	Racoon and Raccoon eyes image (Appendix B). Googly eyes. Toy anatomic skull. *Tintinalli’s Emergency Medicine* textbook. Invisible ink and black light. Lock box and lock. Quiz (Appendix B). Crossword puzzle question (Appendix B).
**Facilitator Instructions**	**Pre-session:** Print out enough quizzes so that there is one for each group. Ensure that the multiple-choice answers of the quiz spell out the name of the office where your group should go next. Print raccoon eyes photos from appendix B and attach googly eyes to racoon. Write out code on toy skull in invisible ink (we wrote a code that was the phone number extension to call our CT techs). **During Session:** Stay seated in room to ensure that participants stay on course. Time from the time participants enter room and stop after they finish their crossword puzzle. If they utilize a hint, add 60 seconds to their time. **Post Session:** Replenish hint if used during prior session. Restore box and re-set lock code. Refresh quiz.
**sGAE Answers:**	First puzzle: Interpret that the raccoon picture with googly eyes means basilar skull fracture. Open skull and find code written in invisible ink with black light on temporal bone (this code can be customized; we recommend the extension number to call CT). Use this code to open lock box. Second puzzle: Assemble multiple choice quiz from pieces in lockbox. Appropriately answer quiz (quiz and answers found in Appendix B) and then deduce that the letters corresponding to the correct answers spell out the location of the next room. Exit crossword: “What push dose medication is typically used to lower blood pressure in acute stroke?” Answer: LABETALOL (#2 Down)
**Hint:**	“Your patient has increasing intracranial pressure! You might want to pop the top to shed light on what to do next.”

**Table:** 

*Scenario 6:*	Sepsis
***Estimated Time to Completion***	**Approximately 30 minutes**
**Learning Objectives**	Recognize vital sign abnormalities that could indicate sepsis. Review important concepts related to the management of septic patients.
**Scenario**	Participants enter the room and see a periodic table on the wall. There is a prompt written that says, “This patient looks sick. What vital information do you need next?” The prompt should be printed on a piece of paper that is very clearly and obviously visible, so it immediately catches the participants attention. Next to the prompt should be a picture of an elderly patient. The prompt should lead the participant to search for more information. There will be a manila envelope or folder on the table with EMS report and sheet from a long-term care facility (Appendix B) enclosed. Participants will have to deduce that the bolded letters in Mike **Sr. Ca**n’t **Th**ink Straight” in enclosed paperwork corresponds to atomic numbers in the periodic table (Sr = strontium = 38 for temp; Ca = calcium = 20 for RR; Th = thorium = 90 for HR). Participants will plug those numbers into the vital sign sheet that is enclosed within the envelope/folder to get a code that unlocks a box. Once the box is unlocked, participants will find the exit crossword clue that asks, “How many blood cultures are drawn in a sepsis workup?” Additionally within the box, there will be a saline bag, blood cultures, saline flushes, and a sheet that says, “Nice catch, Doctor! You identified a septic patient, and you are ready to treat them. You draw blood cultures, start IV fluids, and begin initiating broad spectrum antibiotics! The nurse comes up to you and asks, “Doctor, which antibiotics do you want to use? It has been almost three hours!” This will lead participants to a timer set to 3:00 hours. Underneath the timer, participants will find another prompt written on a small sheet of paper (can be folded or cut into a small size to fit underneath the timer). Next to the timer, there will be two photos (Appendix B), and participants will have to deduce that these represent hospital and community acquired infections. Each syringe from the lock box will have a certain number of milliliters and labels for different drugs. The participants must identify the correct antibiotic syringes (determined by the facilitator before start of activity) and use the milliliters in each to identify a code. This code must be given to the facilitator to allow them to escape the room after the exit crossword is completed.
**Materials:**	Periodic Table with atomic numbers; consider a table without atomic weights or cover atomic weight numbers. Folder. Long-term care facility vital sign sheet (Appendix B). Patient photo (Appendix B). Lock box with 5- or 6-digit combination lock, preferably 6 digits. Prompt within lock box (Appendix B). Saline bag. Blood culture bottles x 2. 10 cc saline syringes (Push portion out of 10 cc syringe to the correct number, some will have 5 ccs, 6 ccs, 8 ccs, etc. to make the correct code to get you to the next room). In addition to pushing out the correct amount of fluid from each syringe, circle the number (5 ccs, 6 ccs, 8 ccs, etc.) on the syringe with a permanent marker. ○ Zosyn ○ Vancomycin ○ Ceftriaxone ○ Ancef ○ Gentamycin ○ Solumedrol ○ Morphine ○ Toradol Water to refill the syringes if needed. Clock or timer set to 3 hours. Timer Prompt (Appendix B). Antibiotic prompt pictures (Appendix B). Community and hospital pictures (Appendix B).
**Facilitator Instructions:**	**Pre-session:** Print out prompts. Set time to 3:00. Place materials in box. Put the number of CCs in the syringes with the correct antibiotics that correlate to room number where participants should go next. **During Session:** Stay seated in room to ensure that participants stay on course. Time from the time participants enter room and stop after they finish their crossword puzzle. If they utilize a hint, add 60 seconds to their time. **Post Session:** Replenish hint if used during prior session. Restore box and re-set lock code. Check syringes to ensure the correct amount of fluid is present; replenish with water if needed.
**sGAE Answers:**	First puzzle: Find file with patient information that says “Mike **Sr. Ca**n’t **Th**ink Straight.” Deduce that the underlined components correspond to atomic numbers in the periodic table (Sr = strontium = 38 for temp; Ca = calcium = 20 for RR; Th = thorium = 90 for HR). Use these numbers in the order that appear on the patient information sheet to unlock the box (382090; 0 will need to be dropped if lock box only allows 5 numbers). Second puzzle: Deduce from the prompt in the box that the 3-hour timer must be found. Locate prompt under timer. Deduce that photos represent community and hospital acquired infections. Correctly identify the appropriate antibiotic syringes that typically treat community (Ceftriaxone) and hospital acquired infections (Vancomycin and Cefepime). Report to the facilitator the number of milliliters in each of the correct antibiotic syringes to make the code to exit room once crossword is completed. Exit crossword: “How many blood cultures are drawn in a sepsis workup?” Answer: TWO (#6 Across)
**Hint:**	“Placed behind other numbers, it makes them greater. Next to one, it becomes ten. To unlock the box, take away the number or you will be here later!”* *This hint should be included if lock box is a 5 letter combination; do not use if lock box is a 6 letter combination.
Hint:	“I build the world, yet I am unseen, In every breath and in machines. I form the codes in DNA, And light the stars that guide your way. To crack the code, you must enable Match what’s underlined to the table.”

**Figure:**
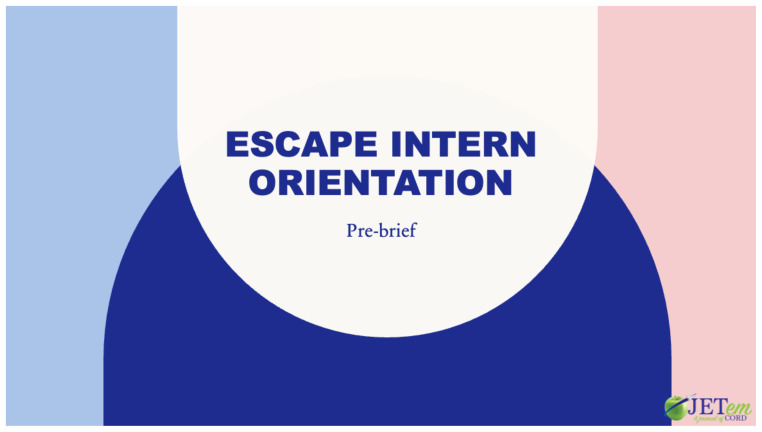
Please see associated PowerPoint file

**Figure:**
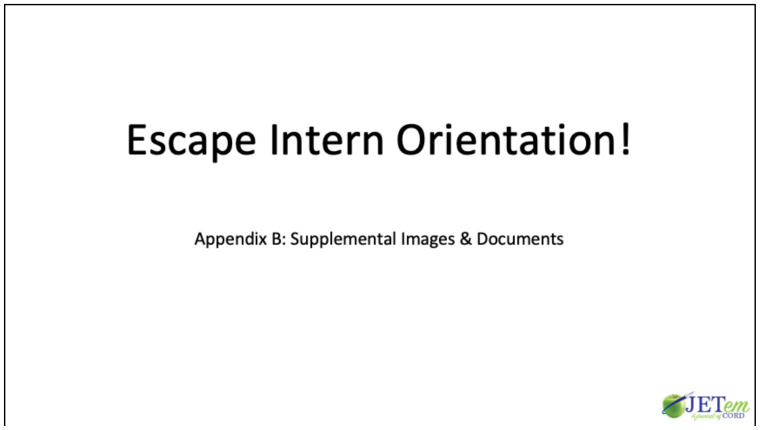
Please see associated PowerPoint file

**Figure:**
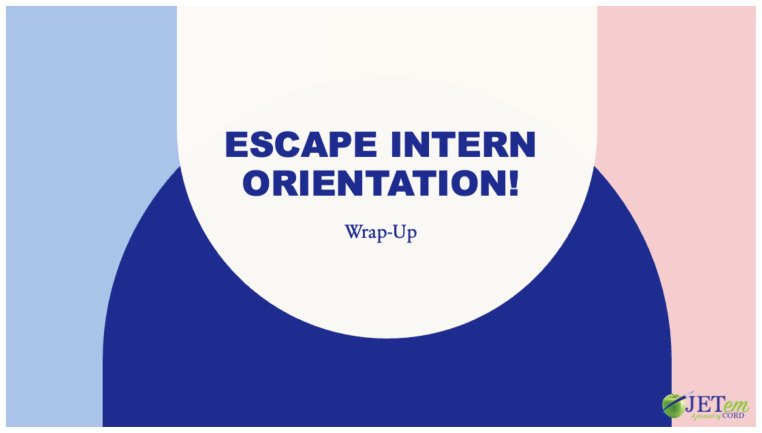
Please see associated PowerPoint file

## SMALL GROUPS LEARNING MATERIALS

### Escape Room Scenarios

**Brief Wrap Up**: See Appendix E

### Pearls

The information below is included via a QR code for participants to review in their own time after the Brief Wrap-up Presentation (see Appendix E).

Station 1: ECG

★ Rhyme to remember heart blocks:
  ○ If R is far from P, then it must be First Degree
  ○ If PR gets longer then QRS drops, then it must be Wenckebach
  ○ If PR stays normal and QRS quits, then it must be Mobitz II
  ○ If P and QRS beat independently, then it must be Third Degree

Station 2: FAST

★ The traditional FAST (Focused Assessment with Sonography in Trauma) exam has 4 views: subxiphoid, hepatorenal, splenorenal, and bladder.
★ Add lung views on right and left to make it an EFAST (extended FAST) exam.
★ Use M-mode to help establish the diagnosis of a pneumothorax. It will look like a barcode as opposed to a sandy beach in appearance.
★ The FAST exam should be performed with the patient SUPINE for the most accurate results.
★ If the patient is unstable with a tension pneumothorax, use a 14-gauge angiocath to perform a needle decompression.

Station 3: 7 Ps

★ Ensure you remember the 7 Ps to achieve a successful intubation
  ○ Preparation (most important!)
  ○ Pre-oxygenation
  ○ Pre-intubation optimization
  ○ Paralysis with induction
  ○ Positioning
  ○ Placement with proof
  ○ Post-intubation management (have a plan before the procedure!)
★ A brief airway assessment is key to adequately prepare for intubation.

Station 4: Peds

★ The *Harriet Lane Handbook* is your go-to resource in the pediatric ED, especially antibiotic dosing.
★ Neonatal pediatric fever is managed by evidenced based guidelines. Be familiar on how to access these and any institution specific modifications.

Station 5: Neurology

★ Signs of basilar skull fracture include raccoon eyes, Battle’s sign (bruising behind ear), cerebrospinal fluid (CSF) otorrhea or rhinorrhea.
★ Become familiar with the Glasgow Coma Scale (GCS)–normal is 15 points and you get 3 of those for just showing up.
★ The National Institute of Health Stroke Scale is used to document the severity of stroke, with the higher the number correlating with increased severity.
★ Hypoglycemia is a common stroke mimic. As such, a fingerstick glucose should be obtained immediately when a patient exhibits signs of stroke.
★ A non-contrast head CT is required prior to administering thrombolytics for stroke.
★ Thrombolytics must be given within 3–4.5 hours of a patient’s last known normal (presumed time of symptom onset).
★ Become familiar with the many contraindications to thrombolytics.

Station 6: Sepsis

★ SIRS (systemic inflammatory response syndrome) criteria components are heart rate, respiratory rate, white blood cell count, and temperature.
★ Don’t forget that leukopenia and hypothermia are included in SIRS criteria components.
★ Blood pressure and lactate level are not components of SIRS criteria.
★ **Almost all septic patients have SIRS but not all patients with SIRS have sepsis.**
★ Become familiar with empiric antibiotic choices for community and hospital acquired infections.
★ Hospital acquired infection coverage is typically aimed at empirically covering for MRSA (methicillin resistant staphylococcus aureus) and pseudomonas.

## Appendix C Materials Master Checklist

□ *Harriet Lane Handbook*
□ *Tintinalli’s Emergency Medicine Textbook*
□ 14g 3.25” needle
□ Blood Culture Bottles x 2
□ Liter Bag of Normal Saline
□ 10 cc Syringes filled with water/saline x 8–10
□ Water to refill syringes
□ McGill Forceps
□ Direct Laryngoscope/Simulator
□ Intubation Head Mannequin
□ White Coat
□ Baby Blanket
□ Toy Baby doll
□ Toy Anatomic Skull
□ Toy Doctor Bag
□ Toy thermometer
□ Balloons, opaque x 100
□ Lamp
□ Lock Box with numerical code
□ Clock or Timer
□ Folders x 2
□ Clip Boards x 4
□ Lock Box x 5
□ Combined letter/number combination lock x 1
  ○ Code: 1VL56 (Scenario 1/EKG)
□ Numerical combination lock x 3
  ○ Code: 14235 (Scenario 2/FAST)
  ○ Code: Customized (Scenario 5/Neurology)
  ○ Code: 38209[0] (Scenario 6/Sepsis)
□ Lock with traditional key x 1
  ○ Scenario 4/Pediatrics
□ Letter combination lock
  ○ Code: PEAS (Scenario 3/7 P’s of Intubation)
□ Craft “googly” eyes
□ Invisible Ink Marker with Black Light x 2
□ Green Dry Erase Marker
□ Laminator
□ Large Periodic Table
□ Random Materials to make piles
  ○ Examples: Journal article printouts, old *Annals* issues, EM news
□ Whiteboard
□ Picture of Faculty Member Who Teaches Airway
□ Printed Supplemental Images/Documents/Hints from Appendix B

## Appendix D Participant Sheet

### INSTRUCTIONS

1. **Print out the sheet below and place on clipboard; provide one sheet and clip board to each group.**
2. **Adjust end point for group to meet your specific arena.**
3. **Can use online crossword puzzle generators to create new crossword puzzles to meet other objectives.**

### ESCAPE INTERN ORIENTATION!

**Group Name:**

**Table:**

TIME	
Room 1	
Room 2	
Room 3	
Room 4	
Room 5	
Room 6	

Proceed to Room *** once your puzzle is complete!

*** Add room specific to your building
