# Supplementary material for: Escape Intern Orientation! — A Capstone and Team Building Activity for New EM Interns
Source: J Educ Teach Emerg Med. 2026 Apr 30;11(2):SG1–SG35. doi: 10.5070/M5.52158 (PMC13152383; doi:10.5070/M5.52158)
Supplement: Supplementary file 1 [file 11-2-SG1-Appendix_A.pptx]

## Slide 1
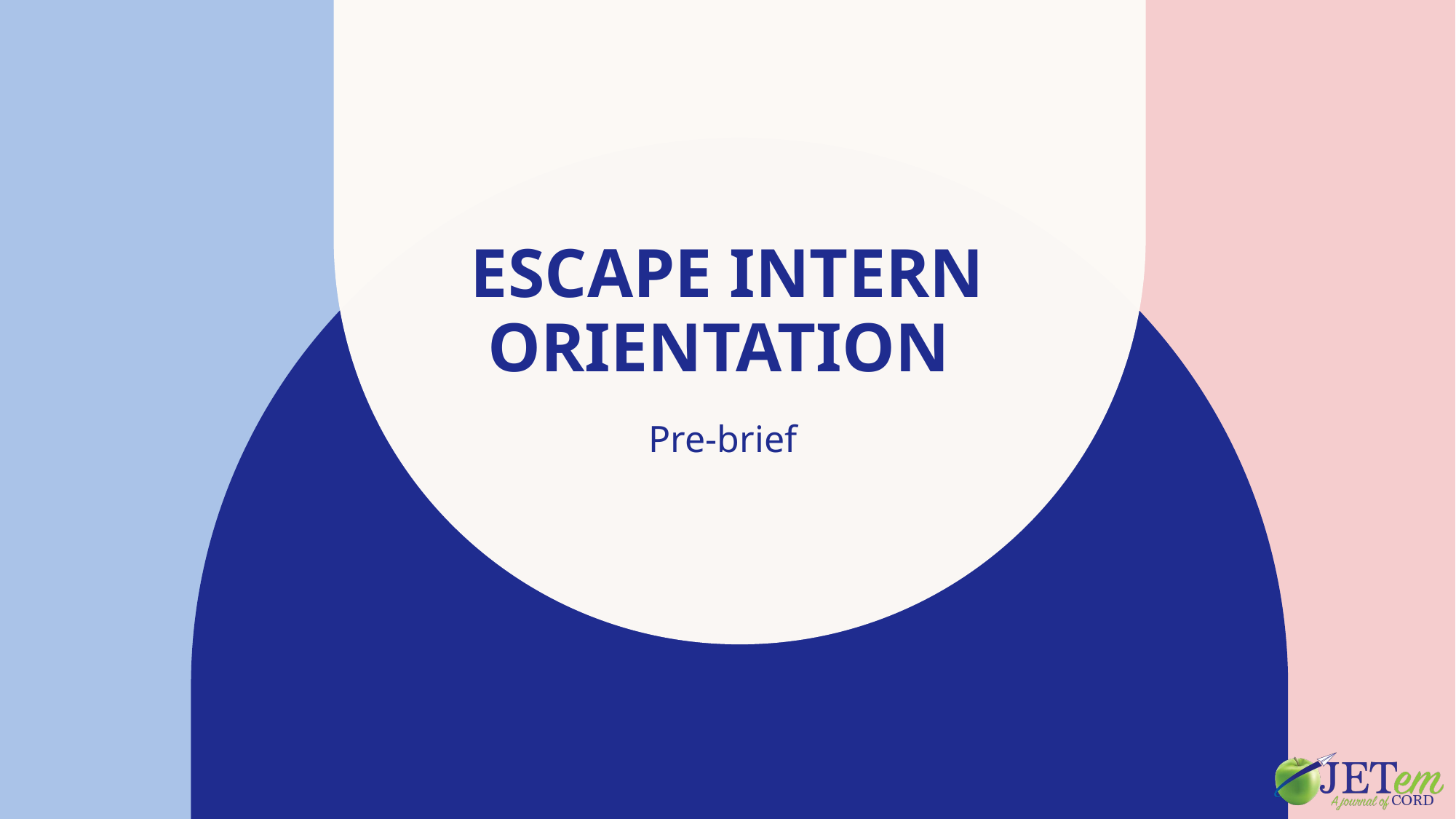

# Escape Intern orientation
Pre-brief

## Slide 2
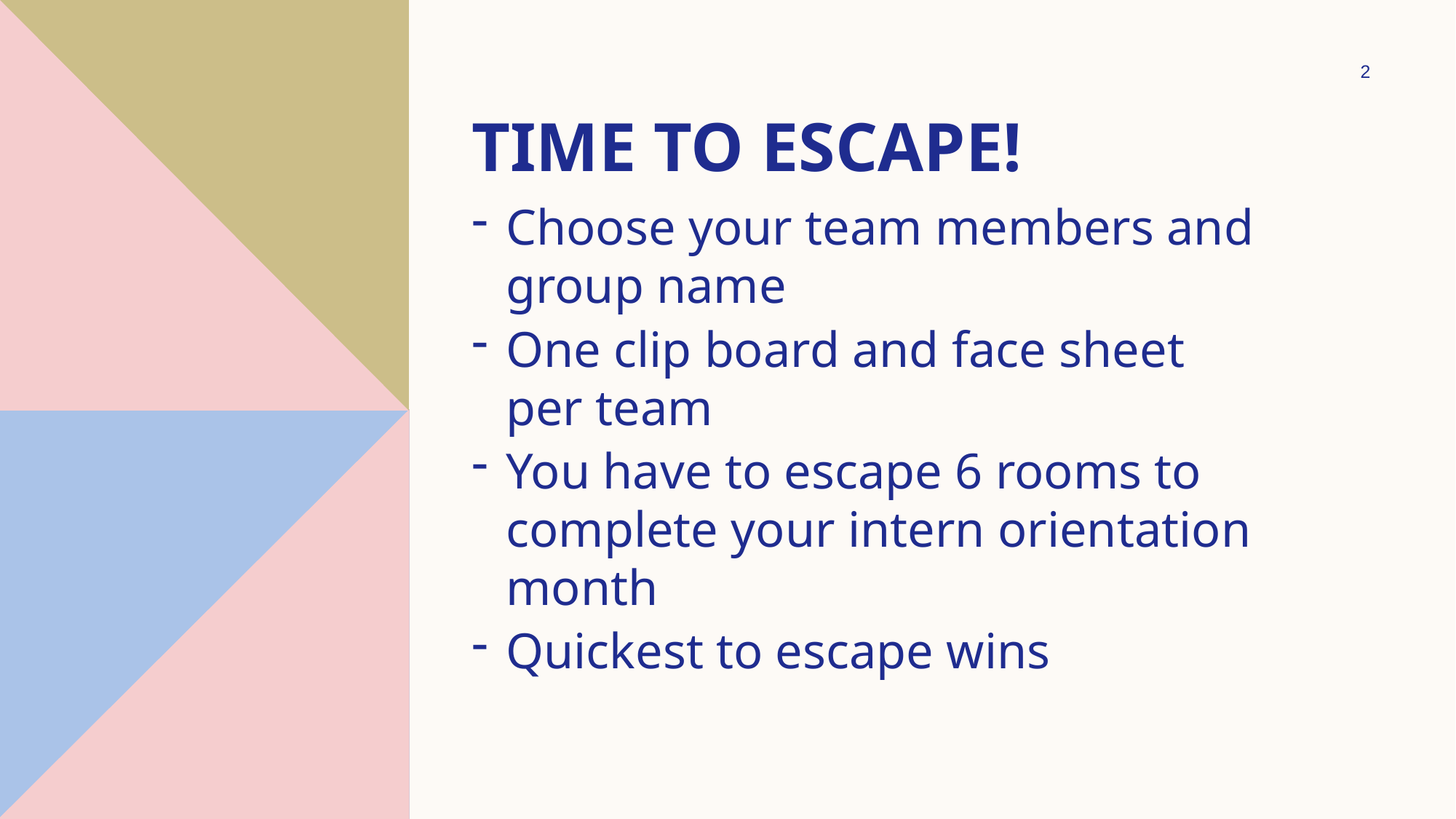

2
# Time to escape!
Choose your team members and group name
One clip board and face sheet per team
You have to escape 6 rooms to complete your intern orientation month
Quickest to escape wins

## Slide 3
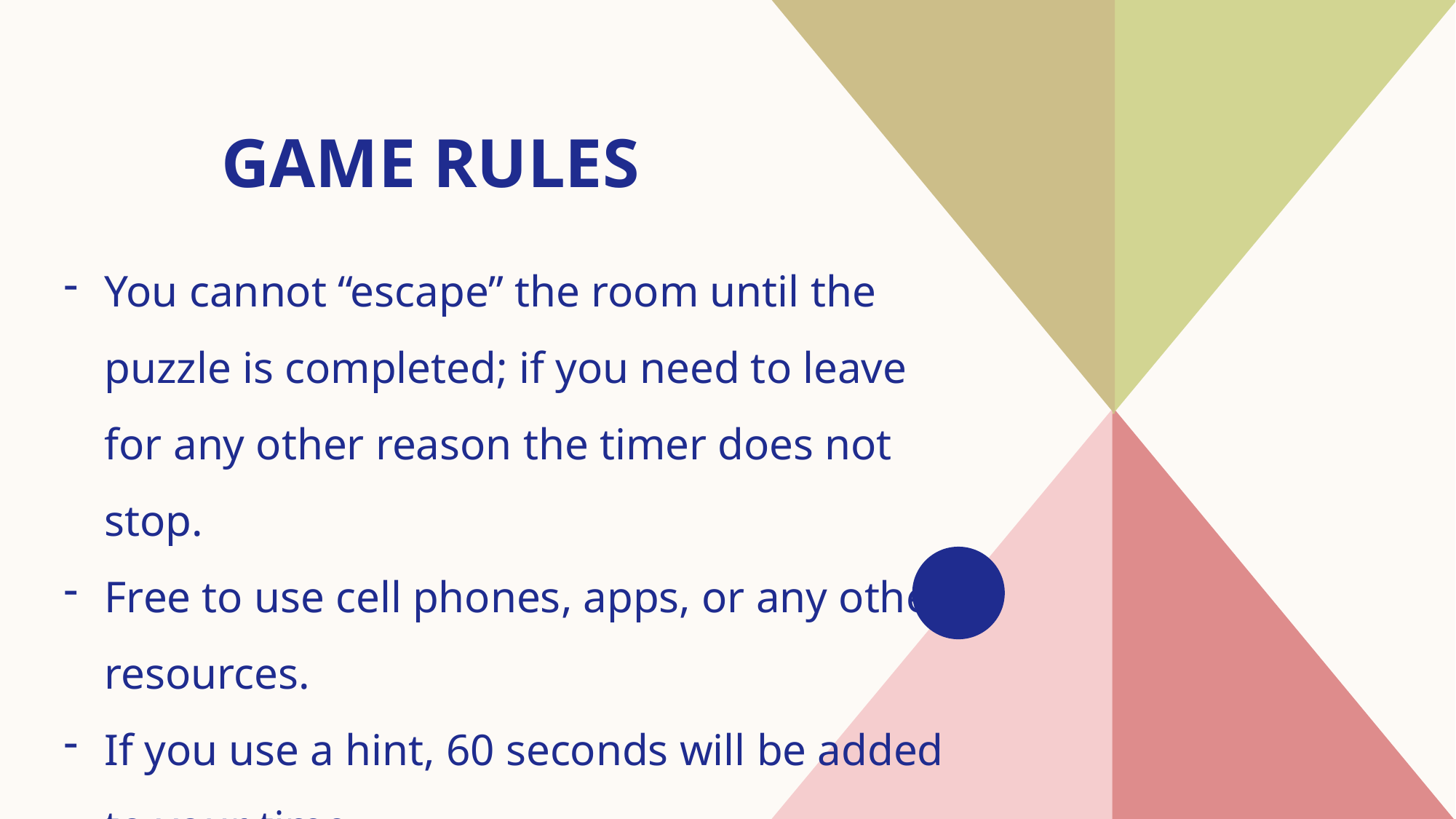

# Game rules
You cannot “escape” the room until the puzzle is completed; if you need to leave for any other reason the timer does not stop.
Free to use cell phones, apps, or any other resources.
If you use a hint, 60 seconds will be added to your time.

## Slide 4
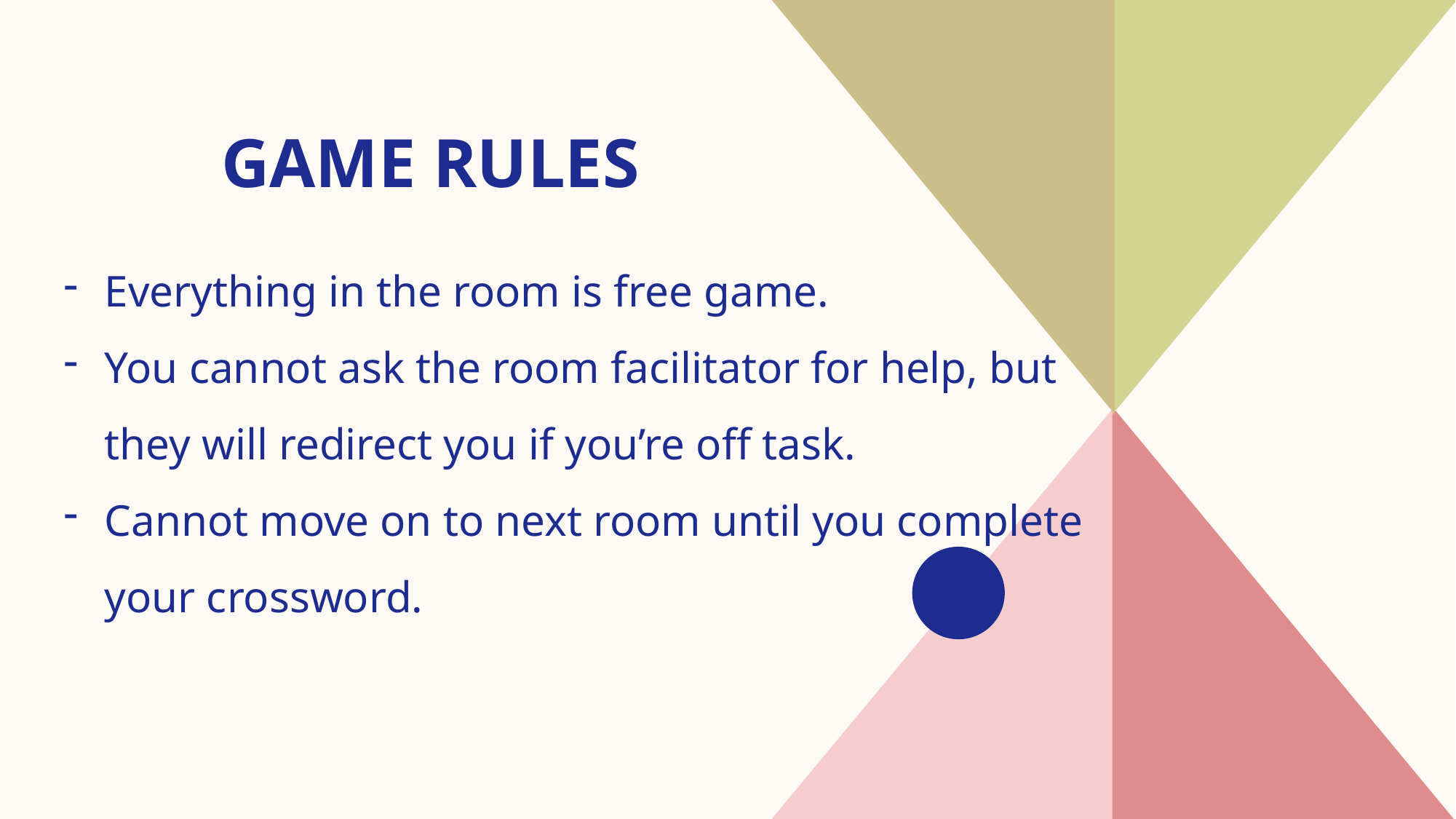

# Game rules
Everything in the room is free game.
You cannot ask the room facilitator for help, but they will redirect you if you’re off task.
Cannot move on to next room until you complete your crossword.

## Slide 5
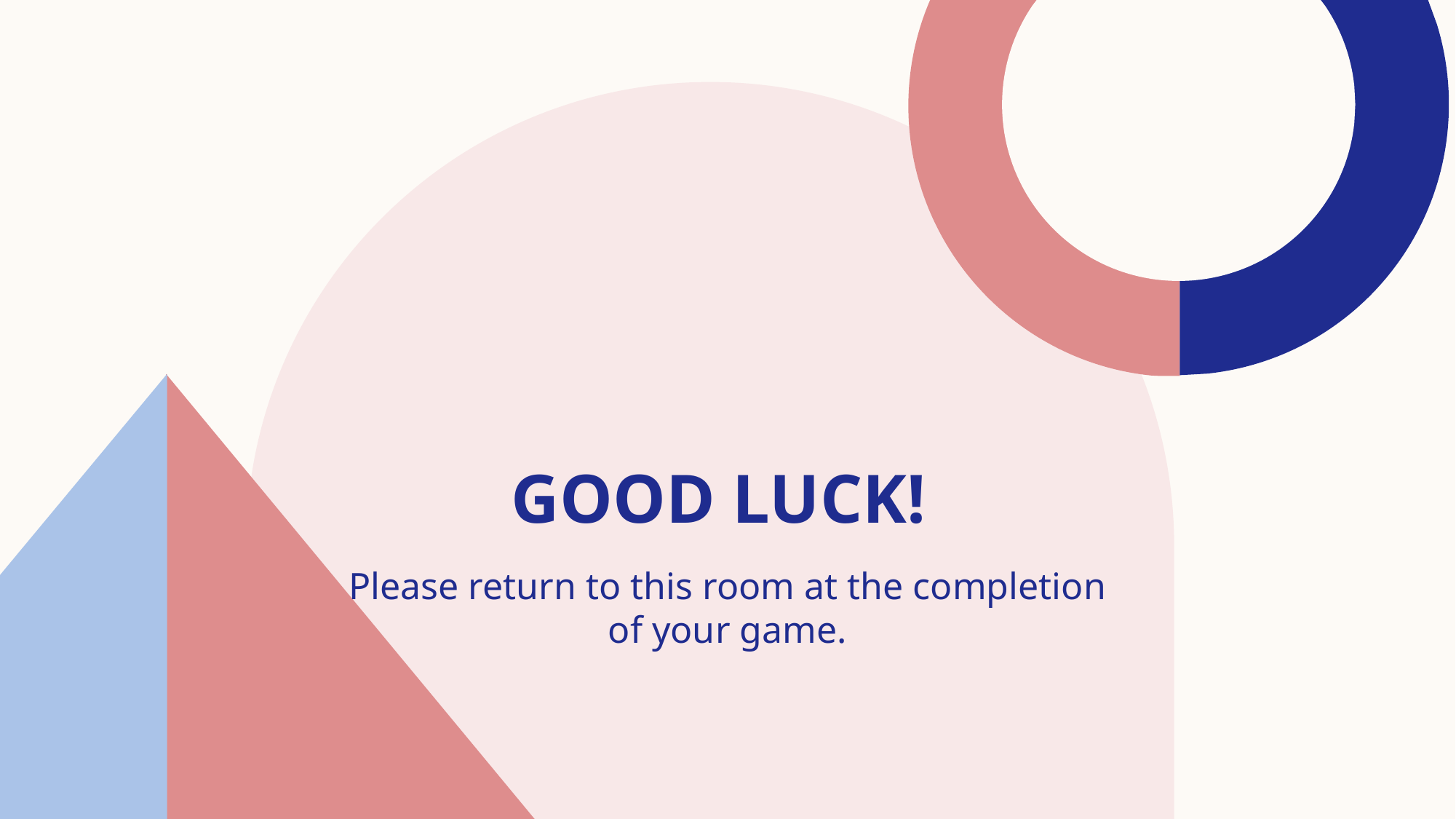

# GOOD LUCK!
Please return to this room at the completion of your game.
