# Supplementary material for: Escape Intern Orientation! — A Capstone and Team Building Activity for New EM Interns
Source: J Educ Teach Emerg Med. 2026 Apr 30;11(2):SG1–SG35. doi: 10.5070/M5.52158 (PMC13152383; doi:10.5070/M5.52158)
Supplement: Supplementary file 3 [file 11-2-SG1-Appendix_E.pptx]

## Slide 1
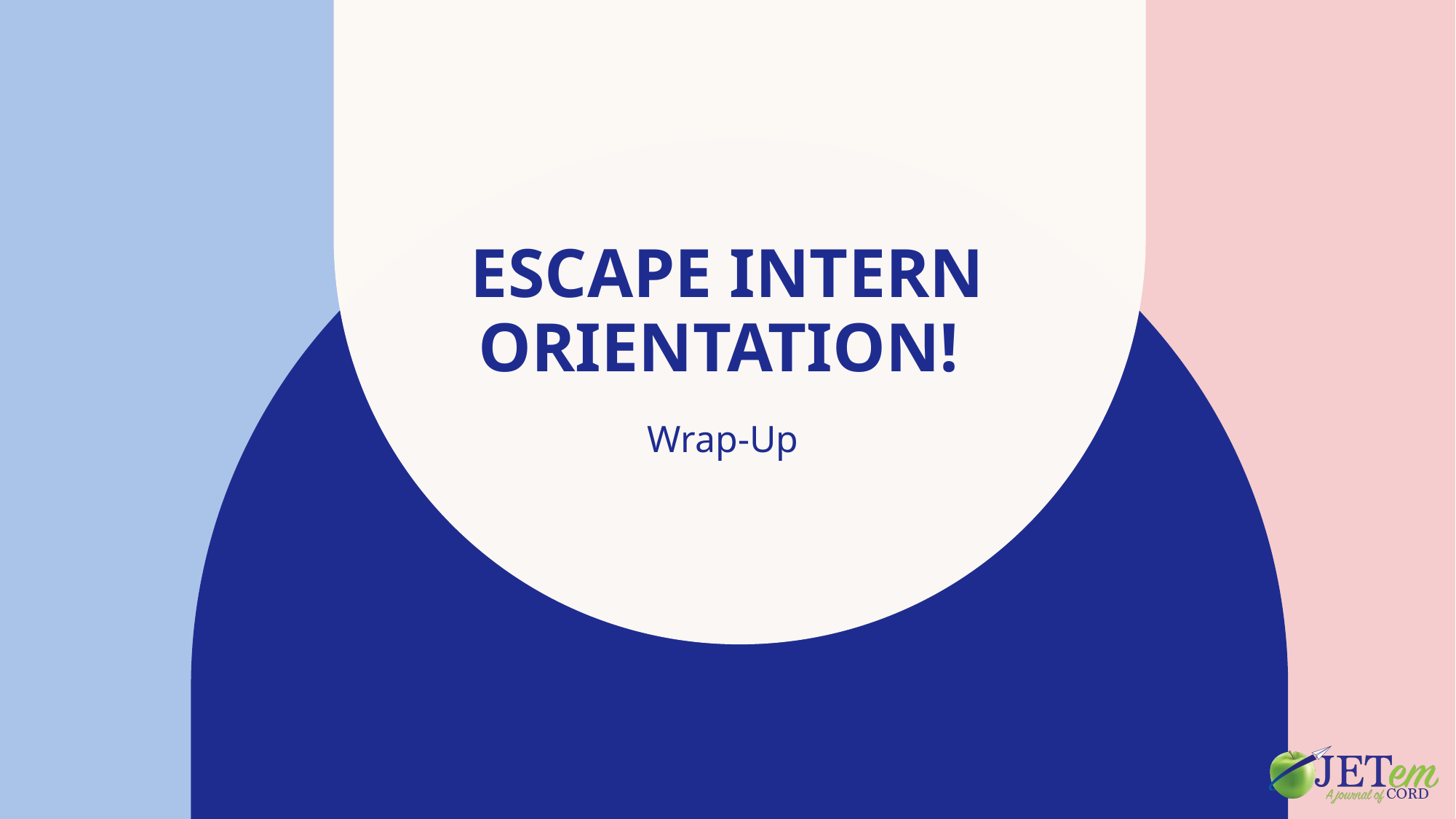

# Escape Intern orientation!
Wrap-Up

## Slide 2
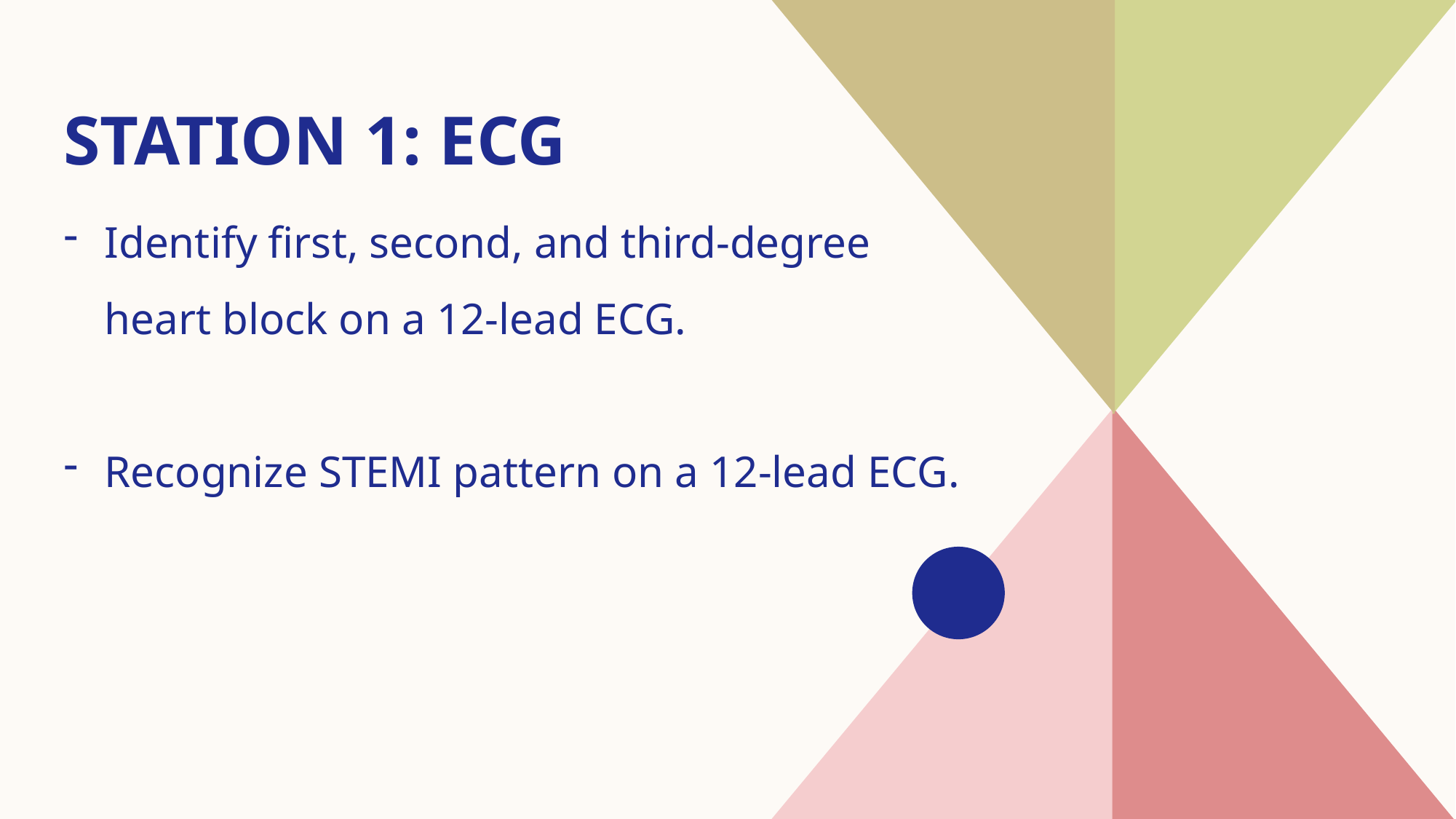

# Station 1: ECG
Identify first, second, and third-degree heart block on a 12-lead ECG.
Recognize STEMI pattern on a 12-lead ECG.

## Slide 3
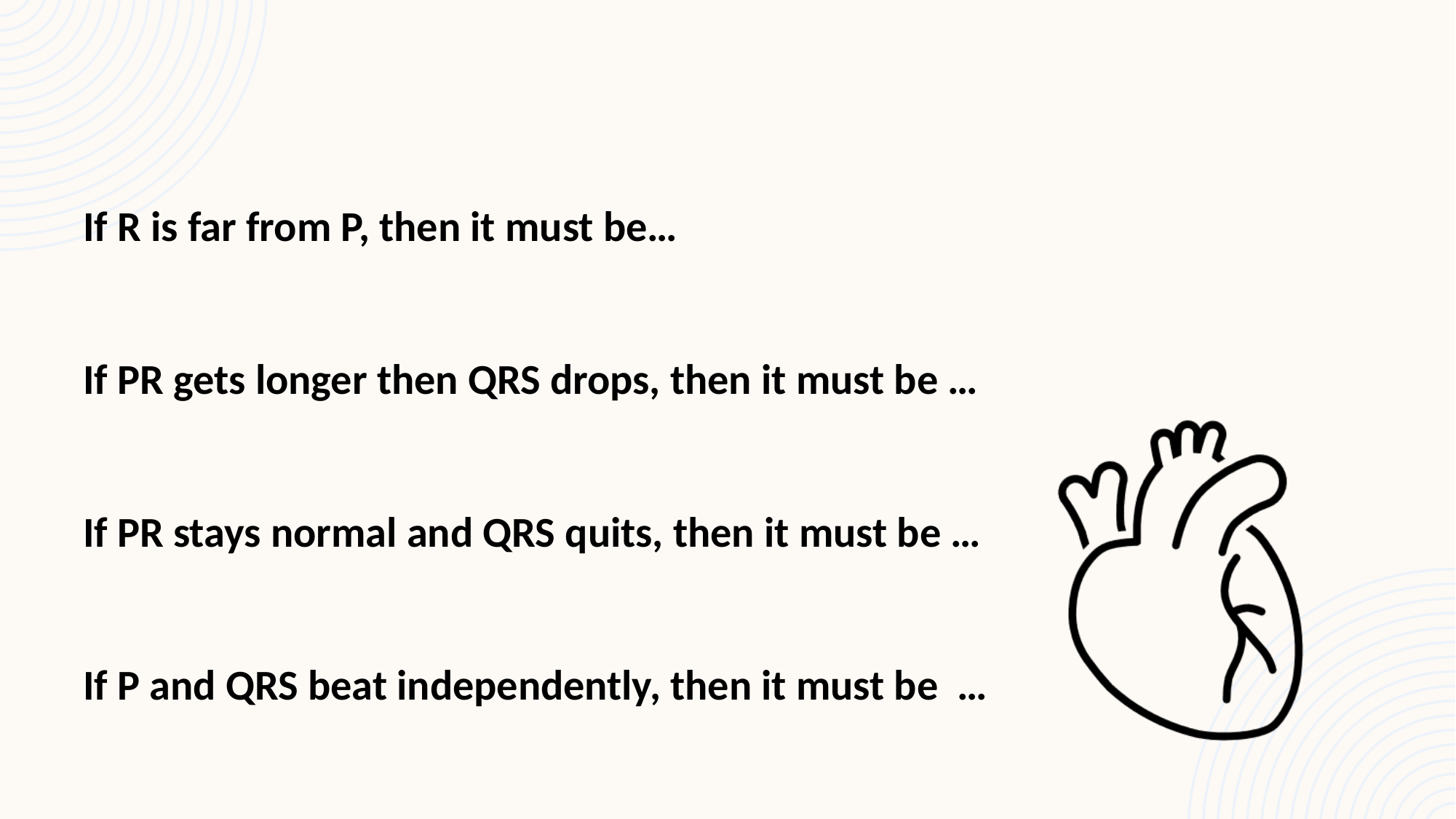

If R is far from P, then it must be…
If PR gets longer then QRS drops, then it must be …
If PR stays normal and QRS quits, then it must be …
If P and QRS beat independently, then it must be …

## Slide 4
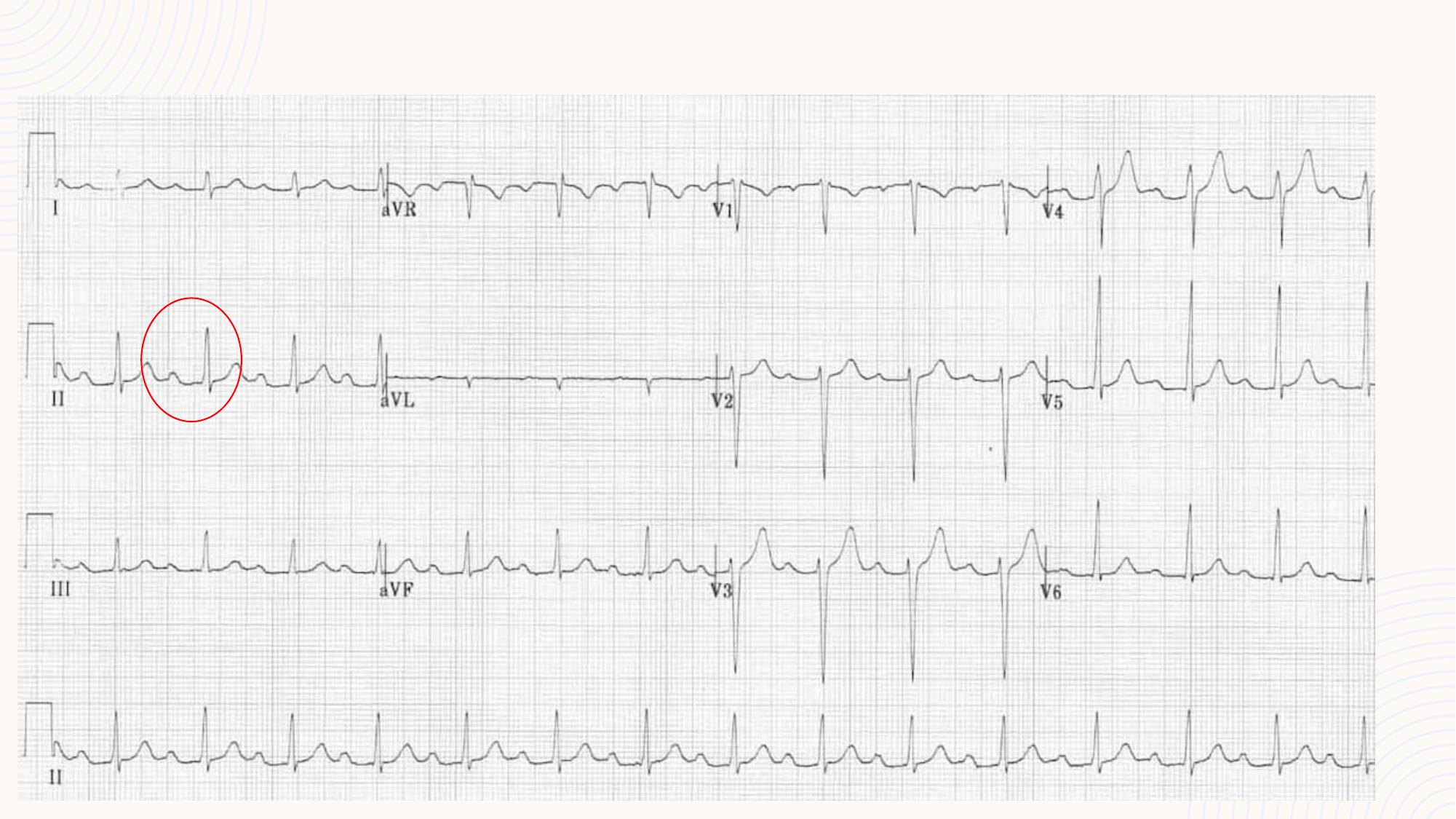

## Slide 5
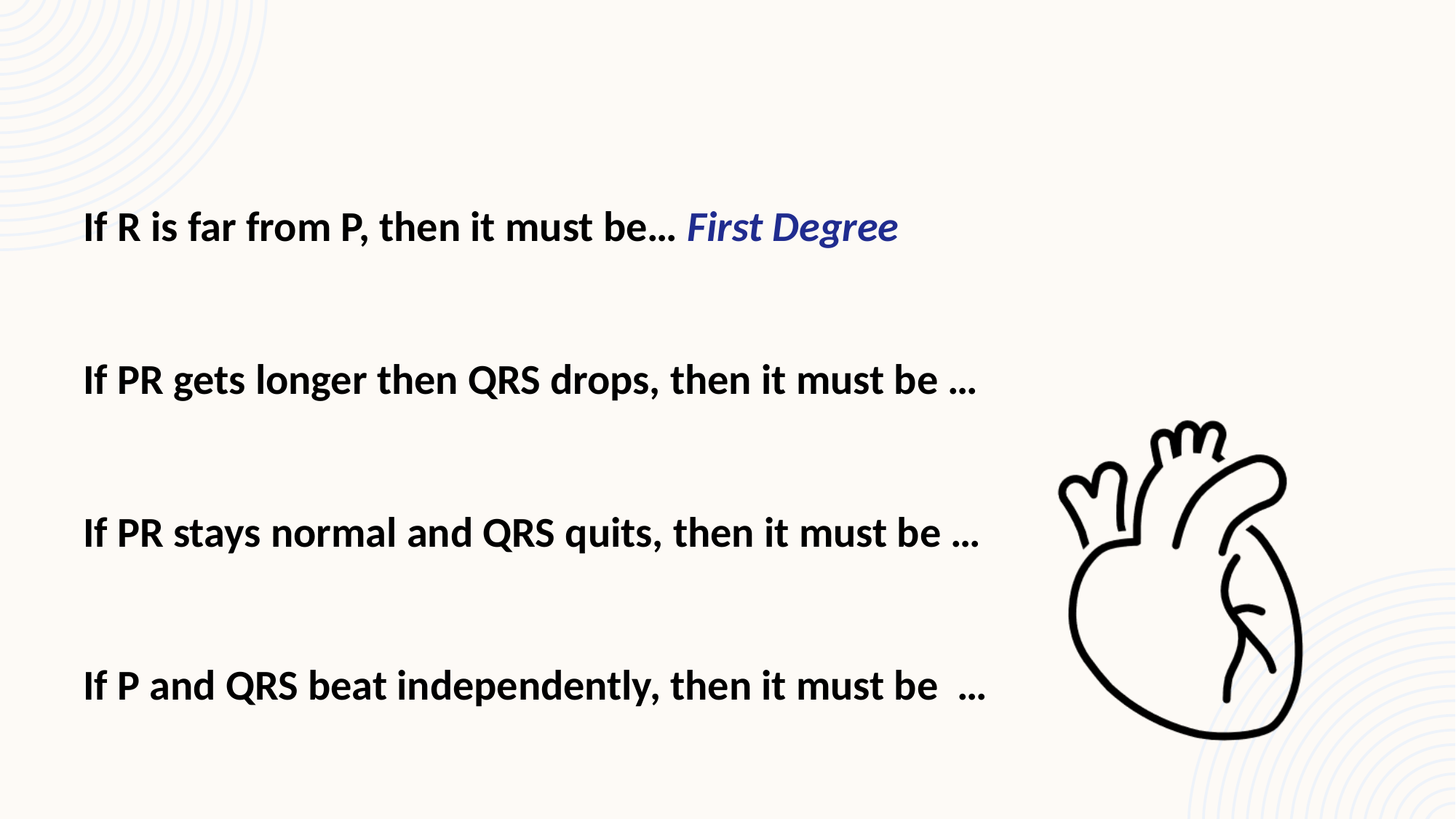

If R is far from P, then it must be… First Degree
If PR gets longer then QRS drops, then it must be …
If PR stays normal and QRS quits, then it must be …
If P and QRS beat independently, then it must be …

## Slide 6
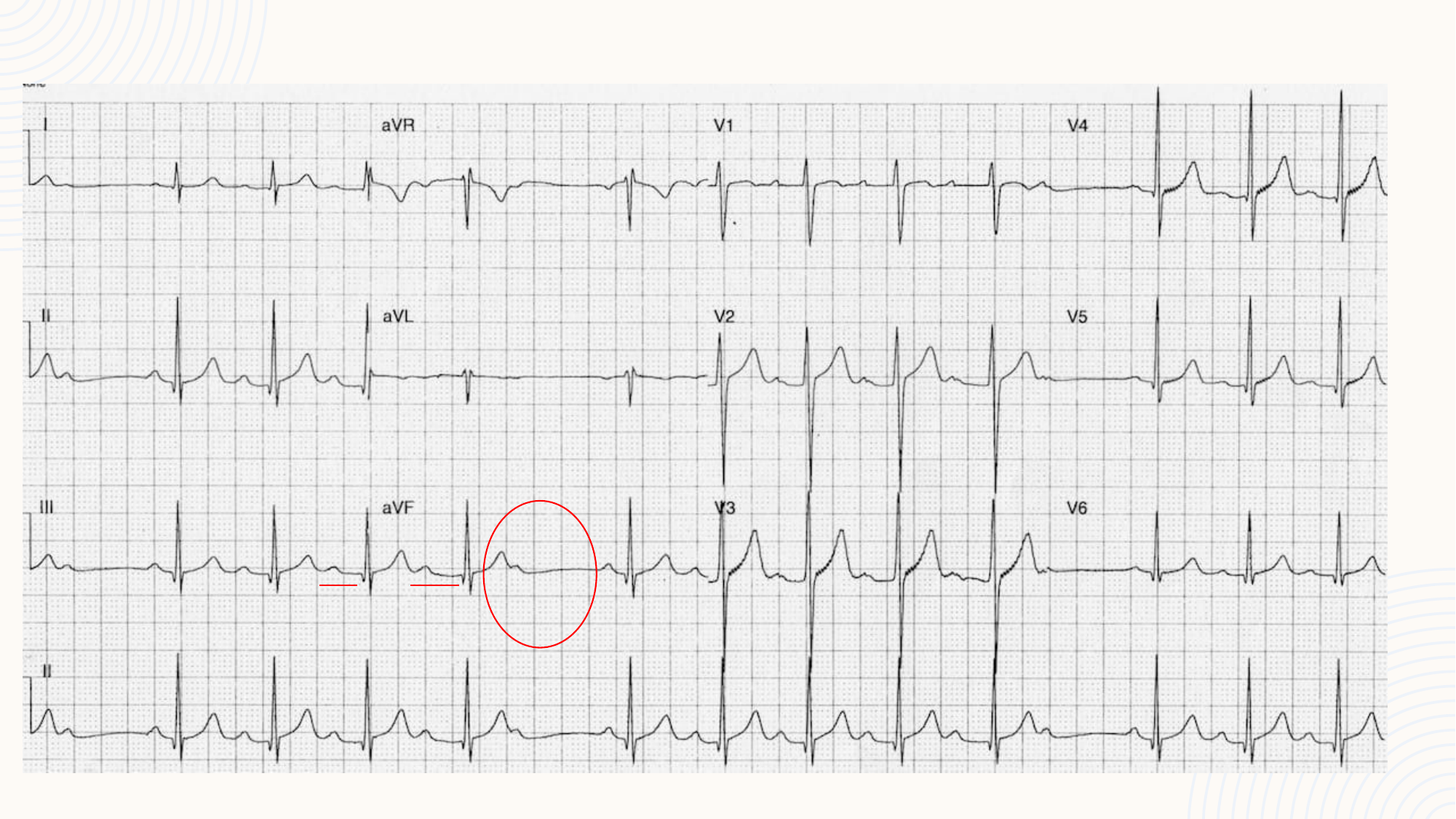

## Slide 7
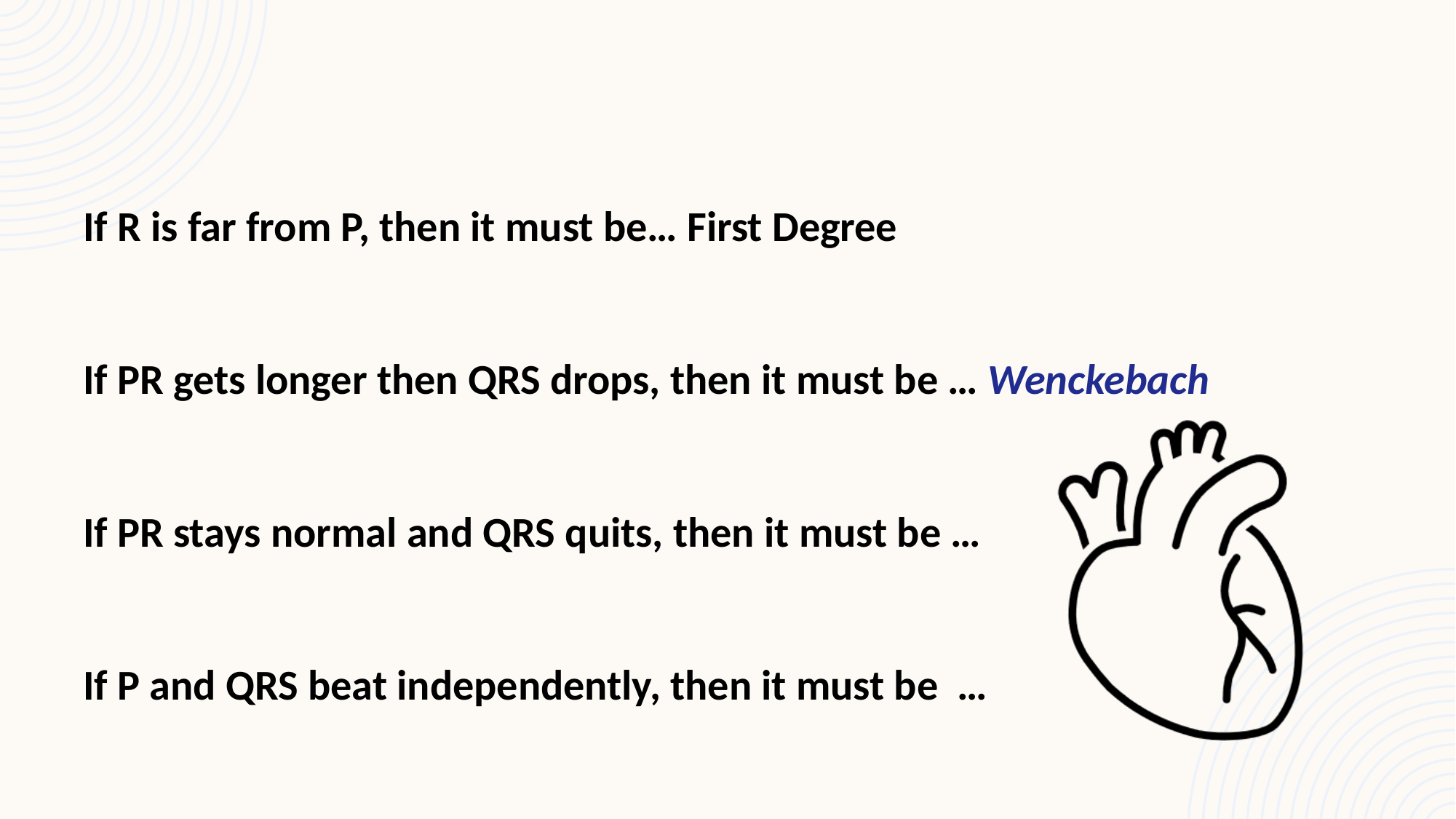

If R is far from P, then it must be… First Degree
If PR gets longer then QRS drops, then it must be … Wenckebach
If PR stays normal and QRS quits, then it must be …
If P and QRS beat independently, then it must be …

## Slide 8
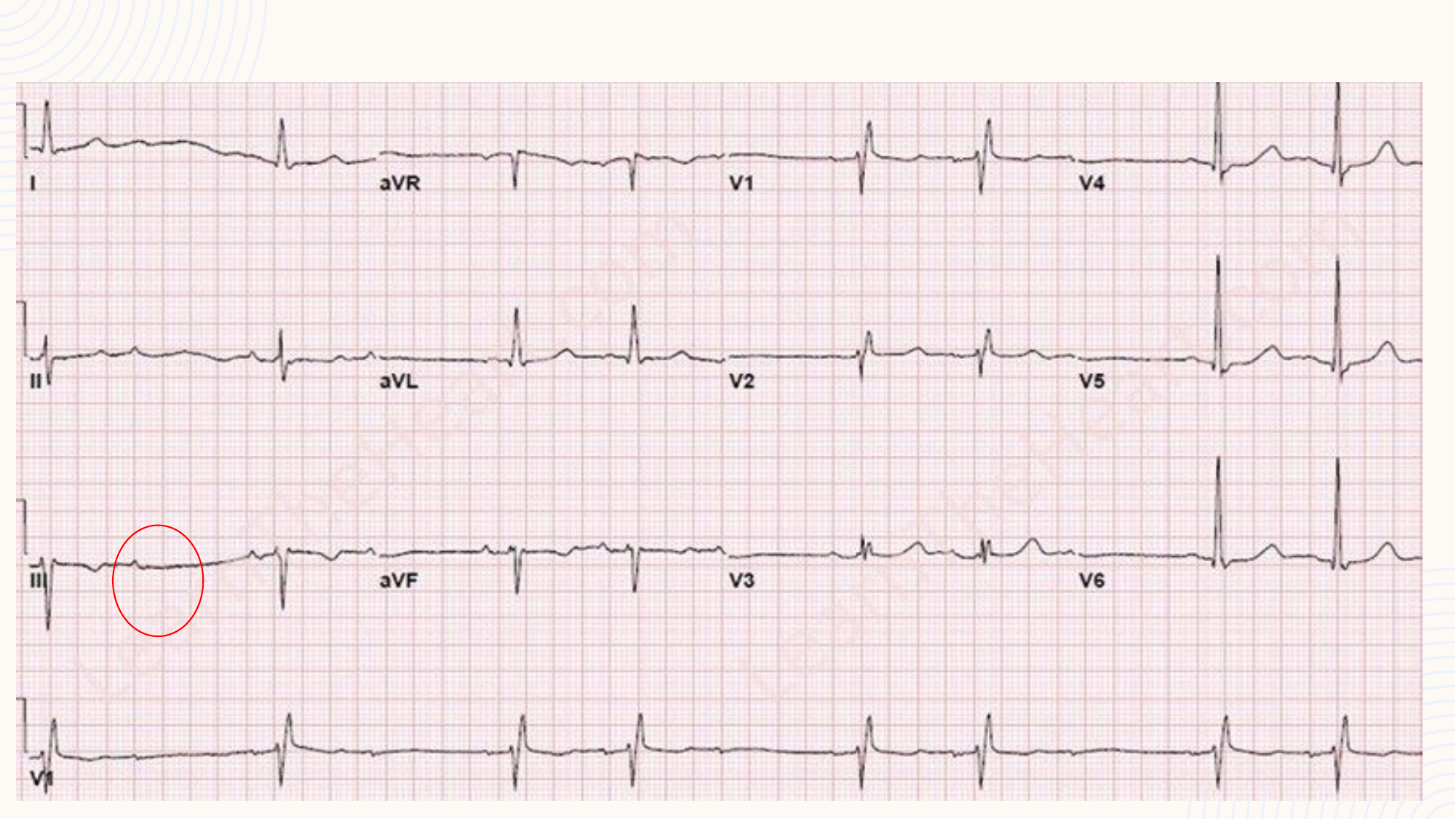

## Slide 9
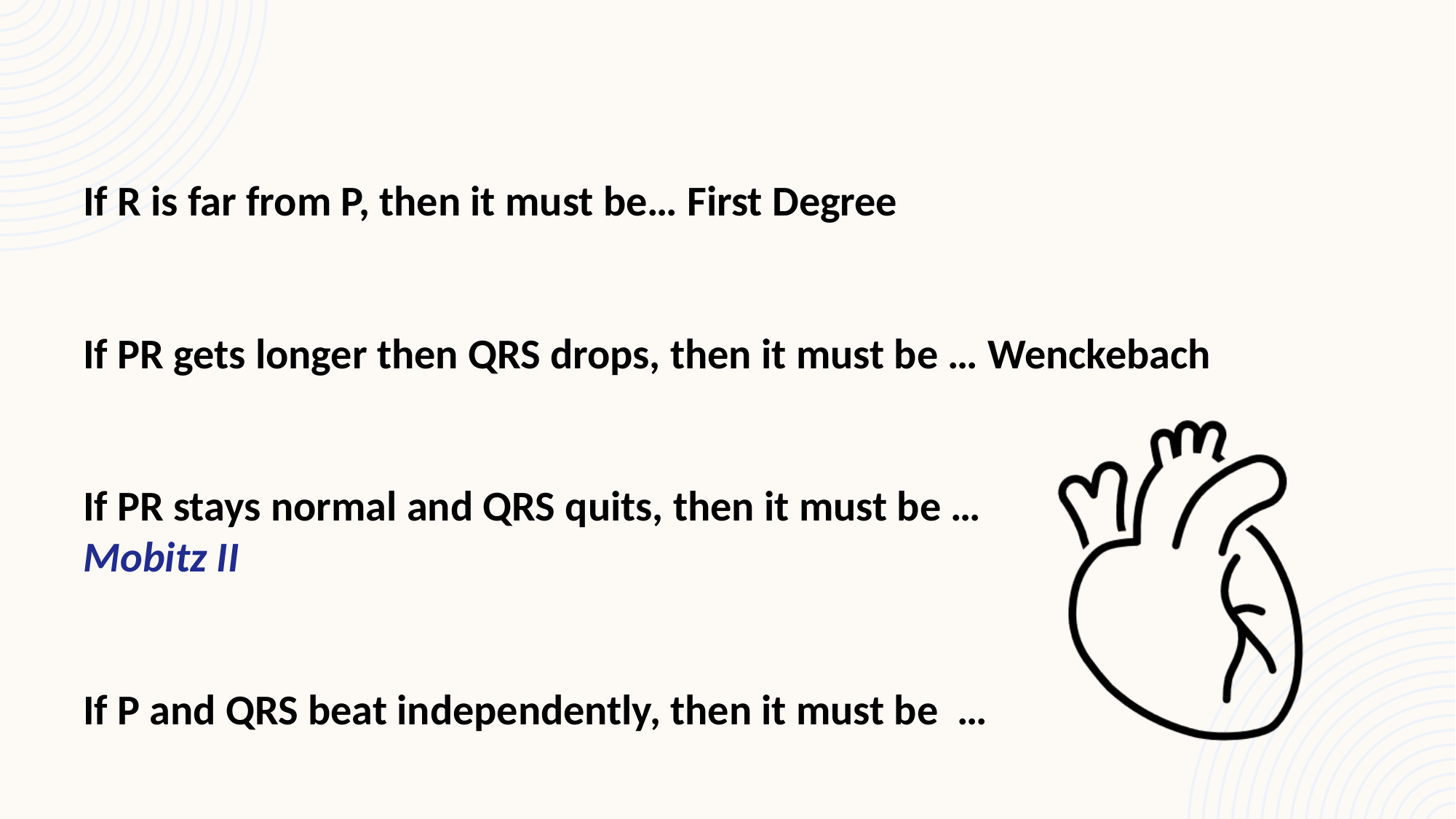

If R is far from P, then it must be… First Degree
If PR gets longer then QRS drops, then it must be … Wenckebach
If PR stays normal and QRS quits, then it must be …
Mobitz II
If P and QRS beat independently, then it must be …

## Slide 10
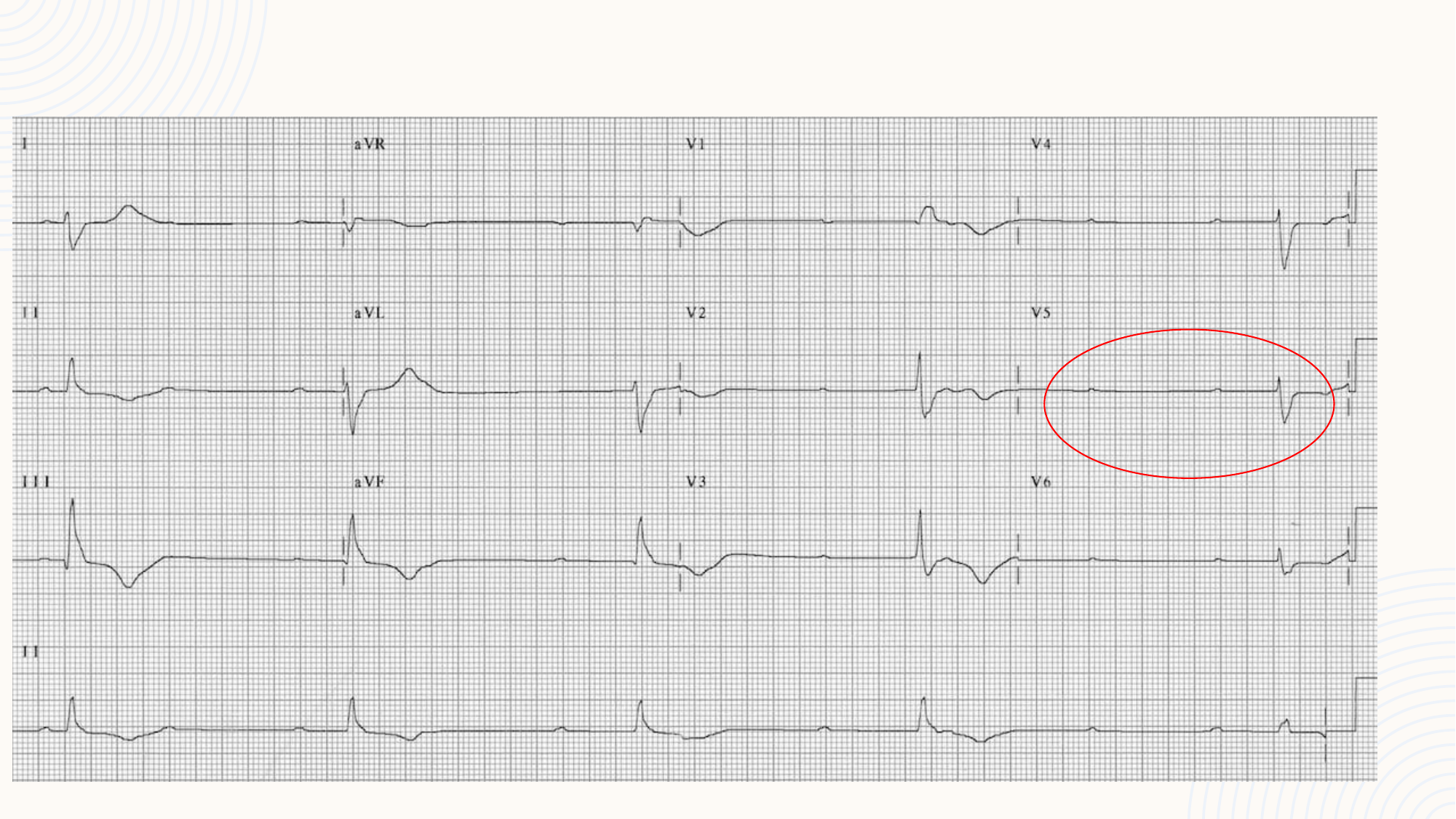

## Slide 11
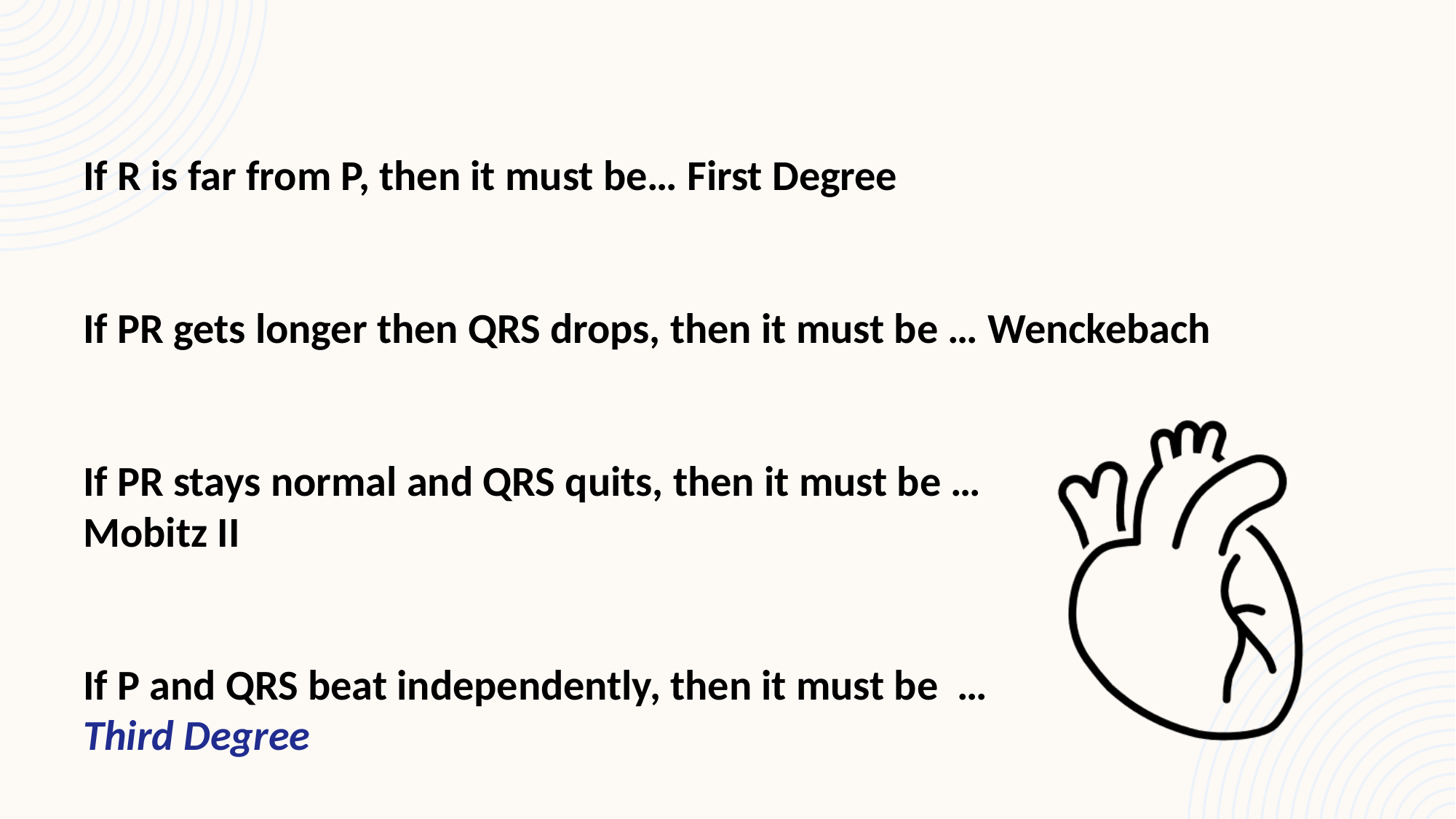

If R is far from P, then it must be… First Degree
If PR gets longer then QRS drops, then it must be … Wenckebach
If PR stays normal and QRS quits, then it must be …
Mobitz II
If P and QRS beat independently, then it must be …
Third Degree

## Slide 12
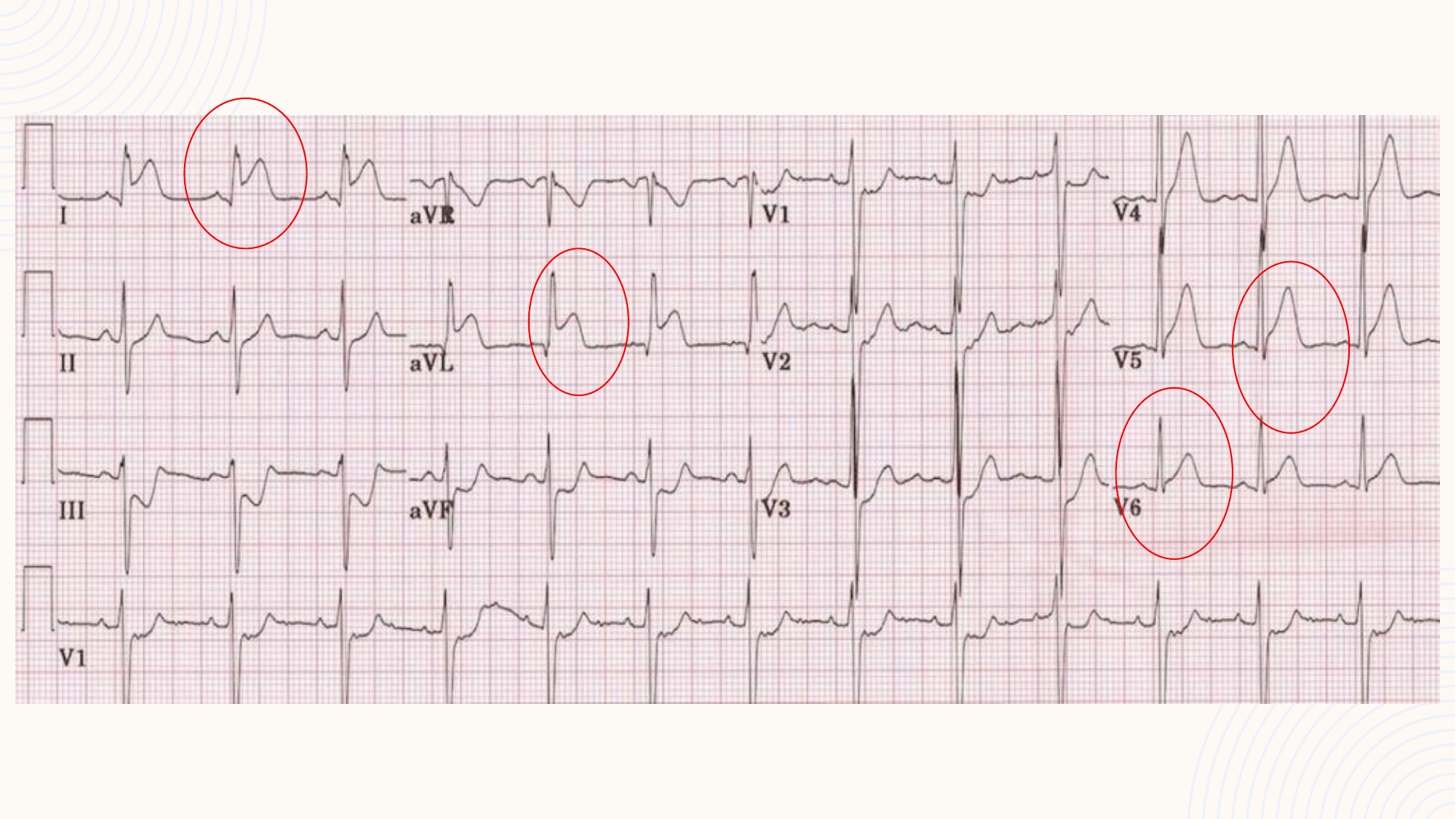

## Slide 13
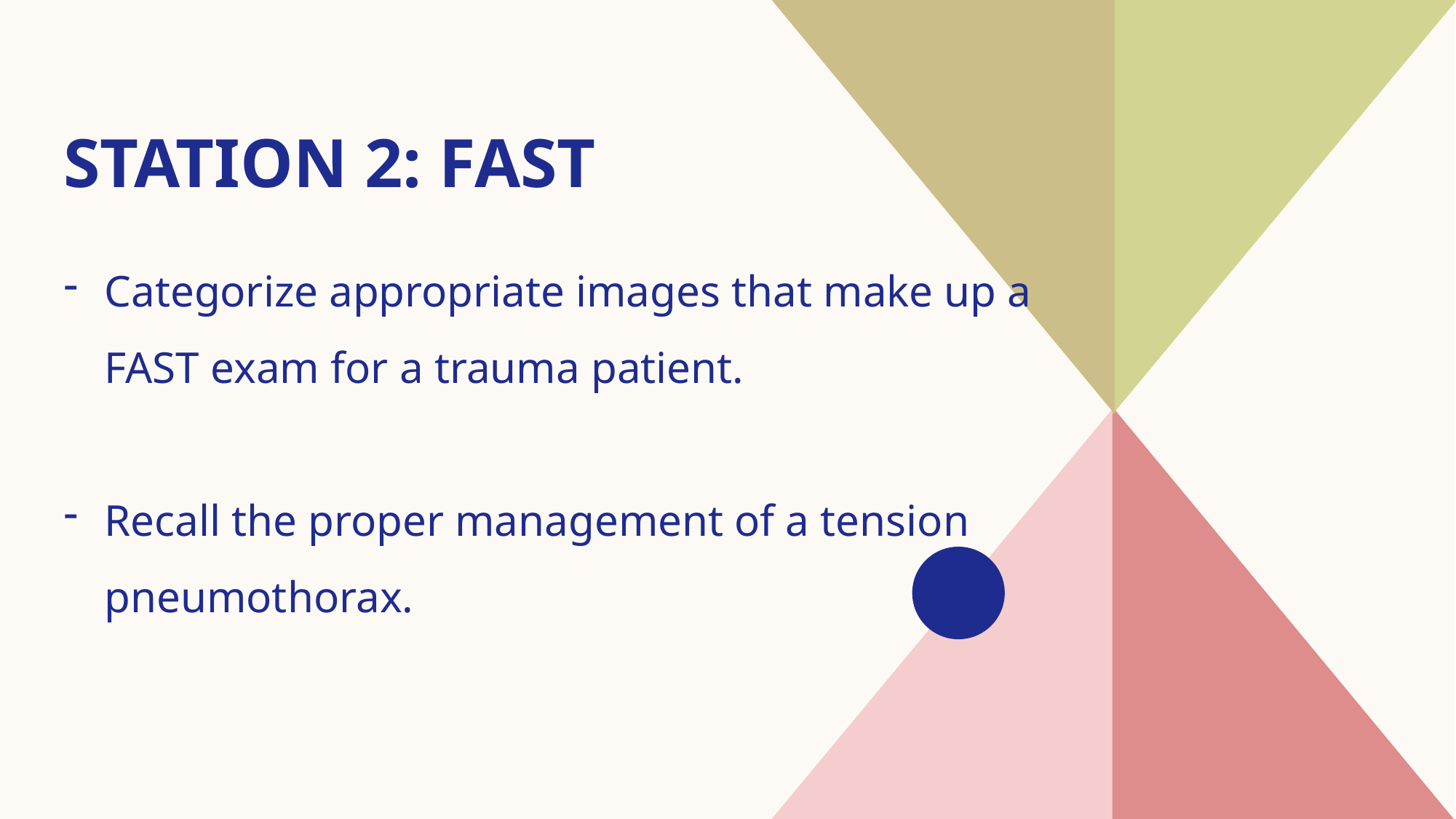

# Station 2: FAST
Categorize appropriate images that make up a FAST exam for a trauma patient.
Recall the proper management of a tension pneumothorax.

## Slide 14
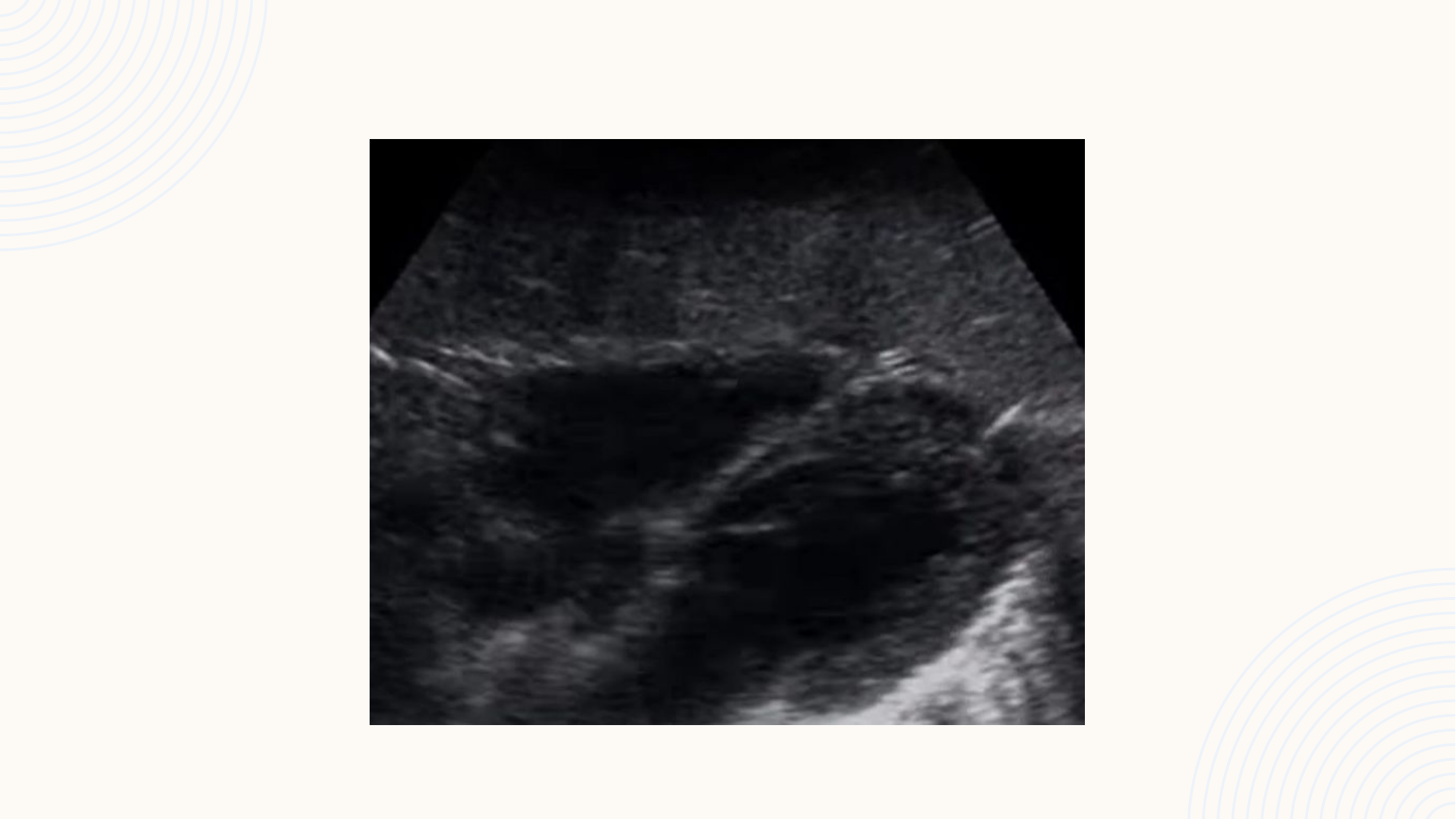

## Slide 15
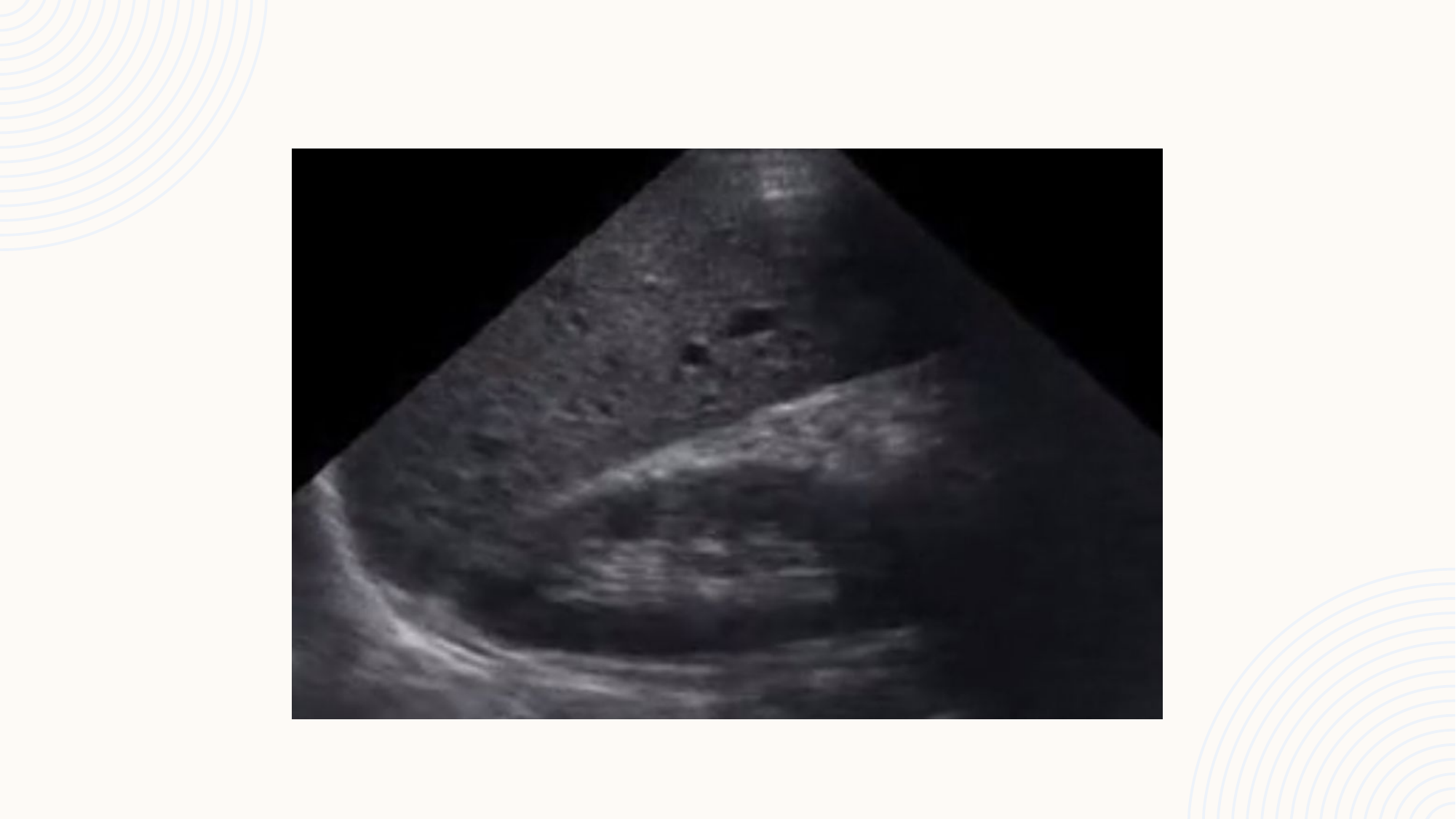

## Slide 16
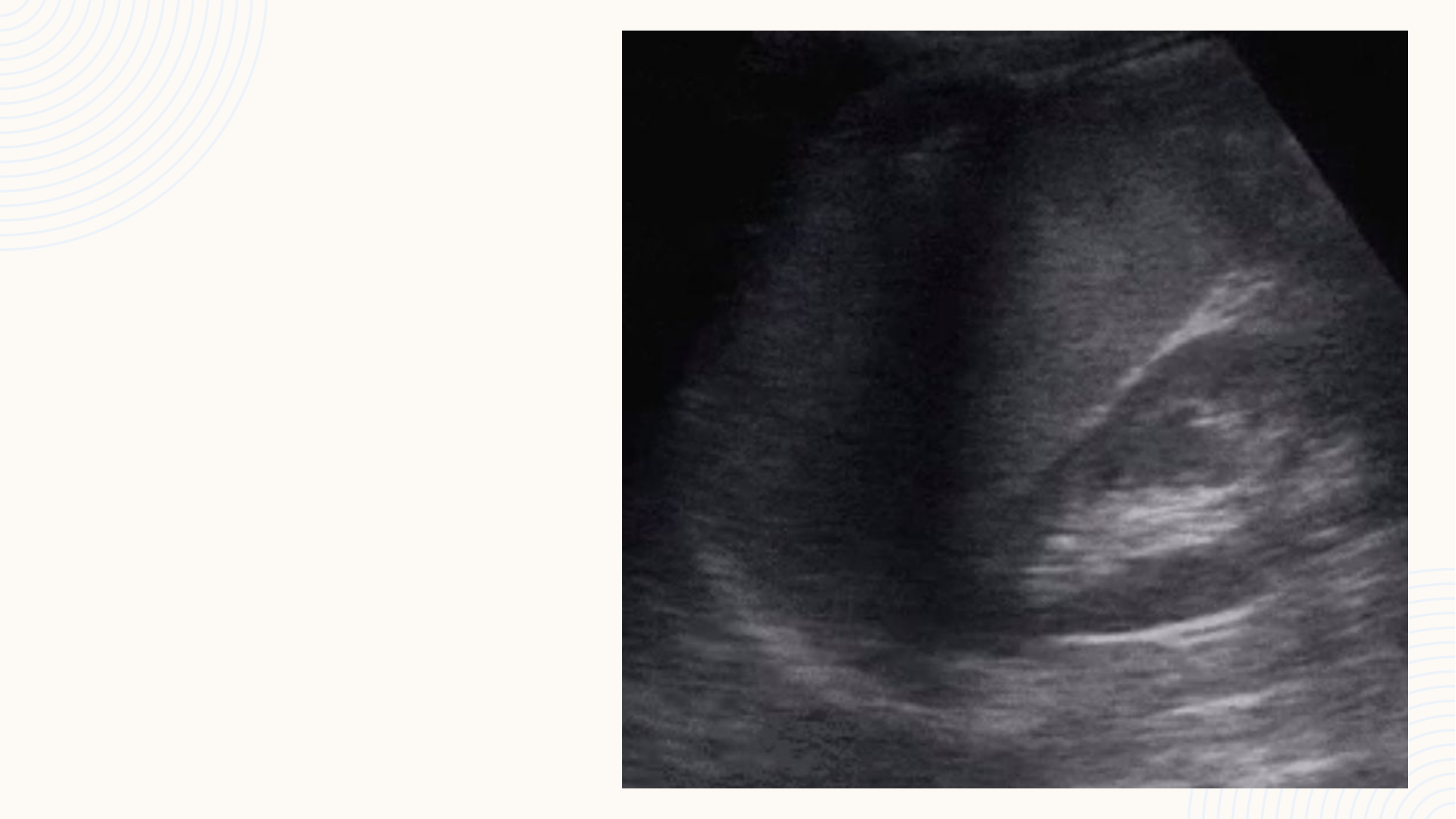

## Slide 17
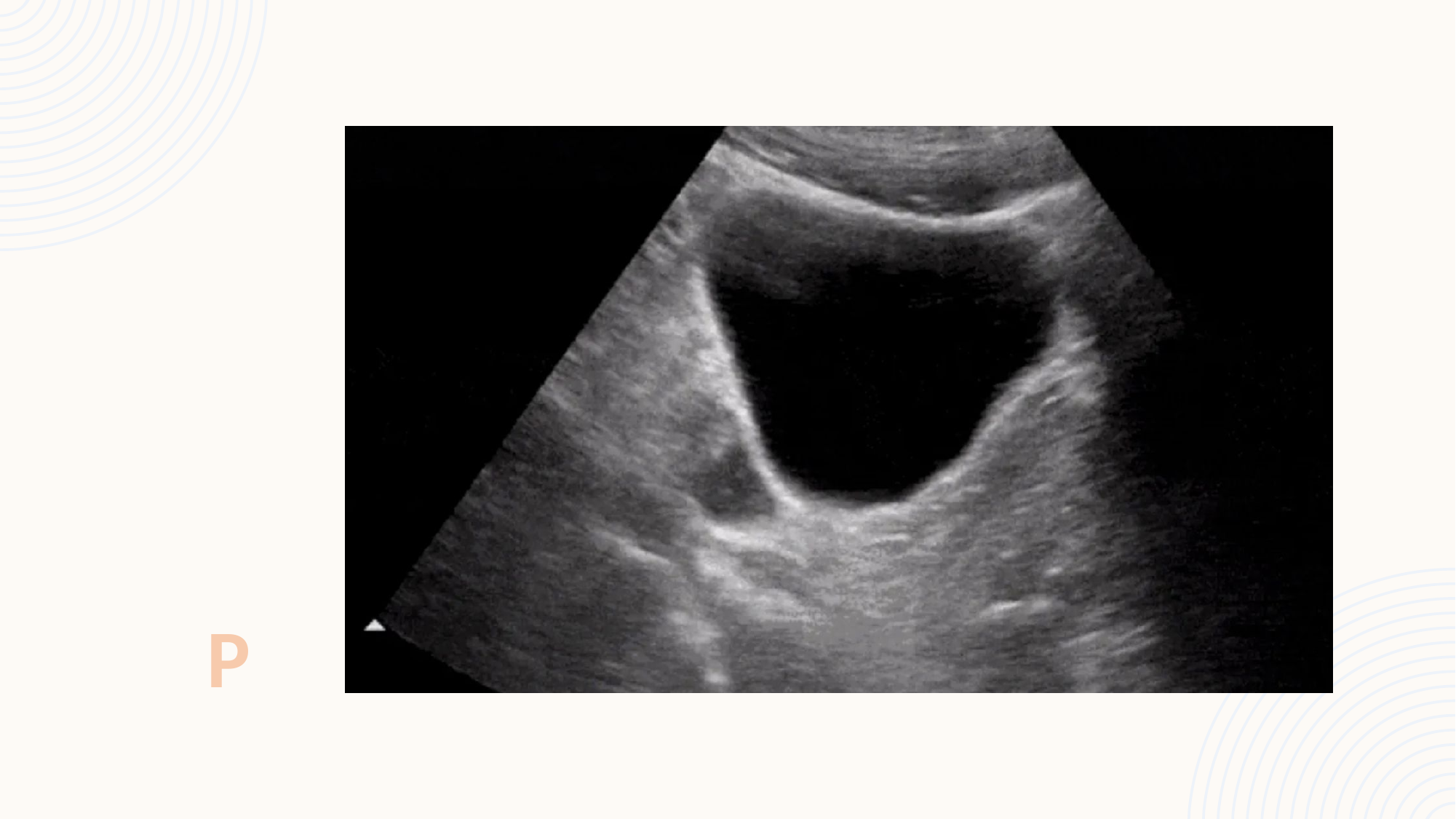

P

## Slide 18
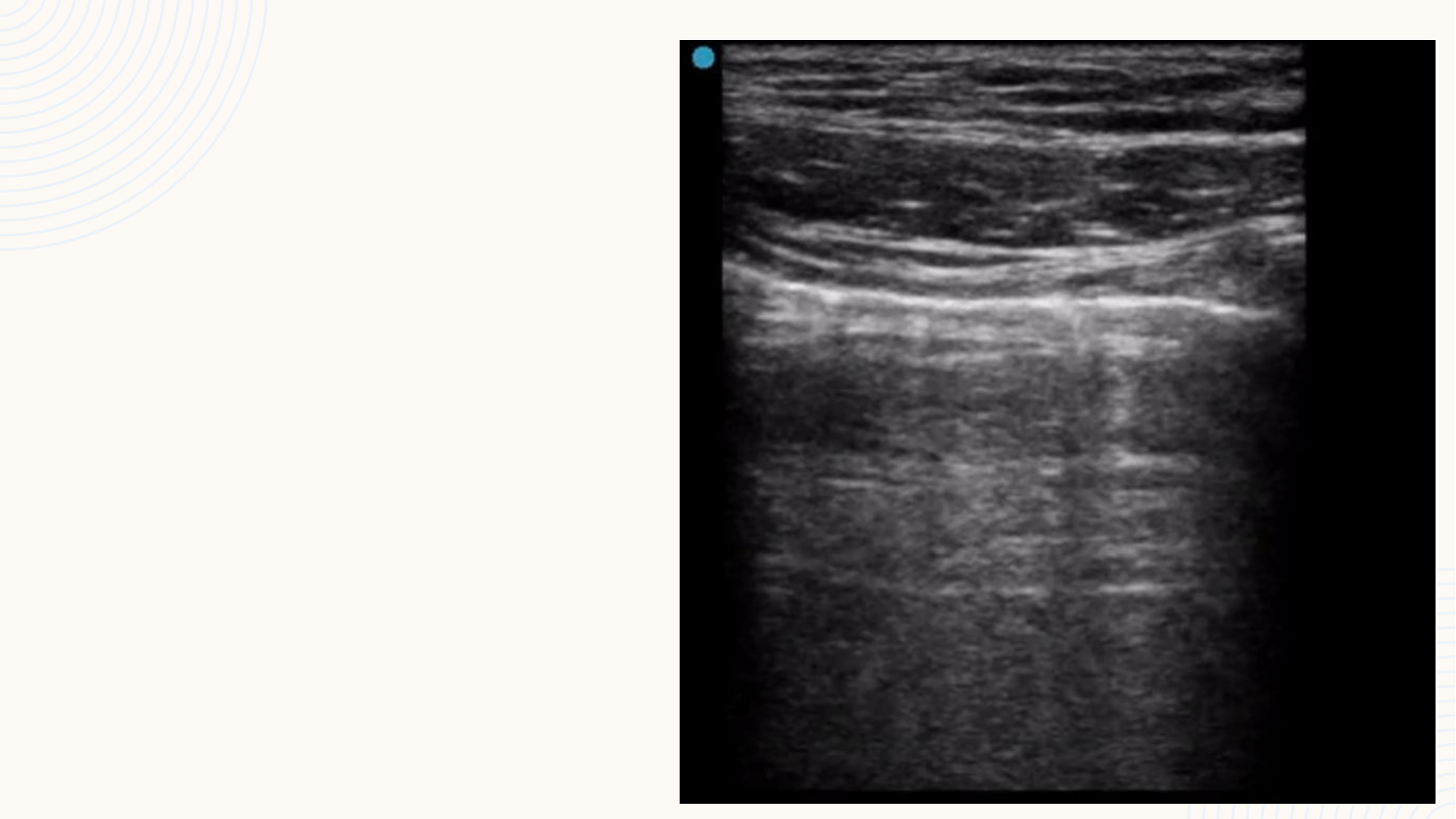

## Slide 19
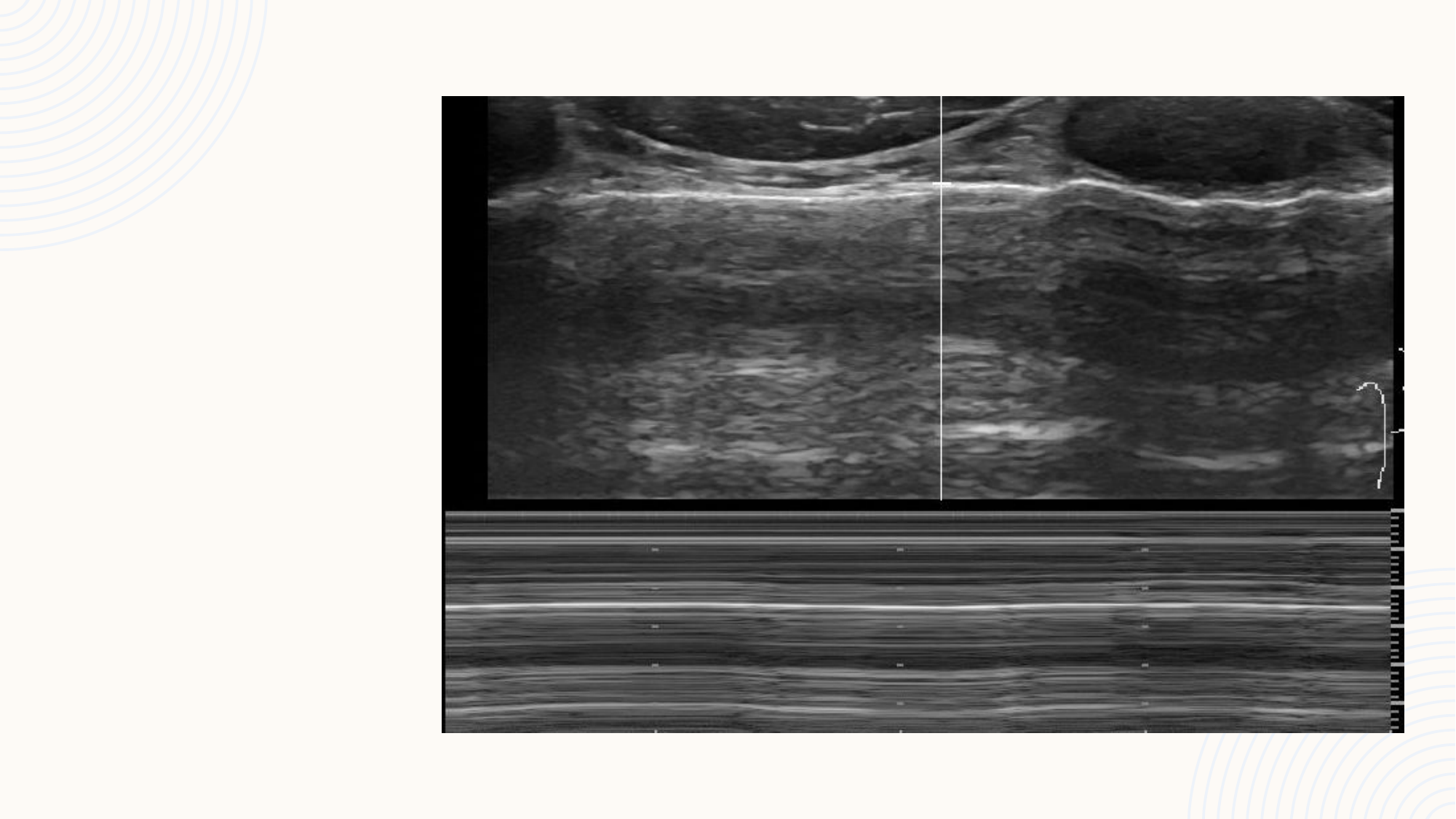

## Slide 20
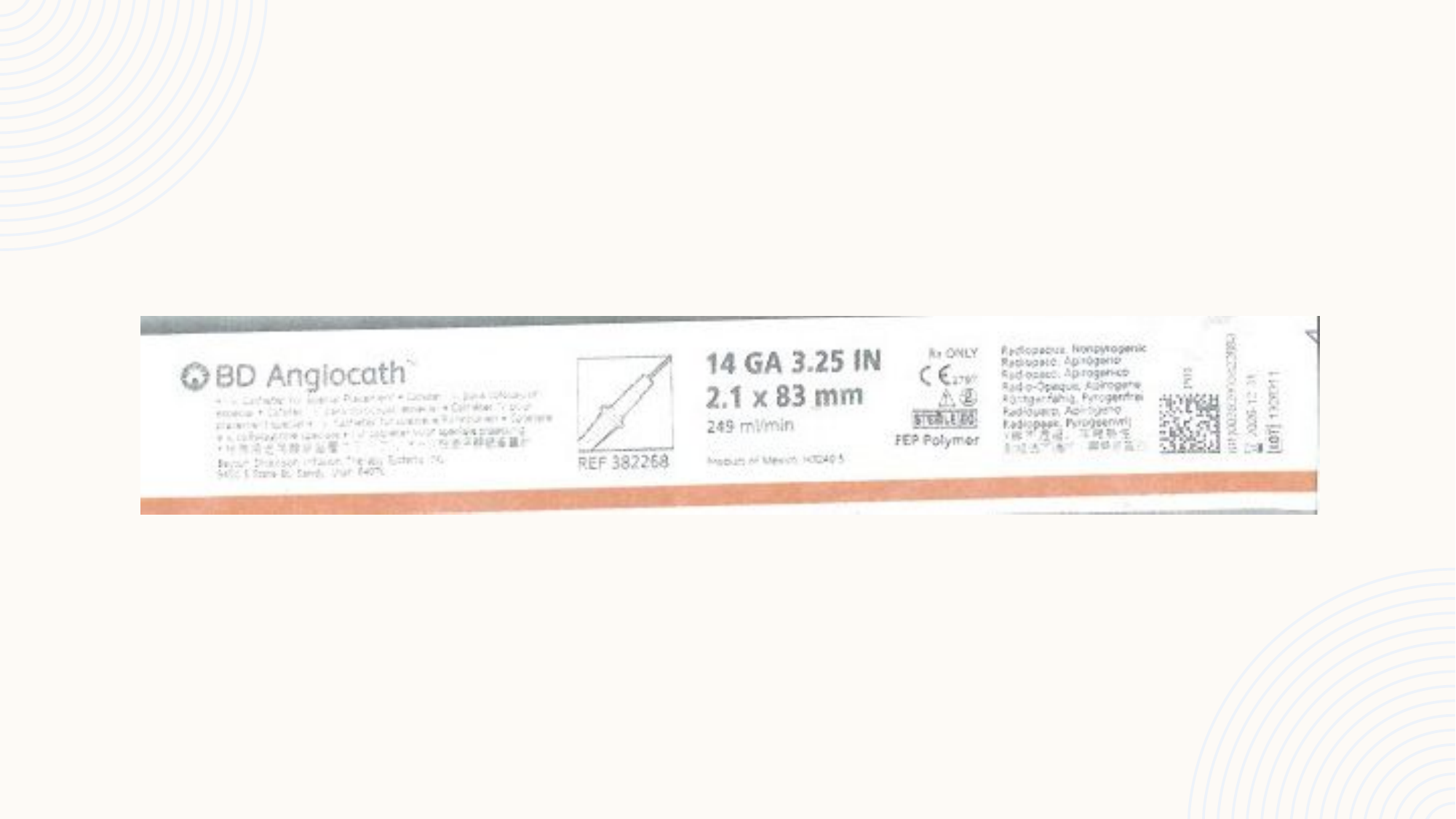

## Slide 21
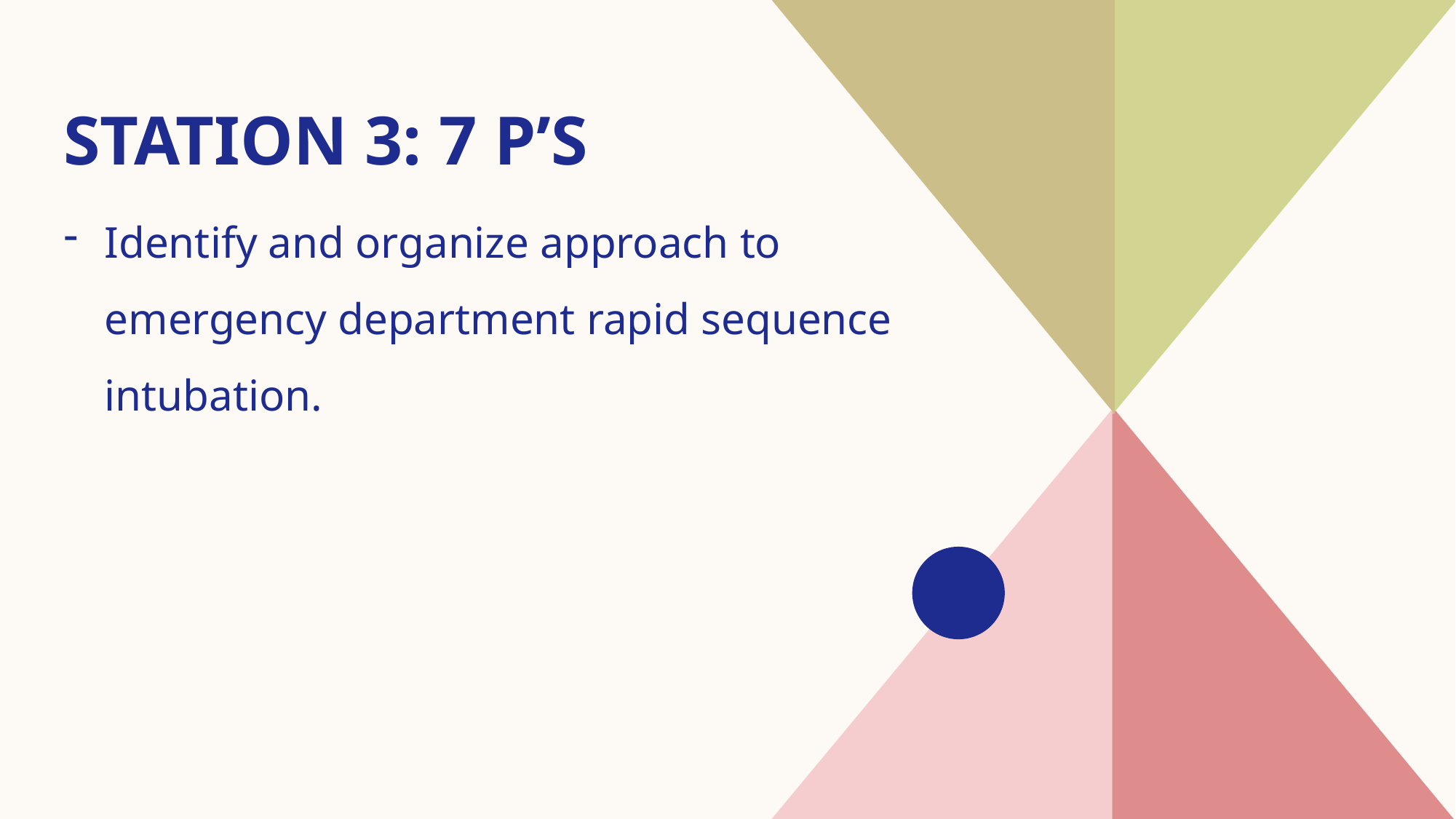

# Station 3: 7 P’s
Identify and organize approach to emergency department rapid sequence intubation.

## Slide 22
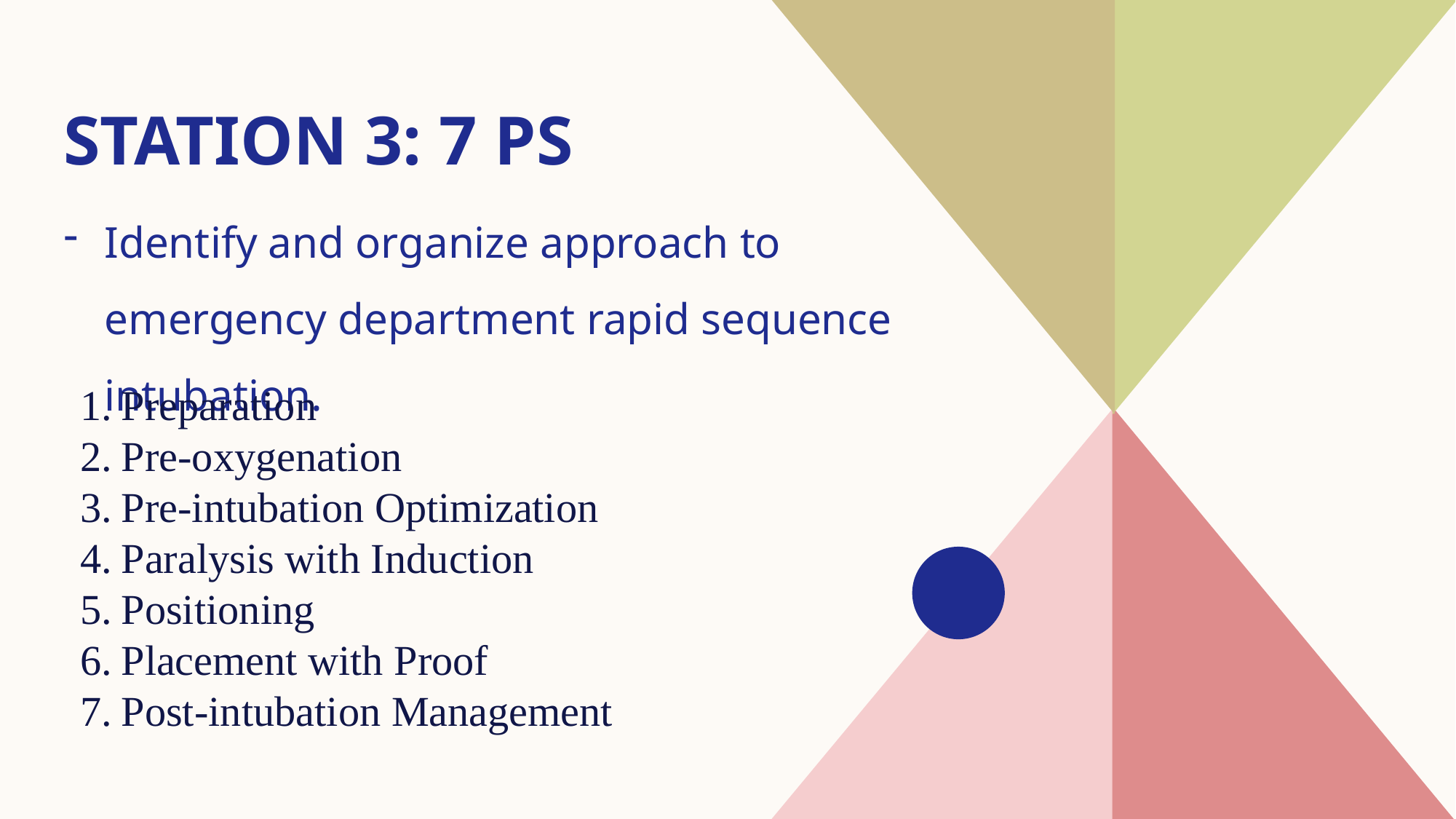

# Station 3: 7 Ps
Identify and organize approach to emergency department rapid sequence intubation.
Preparation
Pre-oxygenation
Pre-intubation Optimization
Paralysis with Induction
Positioning
Placement with Proof
Post-intubation Management

## Slide 23
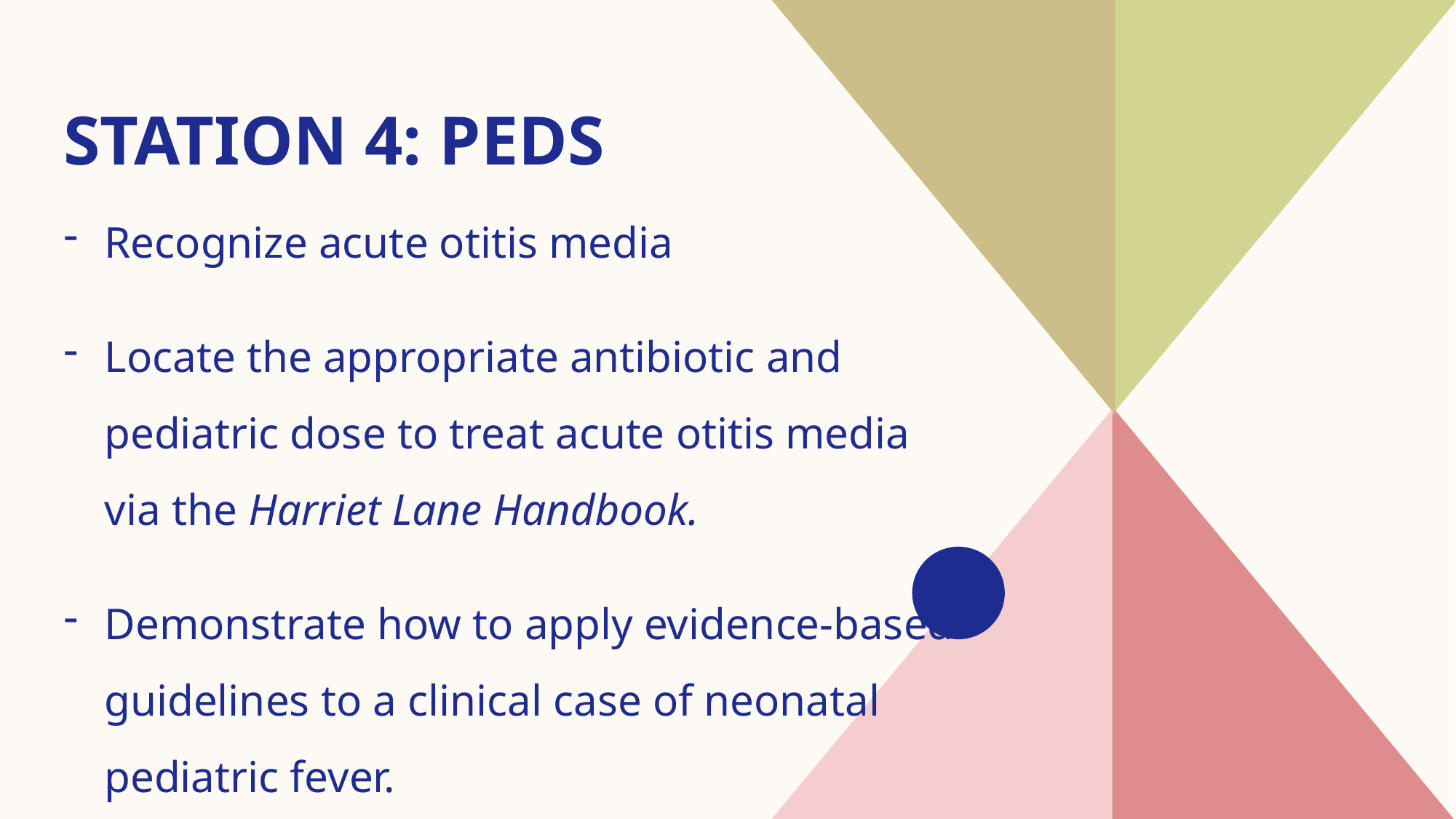

# Station 4: Peds
Recognize acute otitis media
Locate the appropriate antibiotic and pediatric dose to treat acute otitis media via the Harriet Lane Handbook.
Demonstrate how to apply evidence-based guidelines to a clinical case of neonatal pediatric fever.

## Slide 24
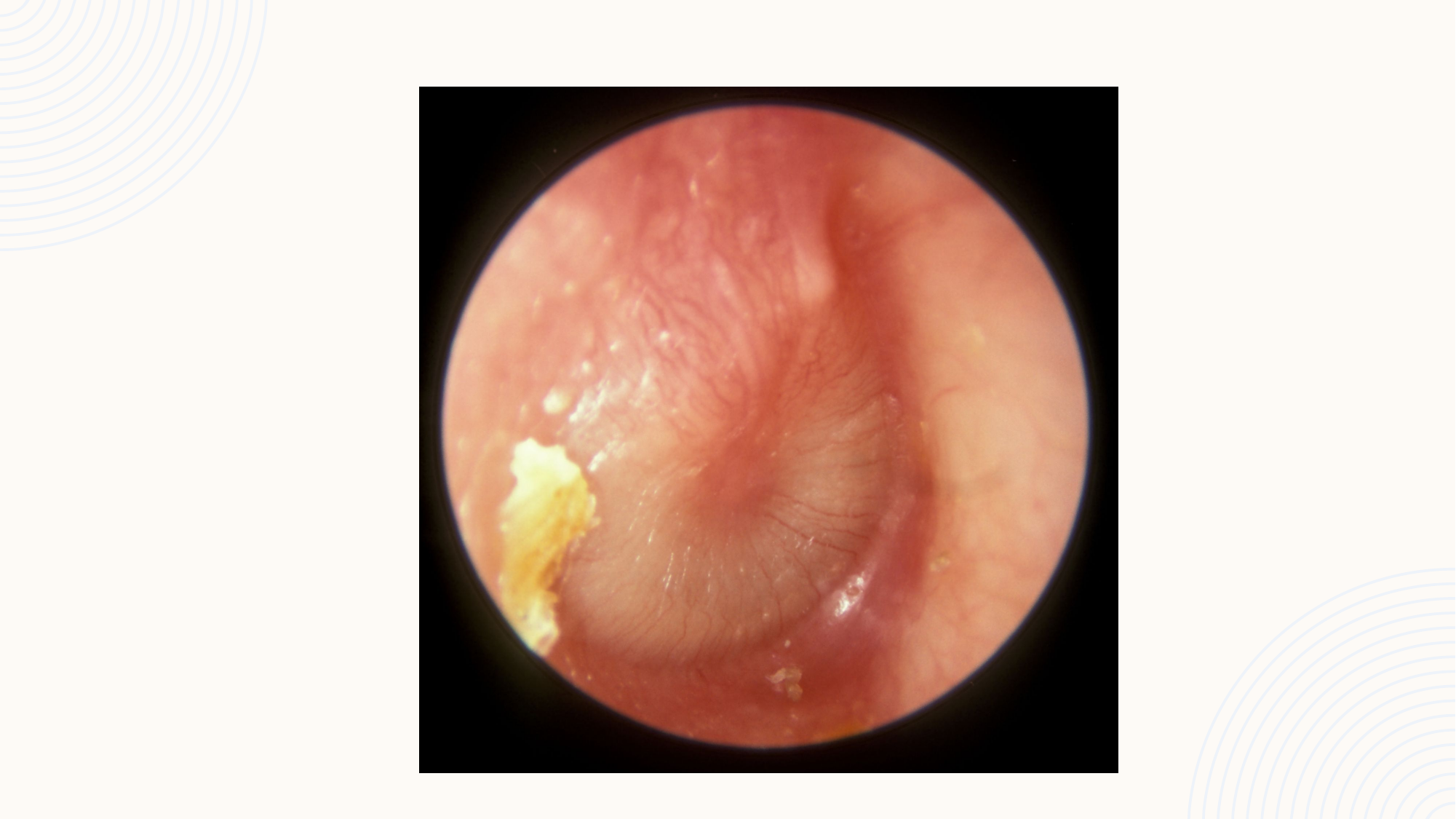

## Slide 25
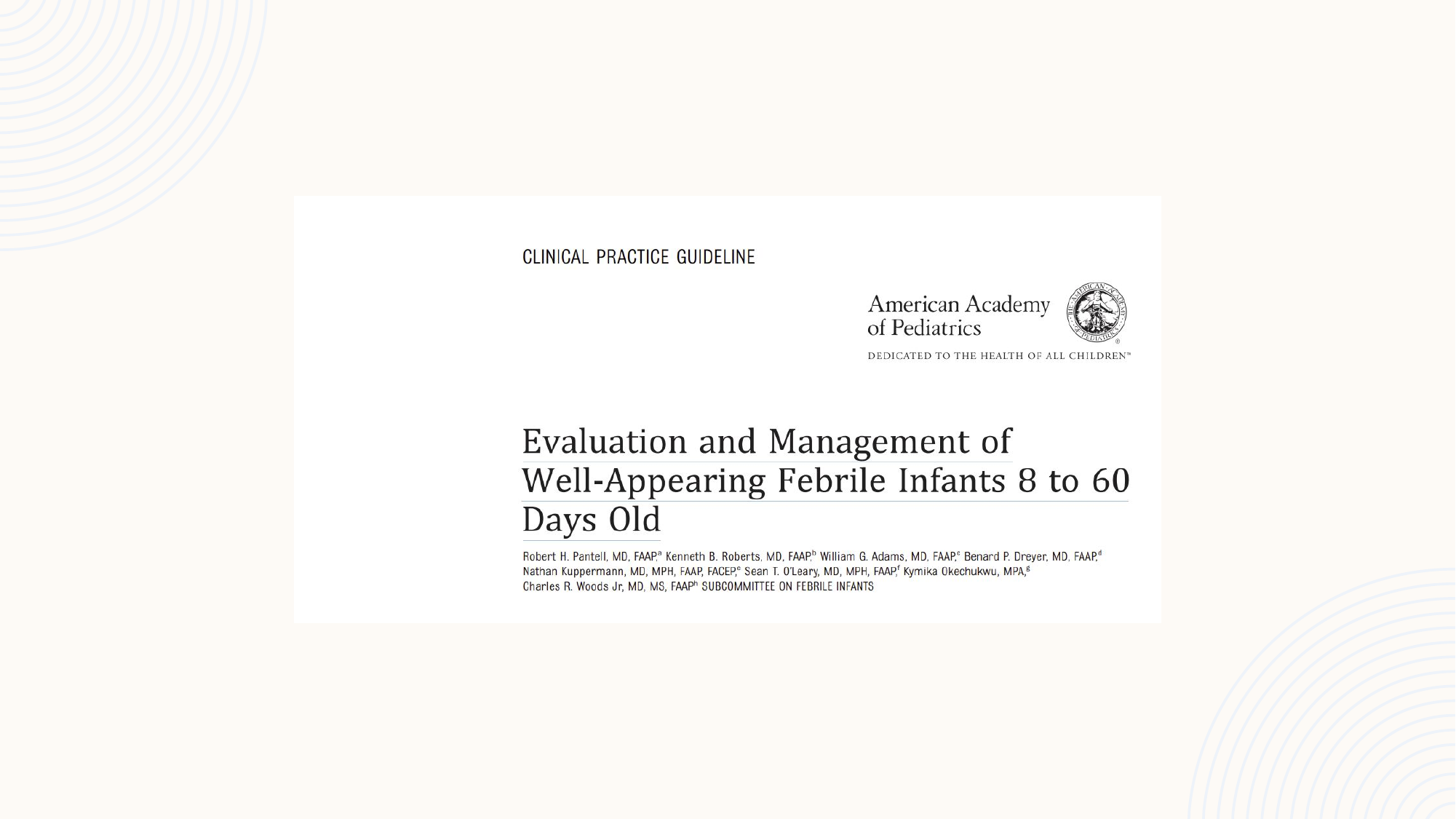

## Slide 26
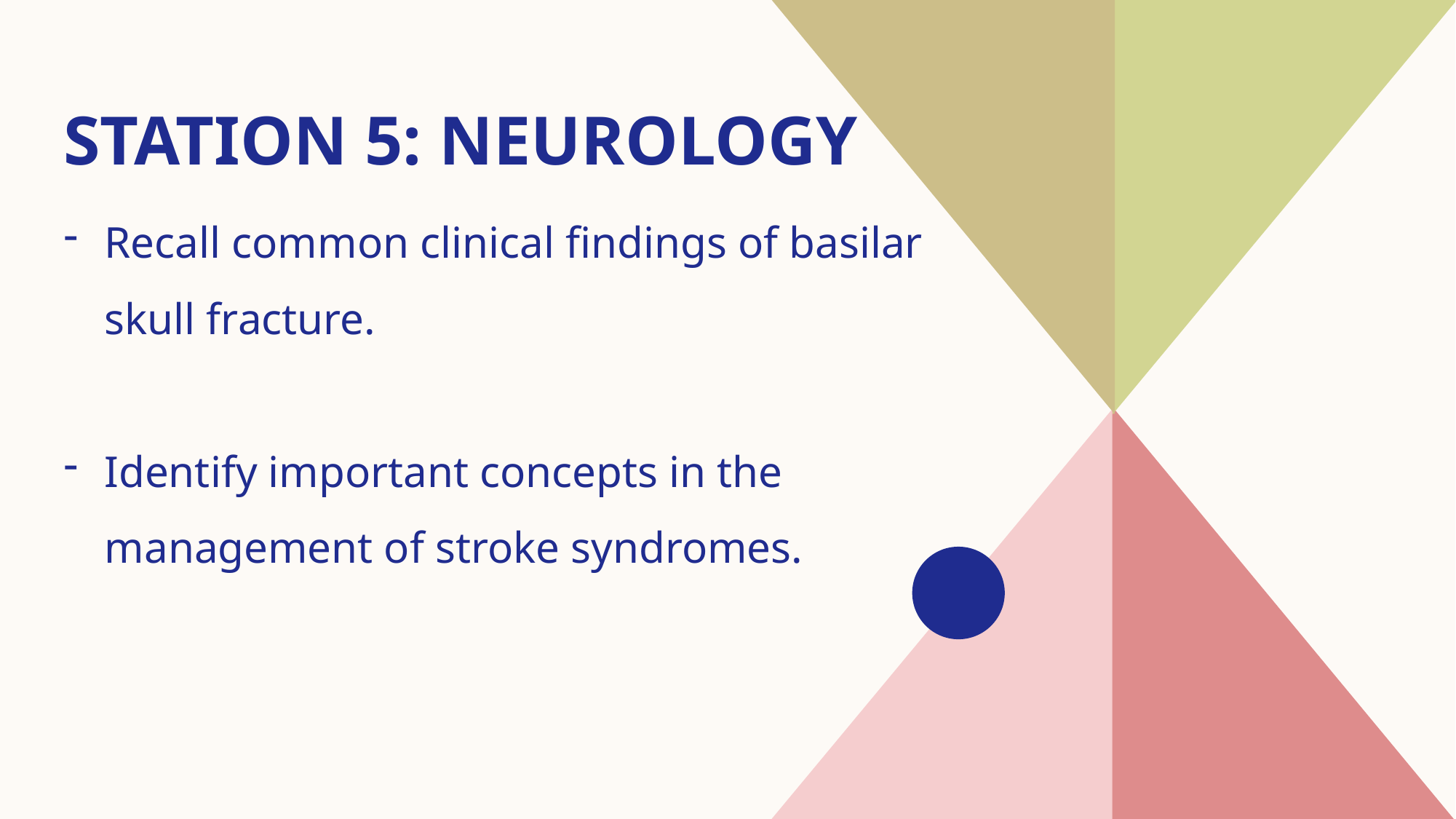

# Station 5: Neurology
Recall common clinical findings of basilar skull fracture.
Identify important concepts in the management of stroke syndromes.

## Slide 27
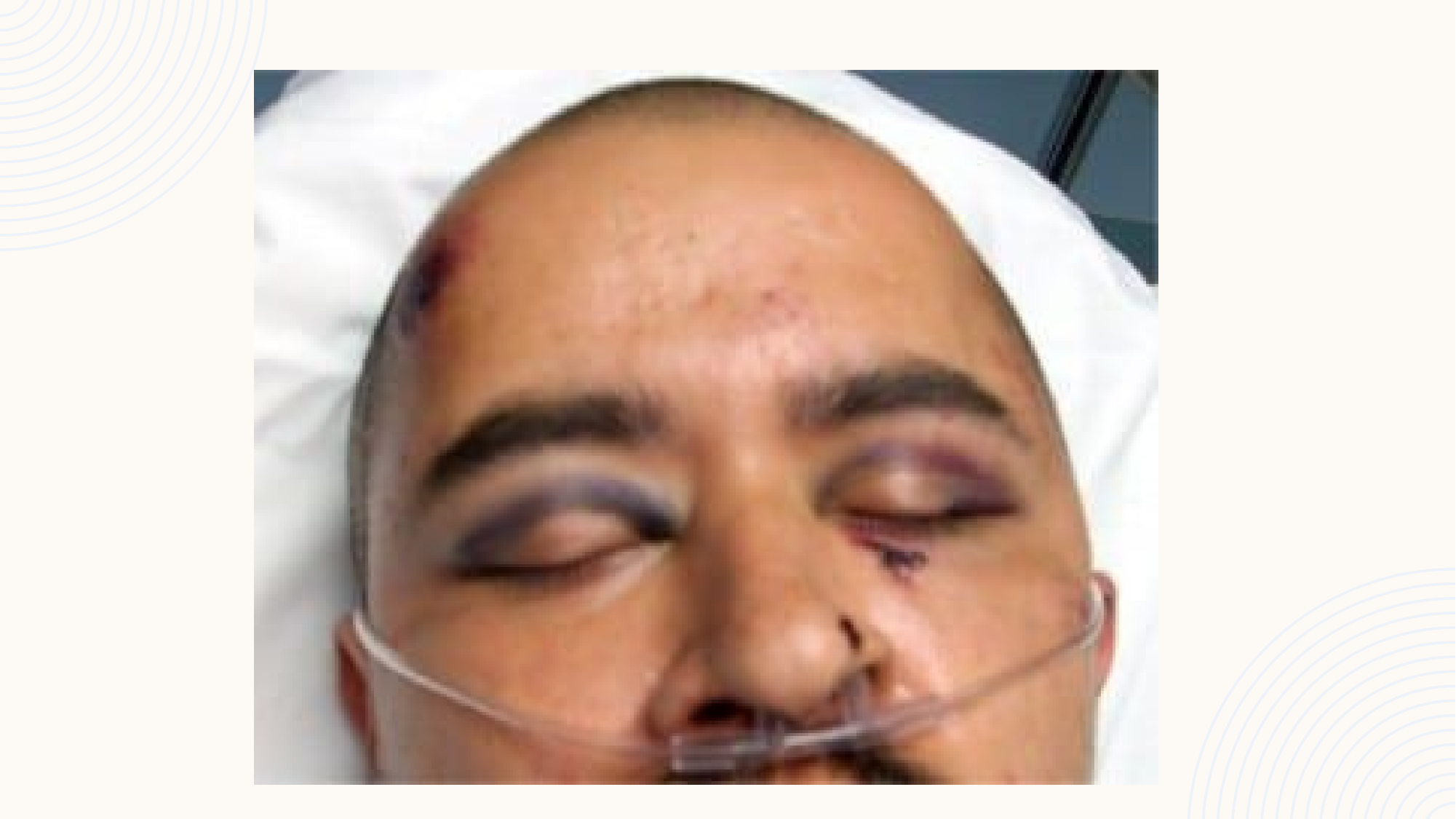

## Slide 28
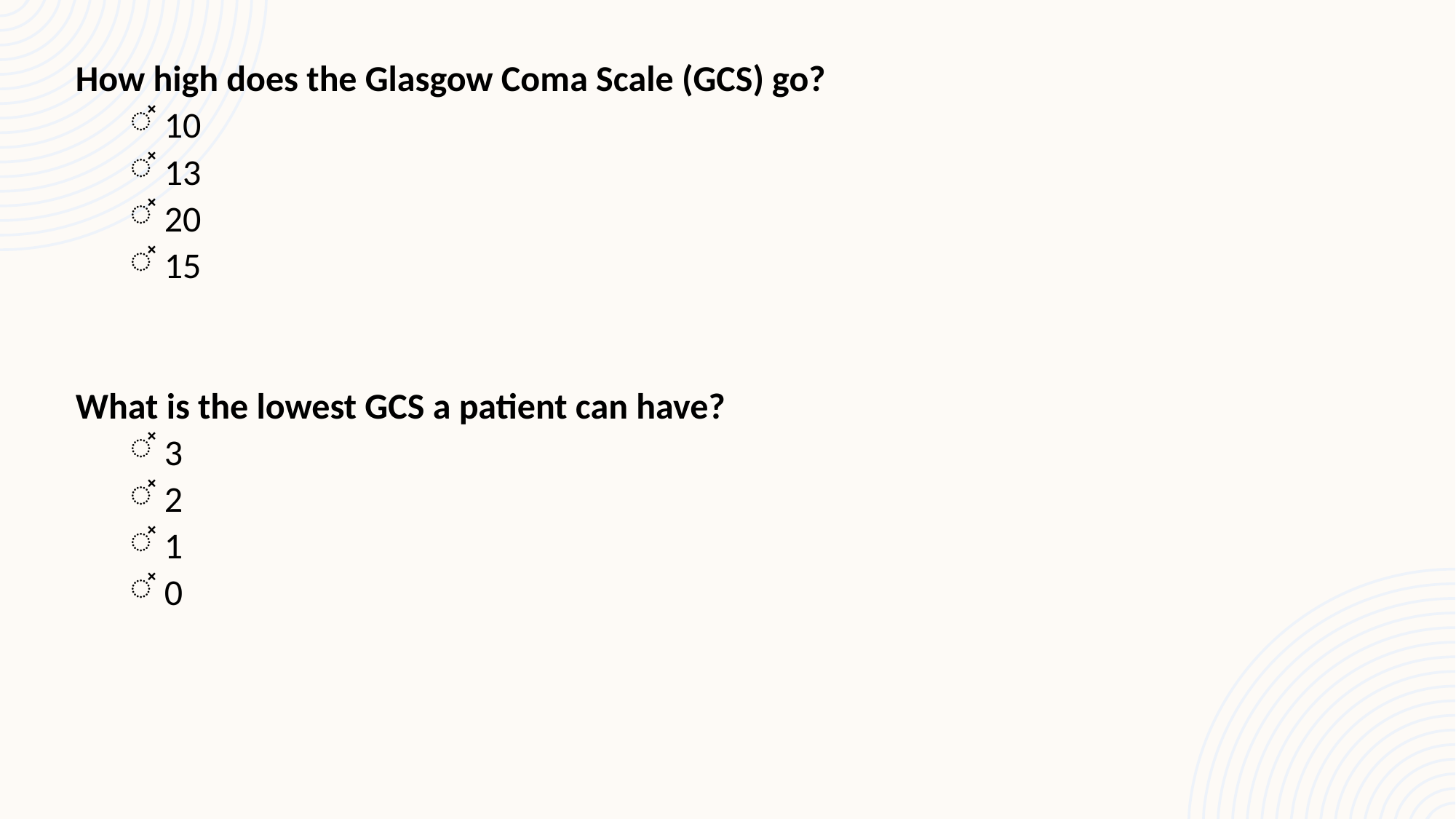

How high does the Glasgow Coma Scale (GCS) go?
10
13
20
15
What is the lowest GCS a patient can have?
3
2
1
0

## Slide 29
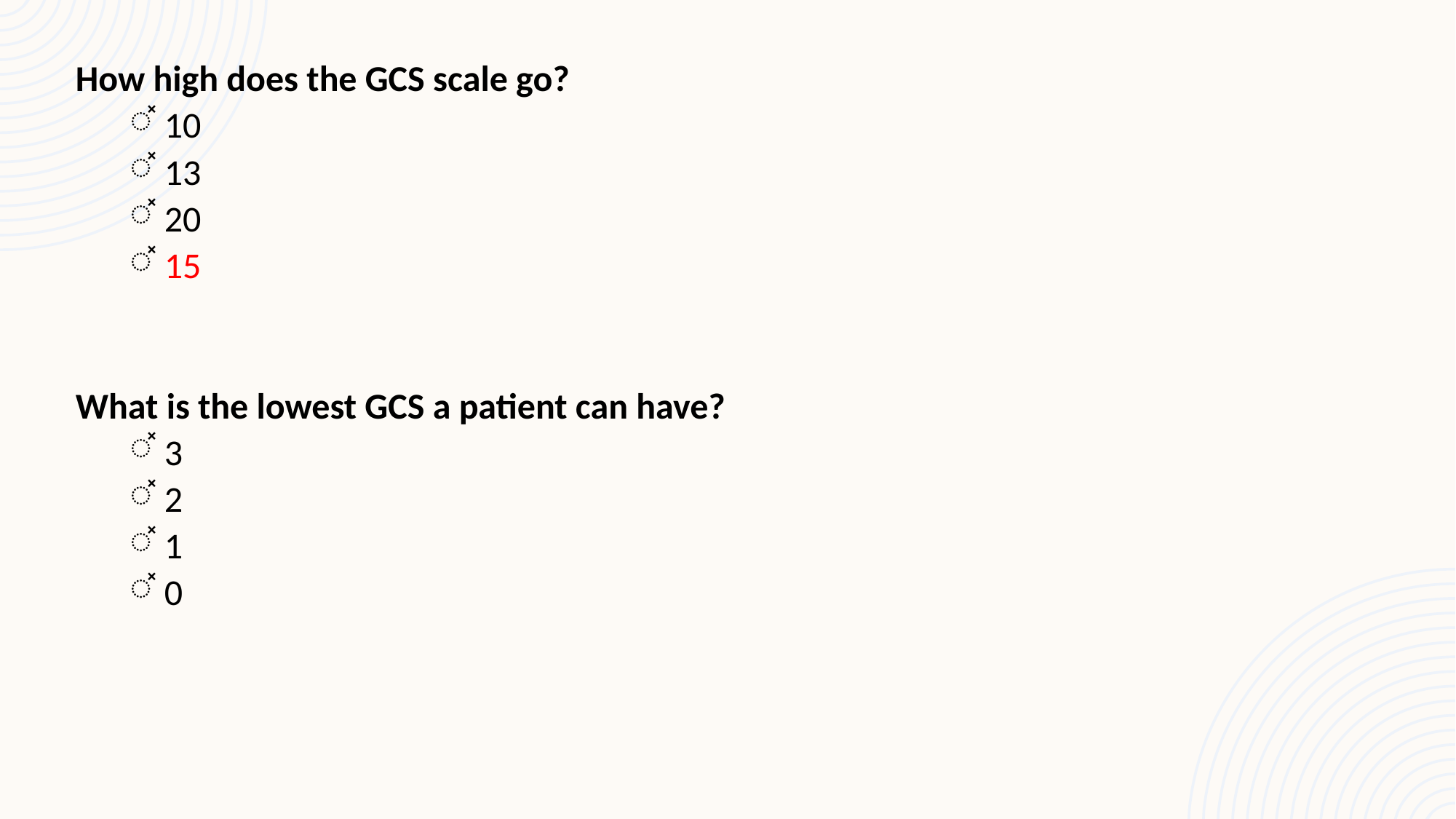

How high does the GCS scale go?
10
13
20
15
What is the lowest GCS a patient can have?
3
2
1
0

## Slide 30
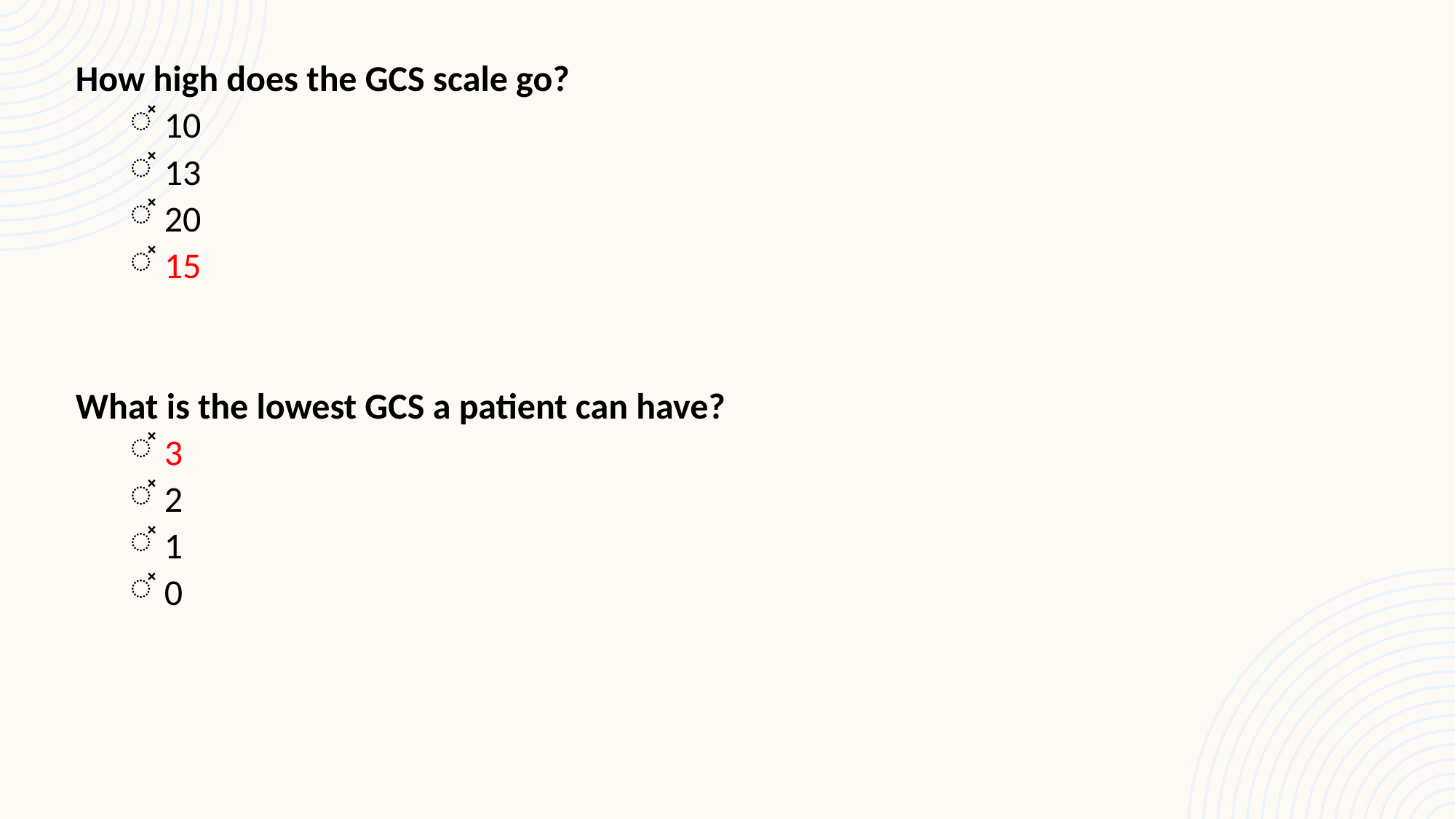

How high does the GCS scale go?
10
13
20
15
What is the lowest GCS a patient can have?
3
2
1
0

## Slide 31
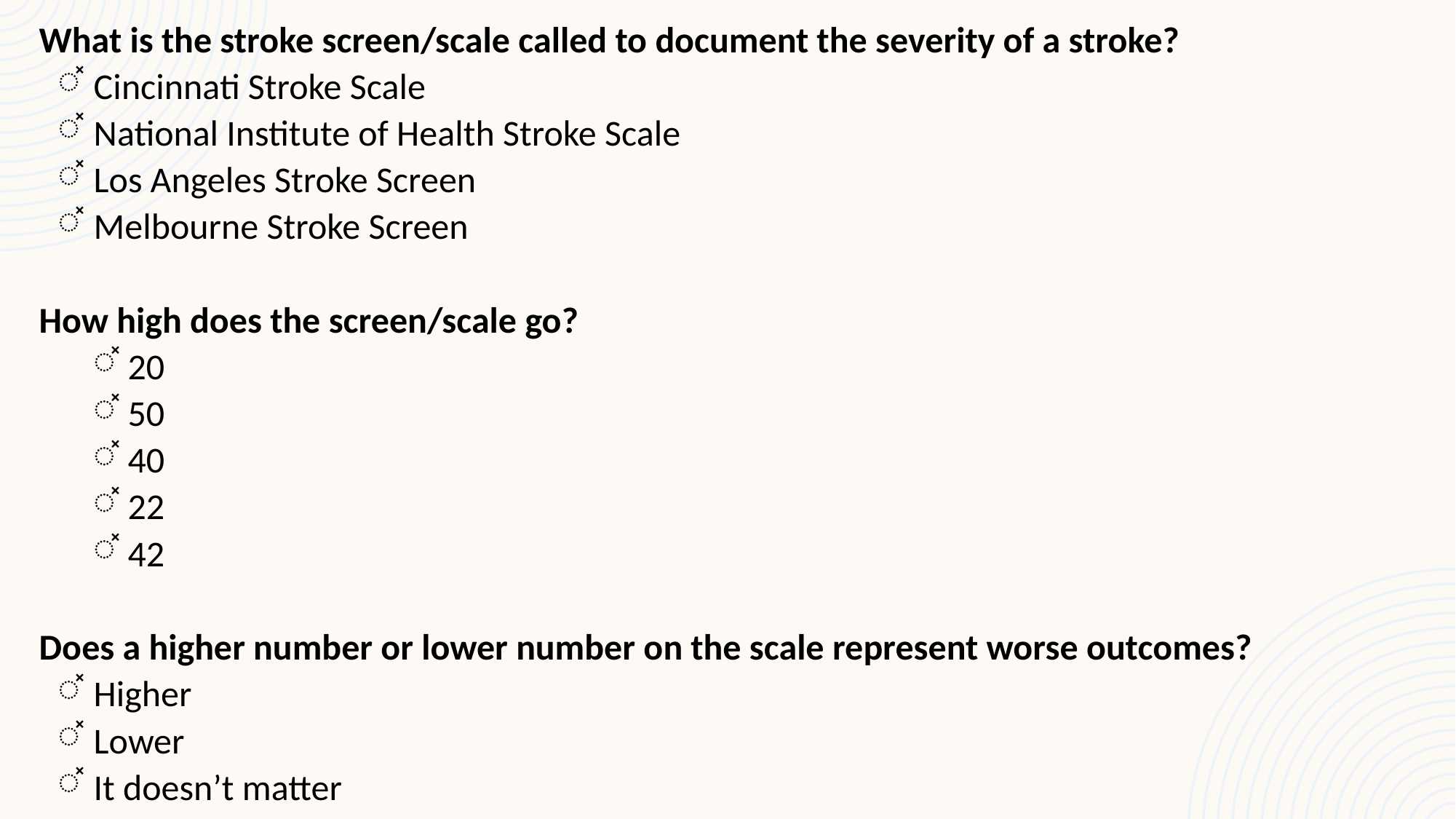

What is the stroke screen/scale called to document the severity of a stroke?
Cincinnati Stroke Scale
National Institute of Health Stroke Scale
Los Angeles Stroke Screen
Melbourne Stroke Screen
How high does the screen/scale go?
20
50
40
22
42
Does a higher number or lower number on the scale represent worse outcomes?
Higher
Lower
It doesn’t matter

## Slide 32
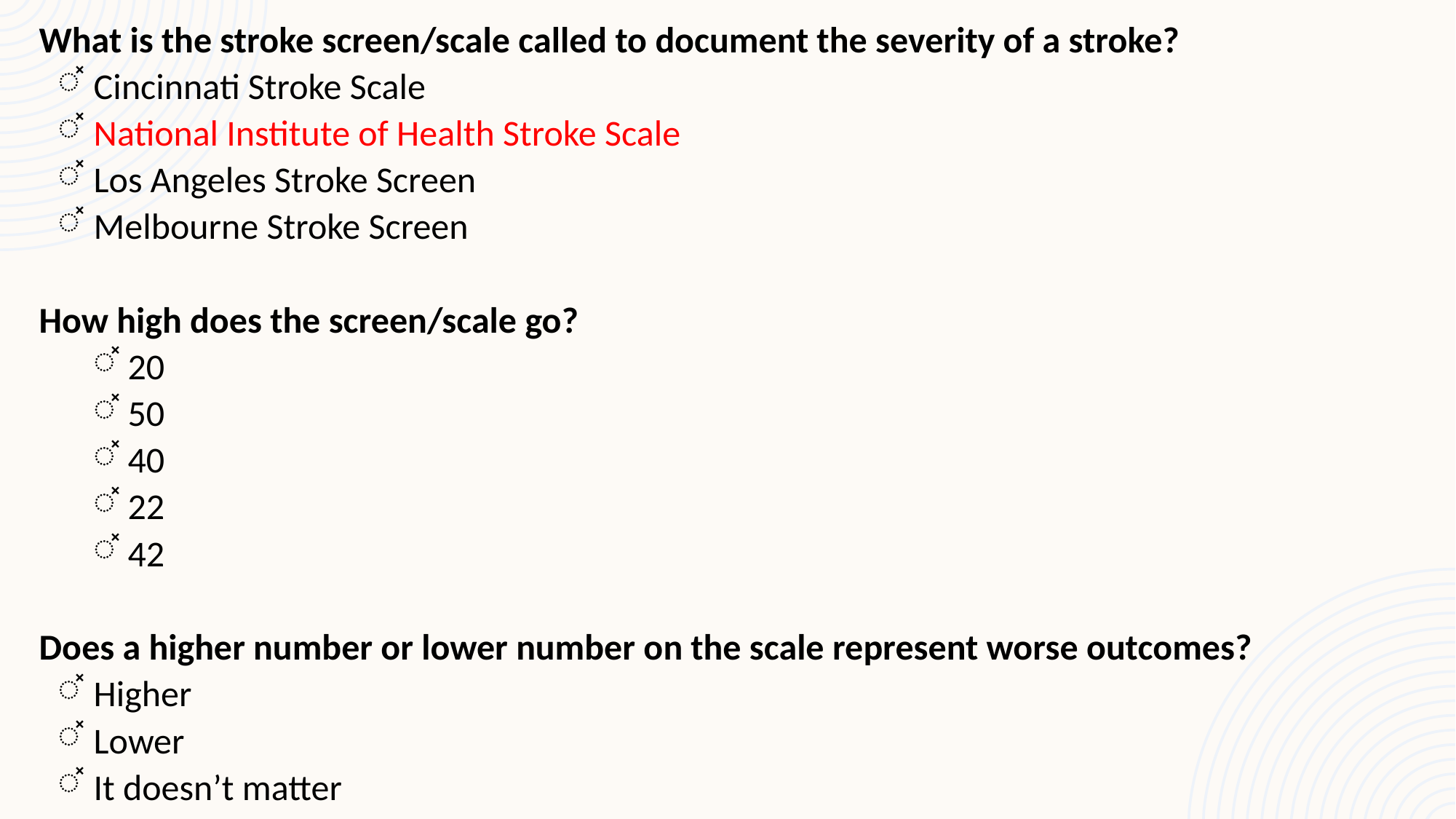

What is the stroke screen/scale called to document the severity of a stroke?
Cincinnati Stroke Scale
National Institute of Health Stroke Scale
Los Angeles Stroke Screen
Melbourne Stroke Screen
How high does the screen/scale go?
20
50
40
22
42
Does a higher number or lower number on the scale represent worse outcomes?
Higher
Lower
It doesn’t matter

## Slide 33
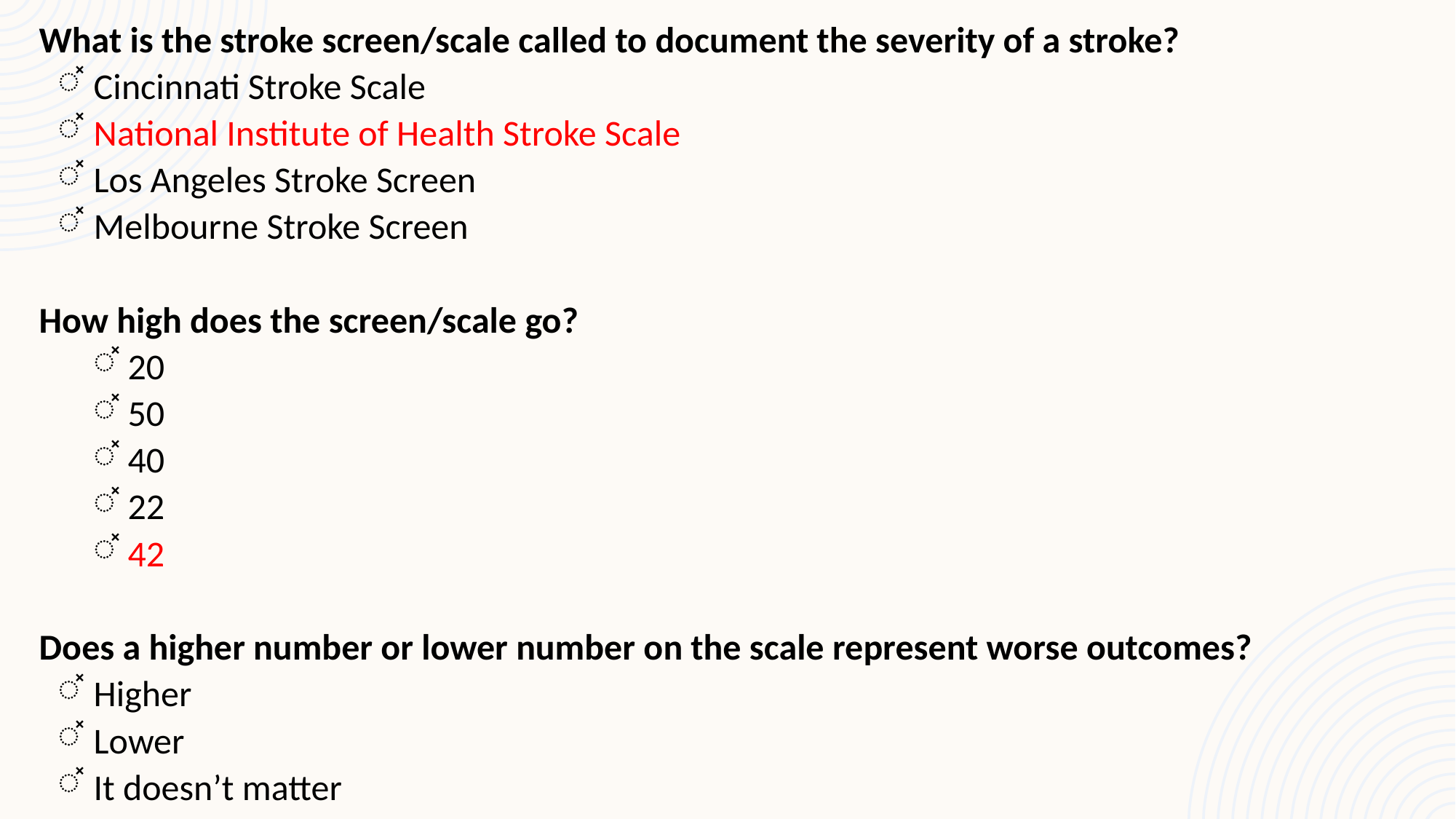

What is the stroke screen/scale called to document the severity of a stroke?
Cincinnati Stroke Scale
National Institute of Health Stroke Scale
Los Angeles Stroke Screen
Melbourne Stroke Screen
How high does the screen/scale go?
20
50
40
22
42
Does a higher number or lower number on the scale represent worse outcomes?
Higher
Lower
It doesn’t matter

## Slide 34
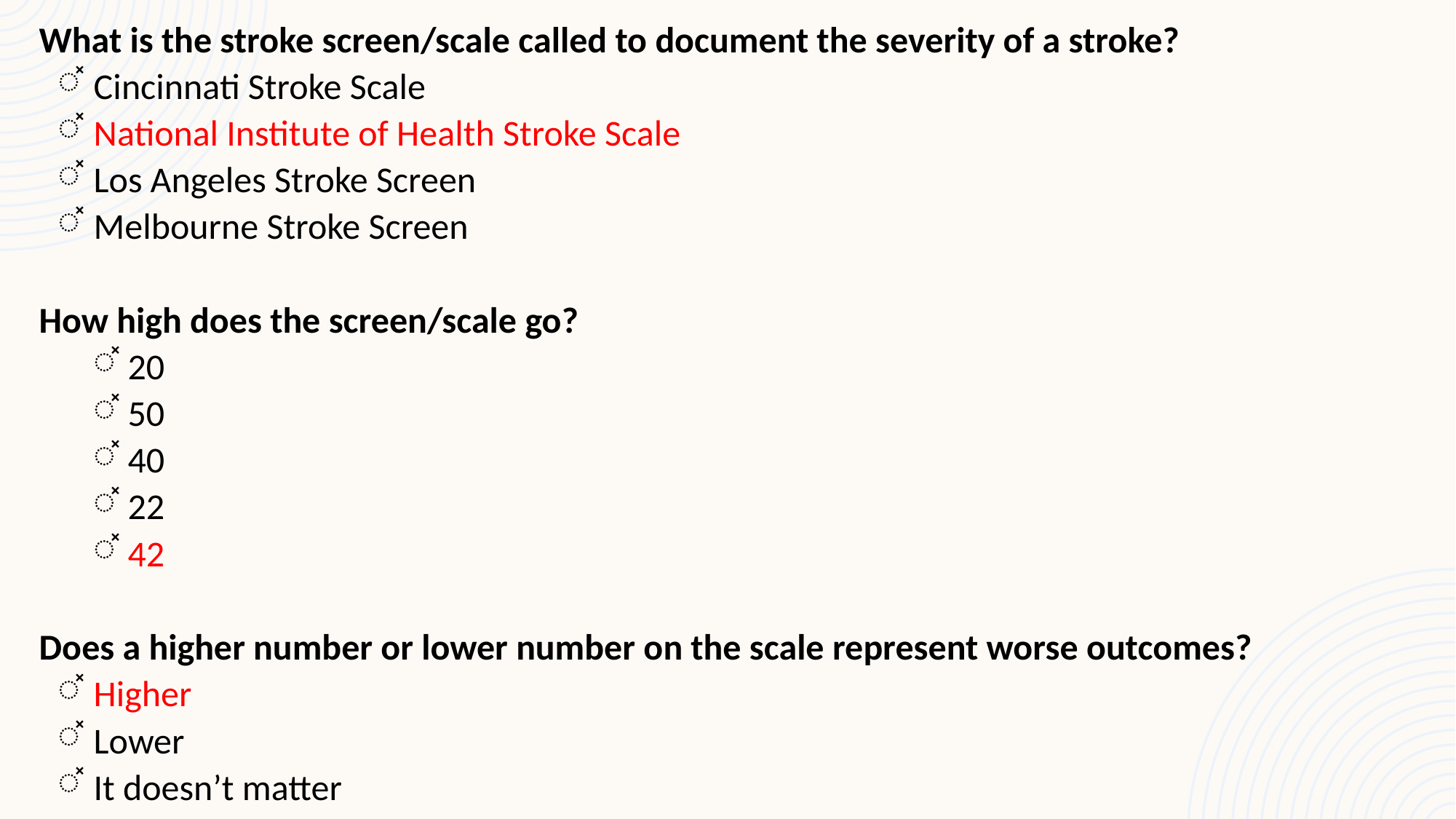

What is the stroke screen/scale called to document the severity of a stroke?
Cincinnati Stroke Scale
National Institute of Health Stroke Scale
Los Angeles Stroke Screen
Melbourne Stroke Screen
How high does the screen/scale go?
20
50
40
22
42
Does a higher number or lower number on the scale represent worse outcomes?
Higher
Lower
It doesn’t matter

## Slide 35
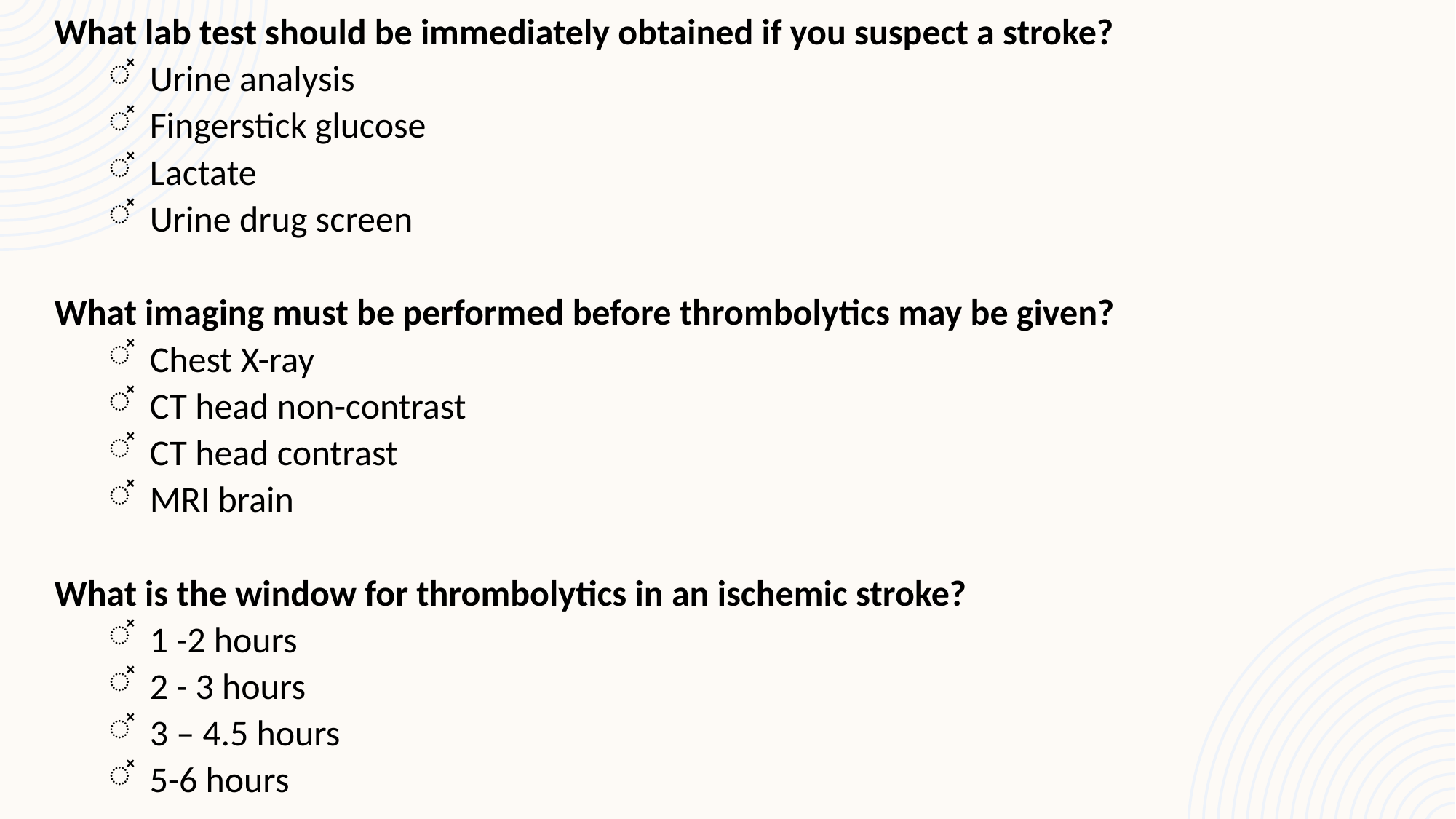

What lab test should be immediately obtained if you suspect a stroke?
Urine analysis
Fingerstick glucose
Lactate
Urine drug screen
What imaging must be performed before thrombolytics may be given?
Chest X-ray
CT head non-contrast
CT head contrast
MRI brain
What is the window for thrombolytics in an ischemic stroke?
1 -2 hours
2 - 3 hours
3 – 4.5 hours
5-6 hours

## Slide 36
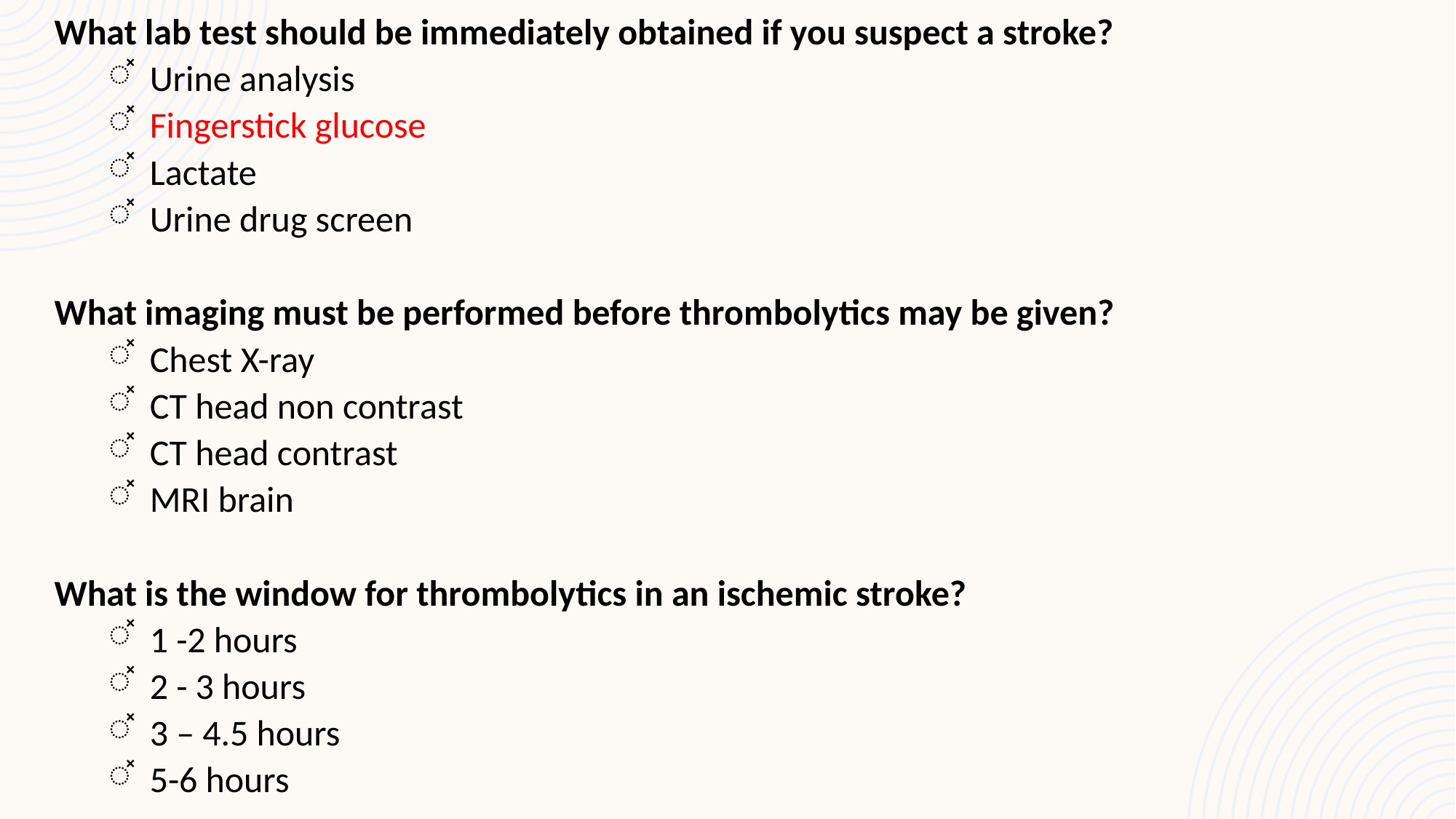

What lab test should be immediately obtained if you suspect a stroke?
Urine analysis
Fingerstick glucose
Lactate
Urine drug screen
What imaging must be performed before thrombolytics may be given?
Chest X-ray
CT head non contrast
CT head contrast
MRI brain
What is the window for thrombolytics in an ischemic stroke?
1 -2 hours
2 - 3 hours
3 – 4.5 hours
5-6 hours

## Slide 37
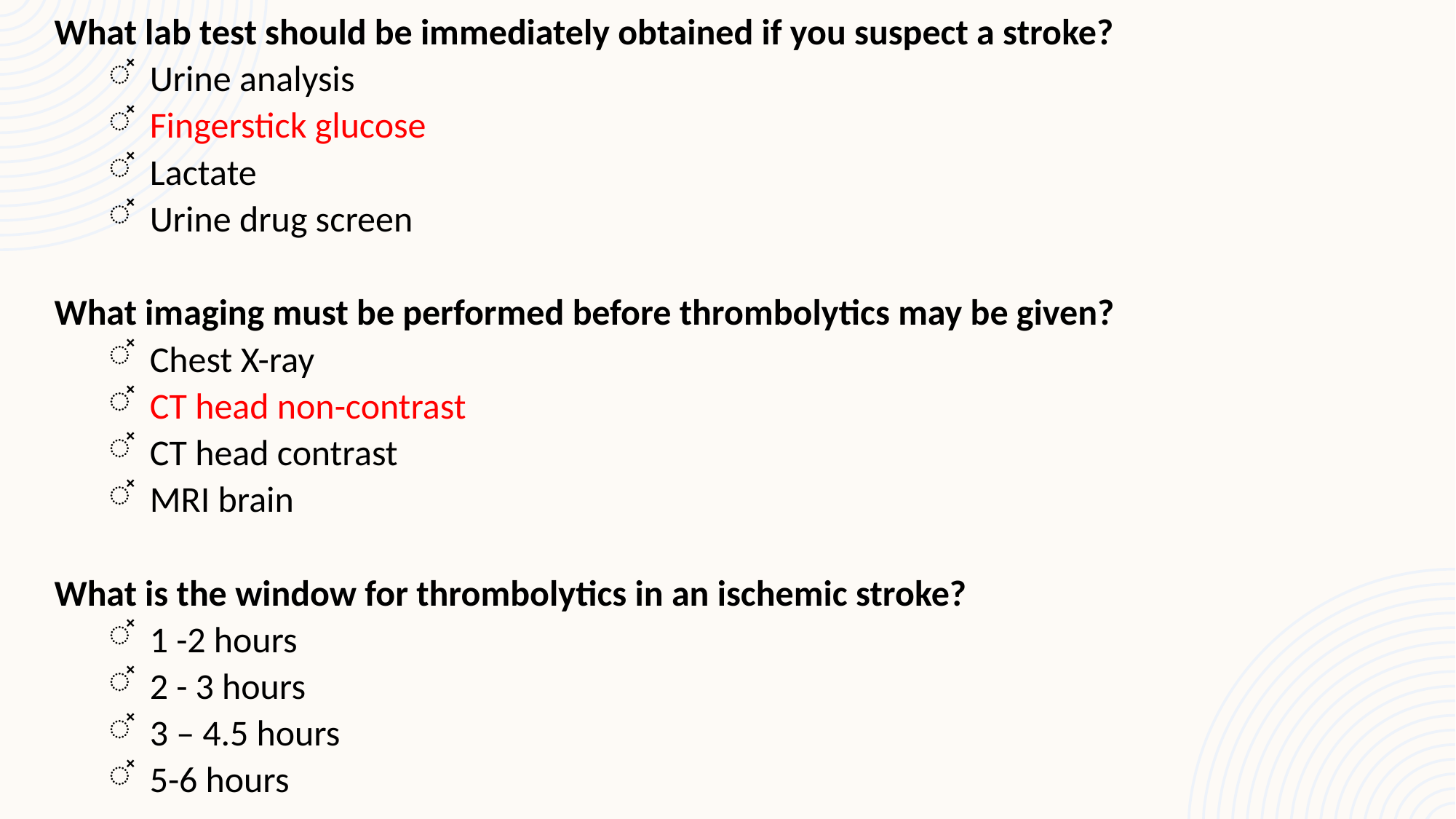

What lab test should be immediately obtained if you suspect a stroke?
Urine analysis
Fingerstick glucose
Lactate
Urine drug screen
What imaging must be performed before thrombolytics may be given?
Chest X-ray
CT head non-contrast
CT head contrast
MRI brain
What is the window for thrombolytics in an ischemic stroke?
1 -2 hours
2 - 3 hours
3 – 4.5 hours
5-6 hours

## Slide 38
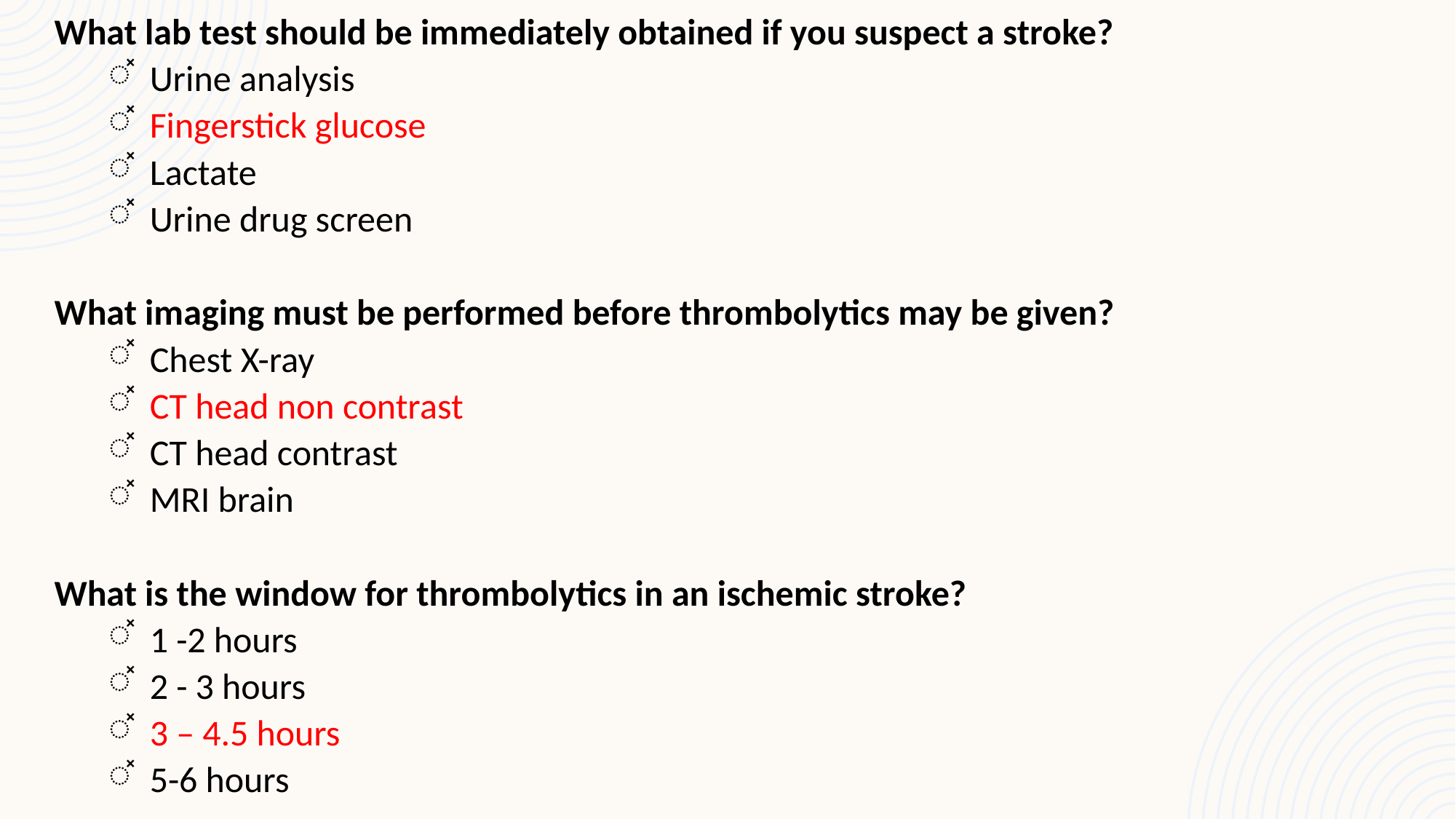

What lab test should be immediately obtained if you suspect a stroke?
Urine analysis
Fingerstick glucose
Lactate
Urine drug screen
What imaging must be performed before thrombolytics may be given?
Chest X-ray
CT head non contrast
CT head contrast
MRI brain
What is the window for thrombolytics in an ischemic stroke?
1 -2 hours
2 - 3 hours
3 – 4.5 hours
5-6 hours

## Slide 39
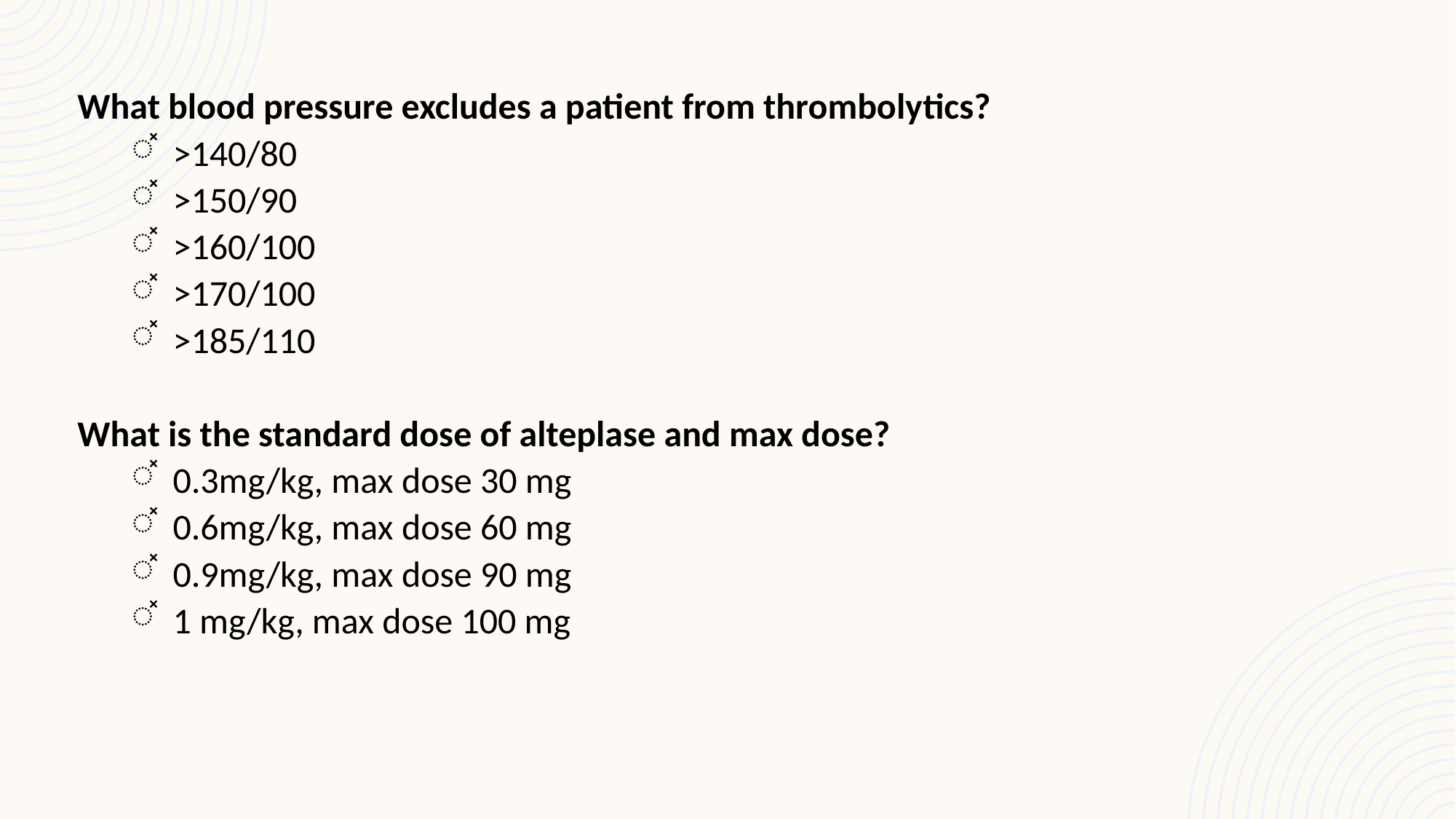

What blood pressure excludes a patient from thrombolytics?
>140/80
>150/90
>160/100
>170/100
>185/110
What is the standard dose of alteplase and max dose?
0.3mg/kg, max dose 30 mg
0.6mg/kg, max dose 60 mg
0.9mg/kg, max dose 90 mg
1 mg/kg, max dose 100 mg

## Slide 40
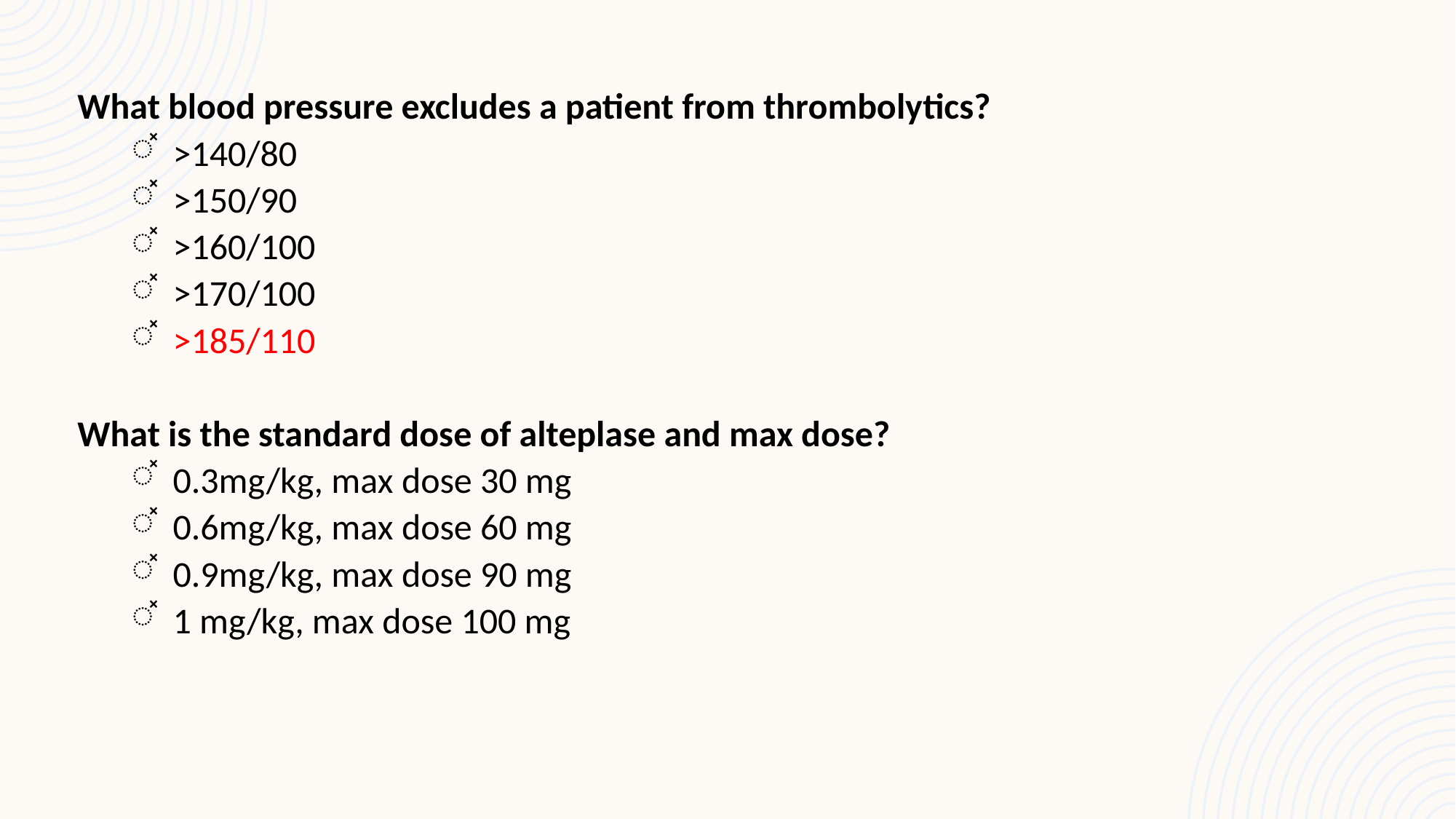

What blood pressure excludes a patient from thrombolytics?
>140/80
>150/90
>160/100
>170/100
>185/110
What is the standard dose of alteplase and max dose?
0.3mg/kg, max dose 30 mg
0.6mg/kg, max dose 60 mg
0.9mg/kg, max dose 90 mg
1 mg/kg, max dose 100 mg

## Slide 41
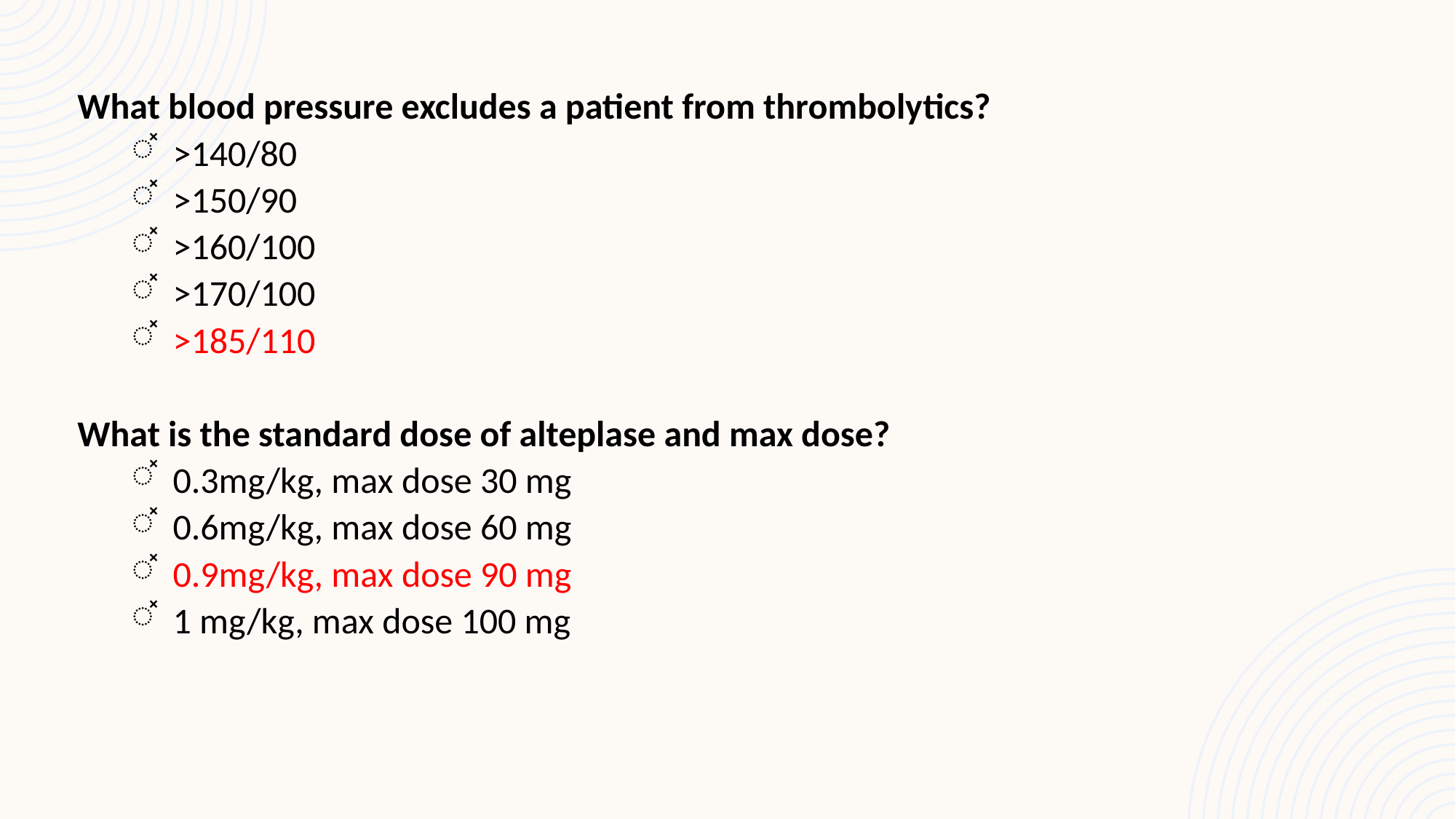

What blood pressure excludes a patient from thrombolytics?
>140/80
>150/90
>160/100
>170/100
>185/110
What is the standard dose of alteplase and max dose?
0.3mg/kg, max dose 30 mg
0.6mg/kg, max dose 60 mg
0.9mg/kg, max dose 90 mg
1 mg/kg, max dose 100 mg

## Slide 42
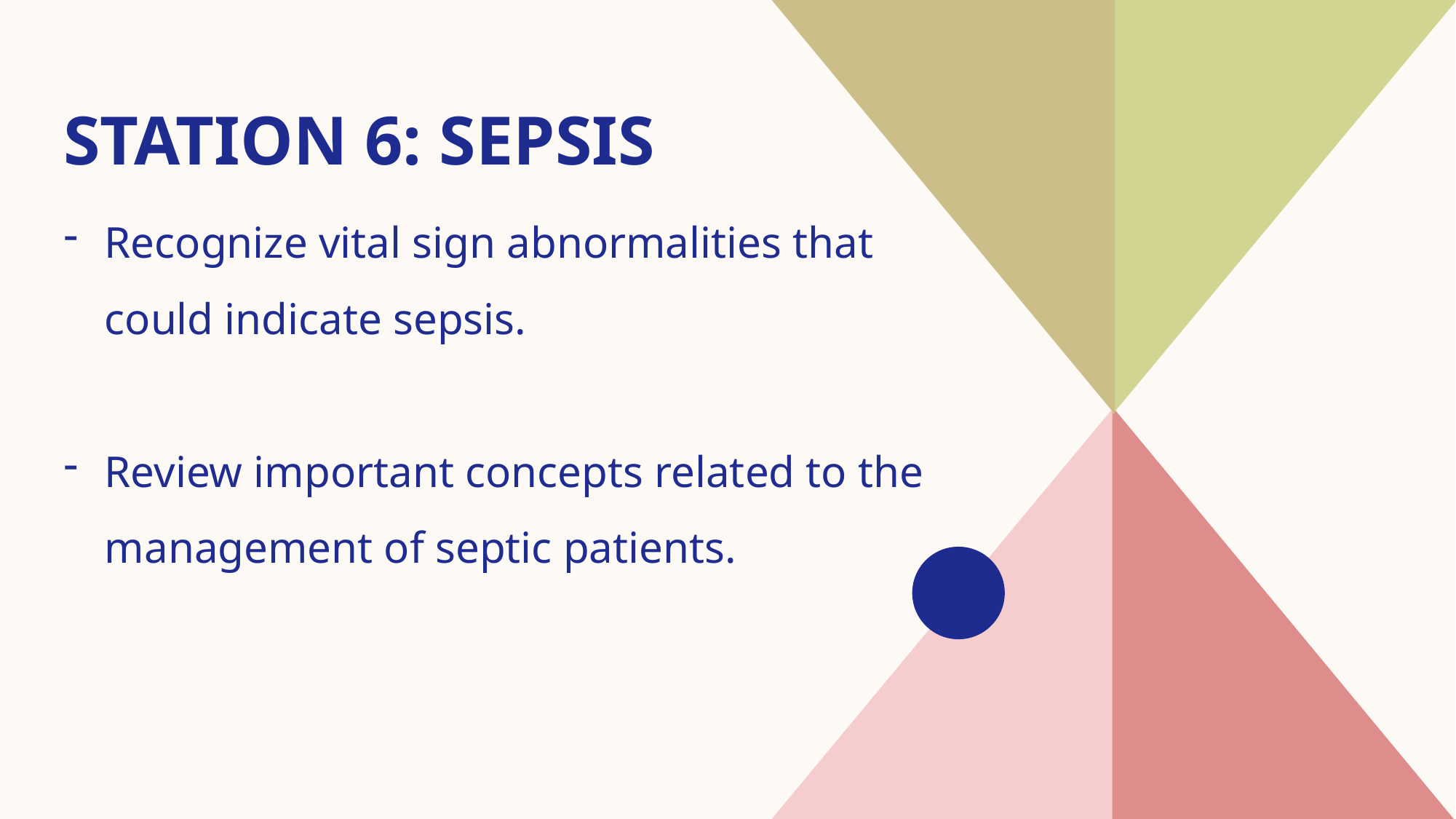

# Station 6: Sepsis
Recognize vital sign abnormalities that could indicate sepsis.
Review important concepts related to the management of septic patients.

## Slide 43
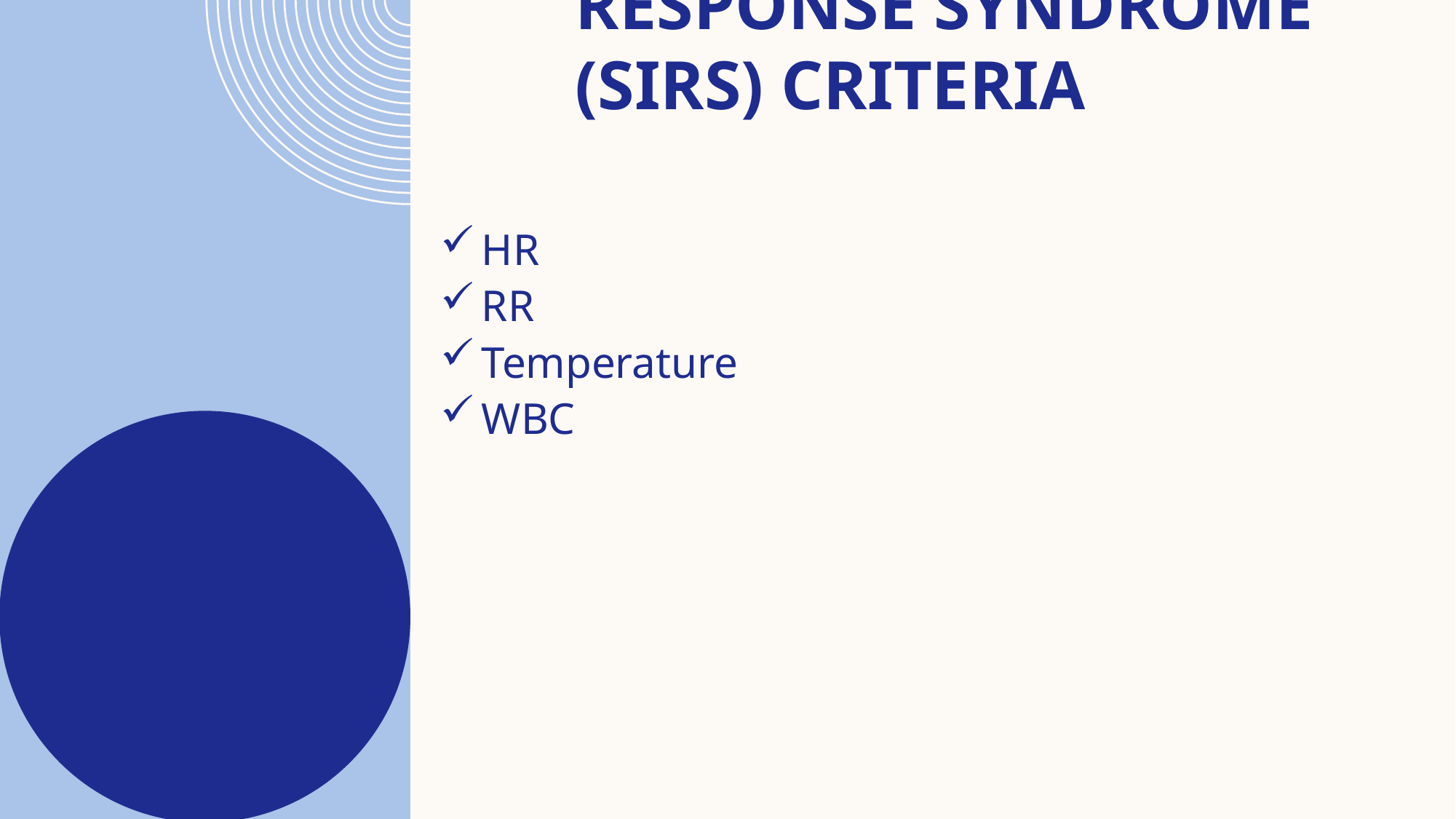

# Systemic Inflammatory Response Syndrome (Sirs) criteria
HR
RR
Temperature
WBC

## Slide 44
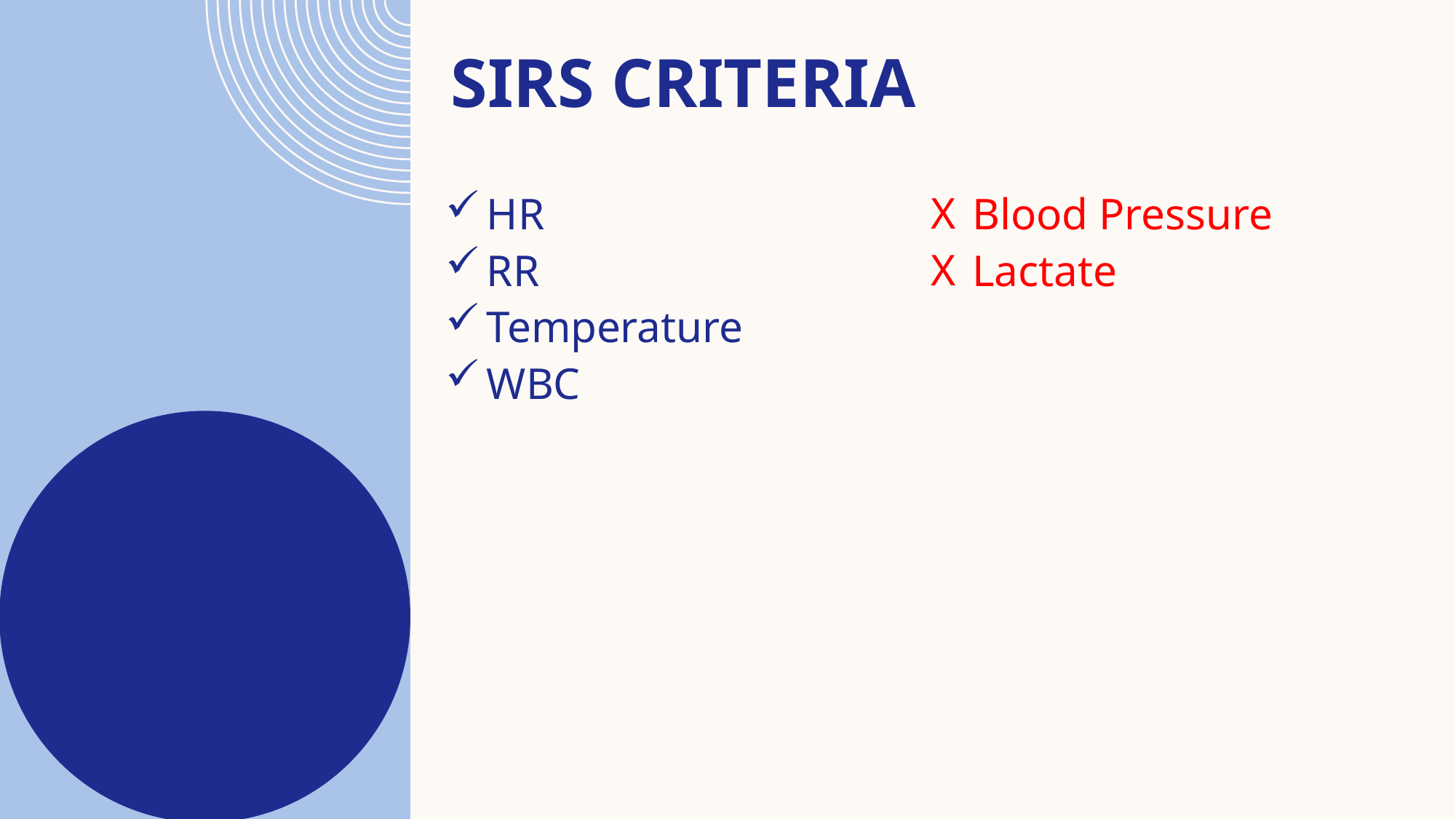

# Sirs criteria
HR
RR
Temperature
WBC
Blood Pressure
Lactate

## Slide 45
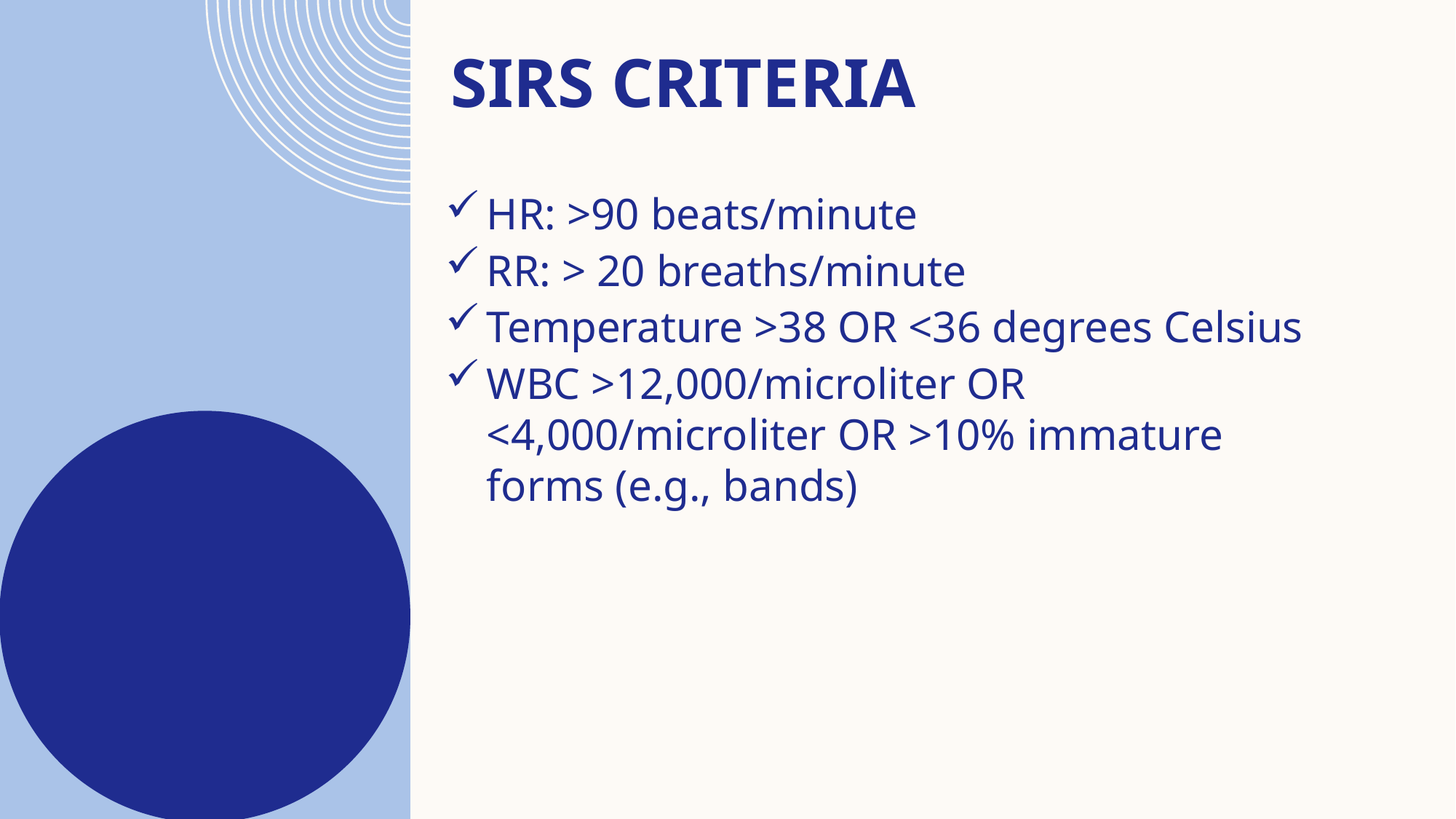

# Sirs criteria
HR: >90 beats/minute
RR: > 20 breaths/minute
Temperature >38 OR <36 degrees Celsius
WBC >12,000/microliter OR <4,000/microliter OR >10% immature forms (e.g., bands)

## Slide 46
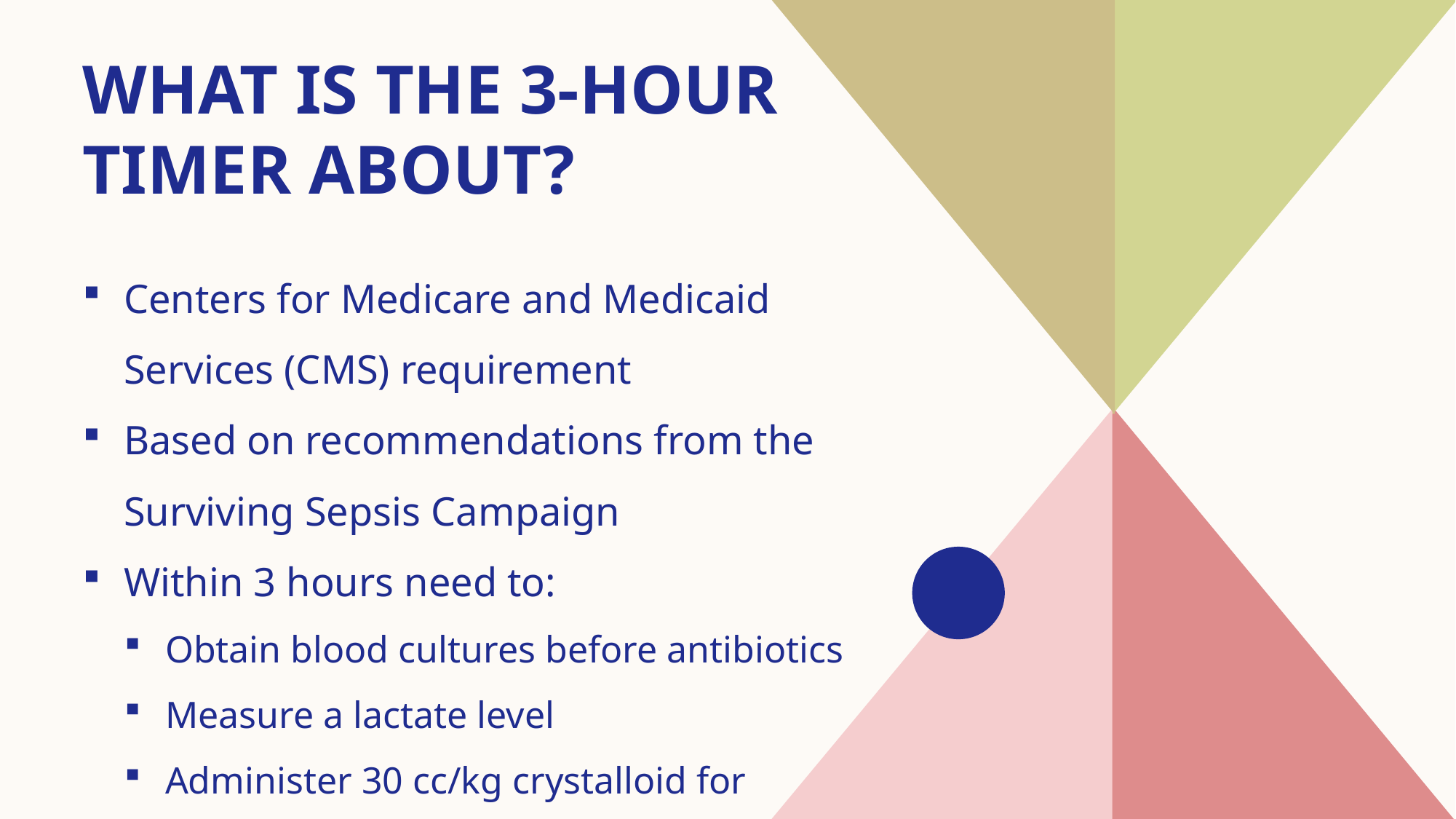

# What is the 3-hour timer about?
Centers for Medicare and Medicaid Services (CMS) requirement
Based on recommendations from the Surviving Sepsis Campaign
Within 3 hours need to:
Obtain blood cultures before antibiotics
Measure a lactate level
Administer 30 cc/kg crystalloid for hypotension
Administer empiric broad spectrum antibiotics

## Slide 47
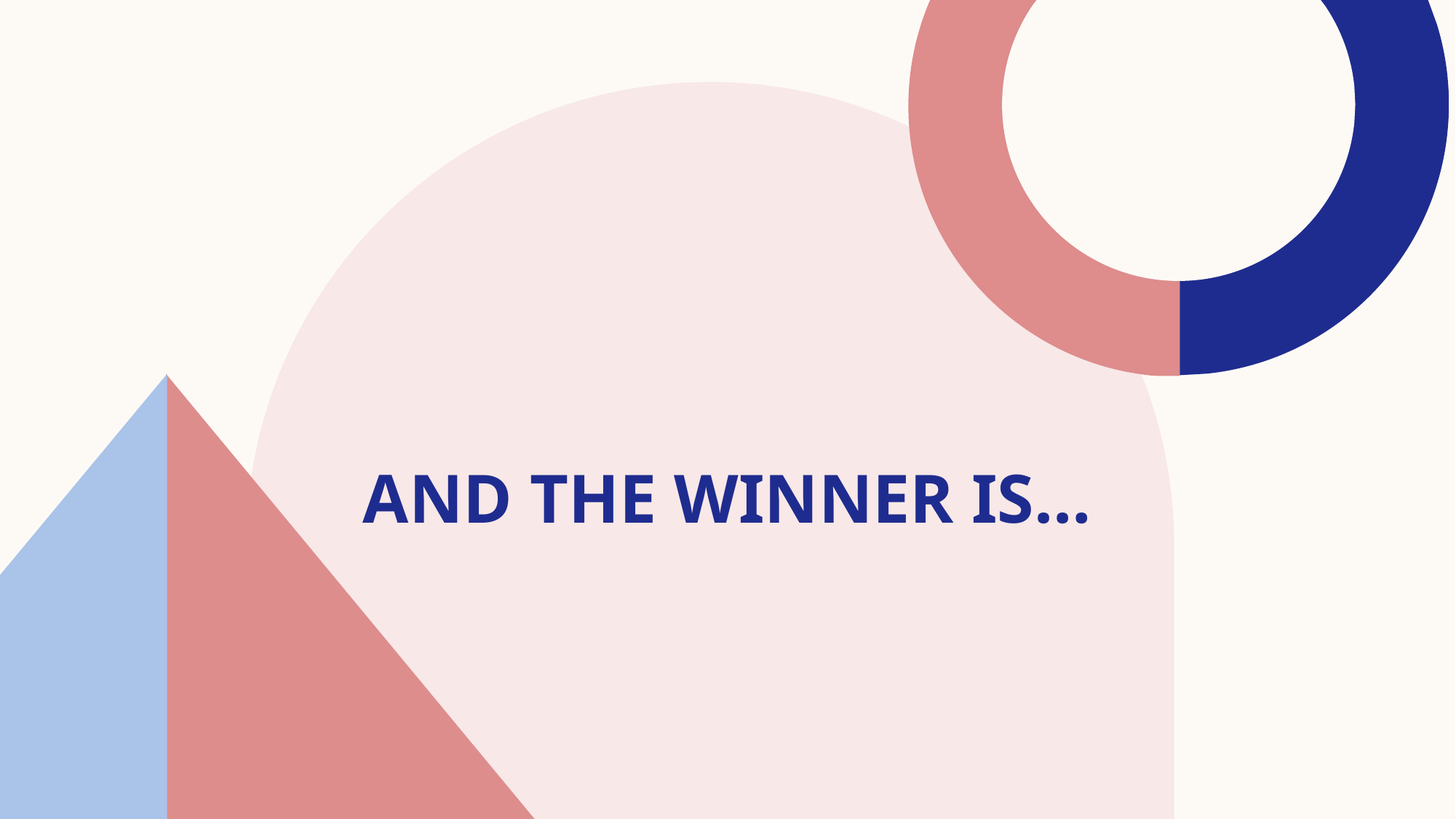

# And the winner is…

## Slide 48
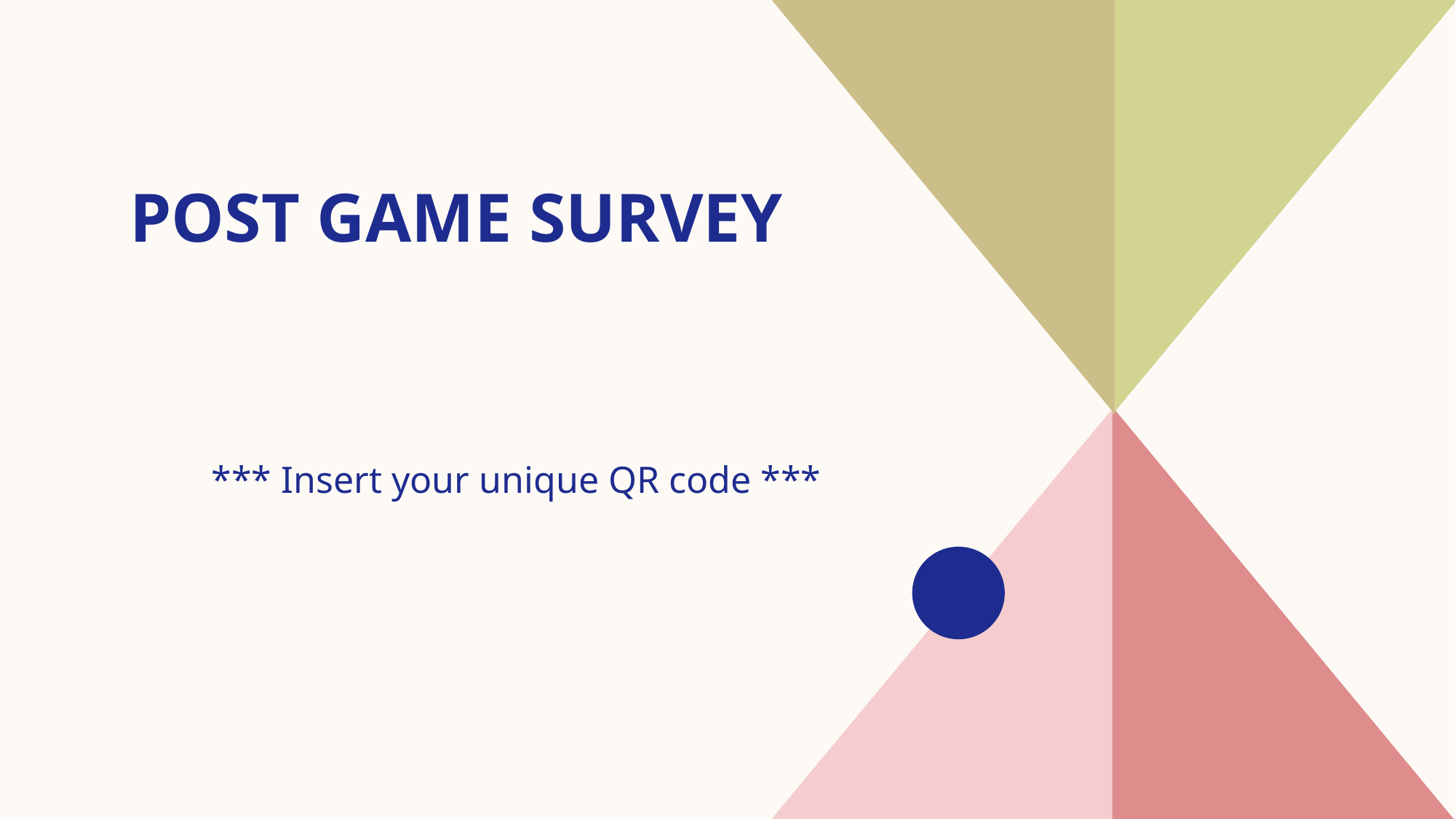

# Post game survey
*** Insert your unique QR code ***

## Slide 49
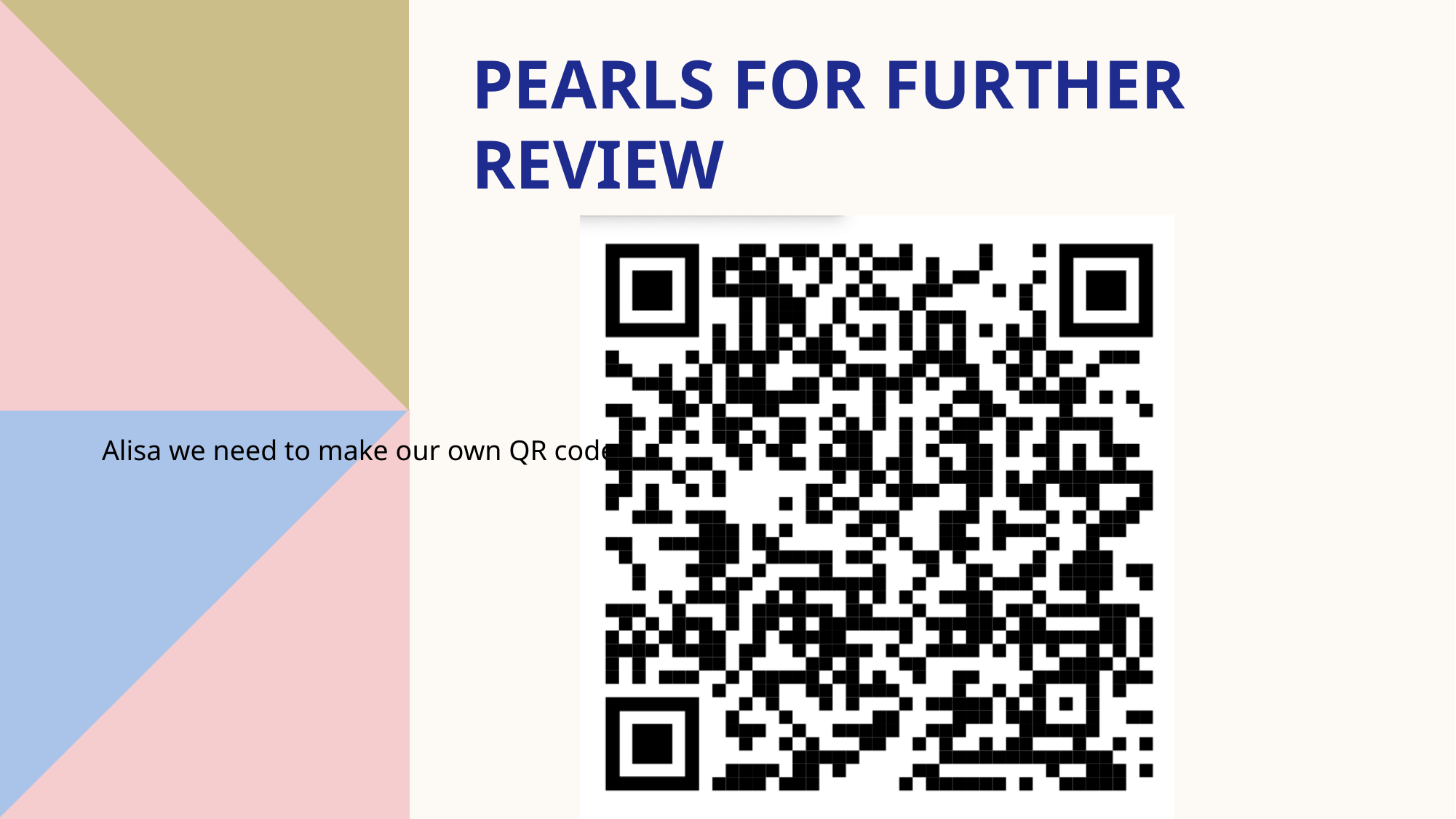

# Pearls for further review
Alisa we need to make our own QR code
